# Using web data to improve surveillance for heat sensitive health outcomes

**DOI:** 10.1186/s12940-019-0499-x

**Published:** 2019-07-09

**Authors:** Jihoon Jung, Christopher K. Uejio, Chris Duclos, Melissa Jordan

**Affiliations:** 1 Department of Geography, Florida State University, 113 Collegiate Loop, Tallahassee, FL 32306 USA; 2 Florida Department of Health, 4052 Bald Cypress Way, Tallahassee, FL 32399 USA

**Keywords:** Heat wave, Extreme heat, Public health, Surveillance system, Twitter, Google search, Social media

## Abstract

**Background:**

Elevated and prolonged exposure to extreme heat is an important cause of excess summertime mortality and morbidity. To protect people from health threats, some governments are currently operating syndromic surveillance systems. However, A lack of resources to support time- and labor- intensive diagnostic and reporting processes make it difficult establishing region-specific surveillance systems. Big data created by social media and web search may improve upon the current syndromic surveillance systems by directly capturing people’s individual and subjective thoughts and feelings during heat waves. This study aims to investigate the relationship between heat-related web searches, social media messages, and heat-related health outcomes.

**Methods:**

We collected Twitter messages that mentioned “air conditioning (AC)” and “heat” and Google search data that included weather, medical, recreational, and adaptation information from May 7 to November 3, 2014, focusing on the state of Florida, U.S. We separately associated web data against two different sources of health outcomes (emergency department (ED) and hospital admissions) and five disease categories (cardiovascular disease, dehydration, heat-related illness, renal disease, and respiratory disease). Seasonal and subseasonal temporal cycles were controlled using autoregressive moving average-generalized autoregressive conditional heteroscedasticity (ARMA-GARCH) and generalized linear model (GLM).

**Results:**

The results show that the number of heat-related illness and dehydration cases exhibited a significant positive relationship with web data. Specifically, heat-related illness cases showed positive associations with messages (heat, AC) and web searches (drink, heat stroke, park, swim, and tired). In addition, terms such as park, pool, swim, and water tended to show a consistent positive relationship with dehydration cases. However, we found inconsistent relationships between renal illness and web data. Web data also did not improve the models for cardiovascular and respiratory illness cases.

**Conclusions:**

Our findings suggest web data created by social medias and search engines could improve the current syndromic surveillance systems. In particular, heat-related illness and dehydration cases were positively related with web data. This paper also shows that activity patterns for reducing heat stress are associated with several health outcomes. Based on the results, we believe web data could benefit both regions without the systems and persistently hot and humid climates where excess heat early warning systems may be less effective.

**Electronic supplementary material:**

The online version of this article (10.1186/s12940-019-0499-x) contains supplementary material, which is available to authorized users.

## Introduction

Elevated and prolonged exposure to extreme heat is an important cause of excess summertime mortality and morbidity. During the European heat wave in 2003, large increases of excess mortality and morbidity were reported in England [1, 2], Netherlands [3], France [4–6], Italy [7], Switzerland [8], and Spain [9]. Many other articles have also discussed the negative impact of extreme heat events on public health [10–14].

There are multiple societal and environmental processes that produce variations in extreme heat resilience. First, behavioral patterns or habits could increase the risk of heat-related health problems. For example, Kilbourne et al. [15] showed that reducing physical activities and drinking extra liquid during heat waves can decrease the risk of heatstroke. Many studies also suggested that opening windows or using air conditioning can reduce the severity of heat-related morbidity [16–20].

Second, multiple studies suggest physiological adaptation (acclimatization) increases heat exposure tolerance. Acclimatization can be defined as the human body’s increased ability to cope with heat stress due to previous heat exposure [21]. When exposed to heat, the body’s renal and cardiovascular systems improve sodium retention, increase renal glomerular filtration rate, and enhance cardiovascular performance to tolerate the excessive heat [22, 23]. Third, demographic (e.g. age, sex, race), socioeconomic (e.g. deprivation), and institutional factors (e.g. energy assistance program) influence personal heat vulnerability [24–27].

The U.S. Centers for Disease Control and Prevention (CDC) initiated BioSense in late 2003 to support early detection of emerging public health threats across the nation [28]. Many states, counties (e.g. Miami-Dade, FL; San Diego County, CA; Santa Clara, CA), and cities (e.g. New York City, Boston, Washington, D.C.) currently run syndromic surveillance systems [29]. Another syndromic surveillance system, called Electronic Surveillance System for the Early Notification of Community-based Epidemics (ESSENCE), now monitors more than 300 military treatment facilities worldwide [30]. The barriers to establishing syndromic surveillance systems include a lack of resources to support time- and labor-intensive diagnostic and reporting processes, delay of official government data and results, and complexed or expensive procedures to gather health data from various sources, etc. [31, 32].

Social Network Services (SNSs) may improve upon the current syndromic surveillance systems by directly capturing people’s individual and subjective thoughts and feelings during heat waves. Some articles investigated how SNSs may reflect extreme heat sentiments. Austin [33] and Baylis [34] found a negative relationship between heat exposure and tweets containing mood-related keywords. This relationship was further refined and studied by Jung and Uejio [35] by focusing on theme-specific-tweets containing “AC” and “heat.” They found a strong positive association between temperature and tweets in Atlanta, Los Angeles, and New York City. Collectively, the papers suggest that SNSs could help jurisdictions without syndromic surveillance systems by providing accessible and cost-effective real-time data. Based on these advantages, SNSs may be helpful for both regions without these systems and persistently hot and humid climates where excess heat early warning systems may be less effective.

The goal of this study is to examine the relationship between social media messages/web search results and health outcomes, and ultimately improve upon models that only consider temperature. We hypothesized that more frequent Twitter tweets or Google search results discussing heat-related keywords are positively associated with health outcomes. These relationships were investigated with ARMA-GARCH and GLMs to control for seasonal and subseasonal trends in the data.

## Methodology

### Research area

**Fig. 1:**
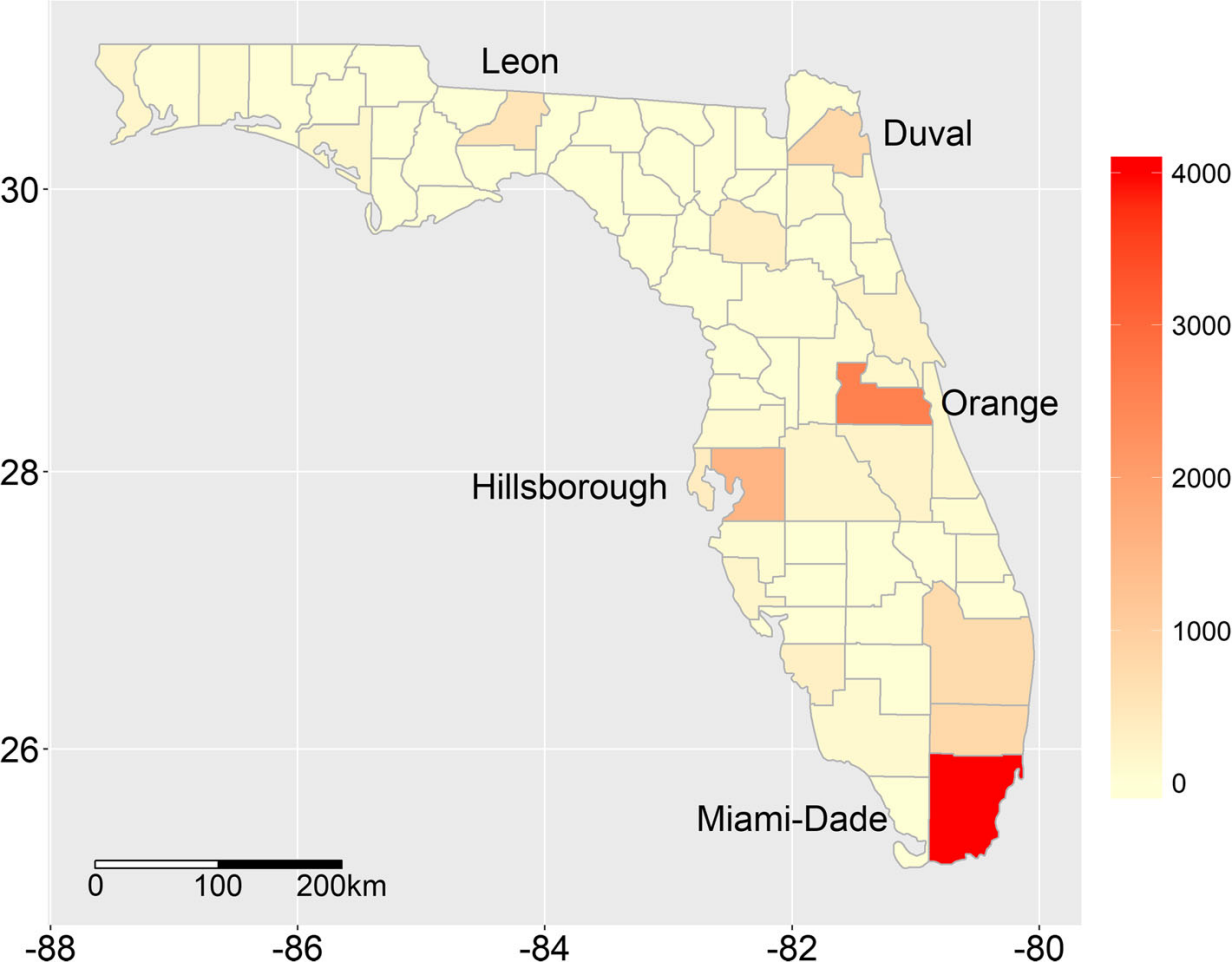
The number of Twitter tweets by county

We selected five counties in Florida, U.S. with the largest number of total validated Twitter tweets collected from the summer of 2014 (Fig. 1). There were 3991 (Miami-Dade), 2575 (Orange), 1549 (Hillsborough), 831 (Duval), and 556 (Leon) tweets. Additional file 1: Table S1 briefly summarizes the demographic characteristics of each county in 2014 using data from the U.S. Census Bureau’s American Community Survey [36]. Miami-Dade County is the most populated while Leon County is the least populated study area county. The median ages of the five counties range from 30.3 (Leon) to 39.4 (Miami-Dade). Miami-Dade and Duval County have the highest percentage of people over age 65 and people under 5 years old respectively. The proportion of females tends to be higher than males throughout the five counties. In terms of race, Anglo Americans represent from 61.8 (Duval) to 78.0% (Miami-Dade) and African Americans from 17.5 (Hillsborough) to 31.5% (Leon) of the population. Except for Miami-Dade County (66.6%), Hispanics comprise less than 30% of the population in the remaining counties.

### Data and analysis

#### Twitter

Twitter is a SNS focusing on text-based messages to communicate with other people. Users can post their feelings, thoughts, and any information up to 140 characters in length (280 characters after 11/2017) in each tweet. They can also redistribute other users’ tweets (i.e. retweet or RT), follow and/or be followed by other users. Followers who subscribe to others’ Twitter accounts can receive all their messages in real time. The user base is currently expanding from individuals to companies, associations, and governments. More than 300 million people actively use the service worldwide.

We respectively accessed the Twitter Search and Streaming Application Programming Interface (API) using the twitteR and streamR packages provided in the R statistical computing and analysis program [37]. We collected tweets for 181 days from May 7 to November 3, 2014. The data from the period June 8 to 10, September 14 to 20, and October 9 were missing due to technical problems.

We constructed two themes from Twitter; AC and heat. Table 1 describes the 3 and 42 Twitter keywords that respectively comprise the AC and heat themes. AC is one of the most effective and widespread measures to adapt to heat exposure and mitigate negative health impacts [38–42]. We explored possible keywords from historical tweets using the Twitter Search API. Through this process, we developed a “heat” theme that contained “heat” plus another term such as a place (e.g. car, home, office, school), time (e.g. May, summer), symptoms (e.g. heat exhaustion, heatstroke, hyperthermia, and sunstroke), adjective (e.g. unbearable, dry), a related noun (e.g. warning, watch) or a pronoun (e.g. this). We also considered possible typographic errors (e.g. heet, sun-strok). More detailed information on data collection procedures can be found in Jung and Uejio [35].

**Table 1:** Two main themes and forty-five keywords were used for collecting data. Numbers in parentheses represent the total number of keywords

Theme	Keywords
AC (3)	A/C, Air conditioning, HVAC
Heat (42)	Car heat, Dry heat, Fan heat, Heat august, Heat damn, Heat extremely, Heat fuck, Heat fucking, Heat home, Heat index, Heat intensifies, Heat july, Heat june, Heat killing, Heat may, Heat office, Heat related, Heat-related, Heat school, Heat september, Heat sun, Heat unbearable, Heat warning, Heat watch, Heat wind, Heatex, Heat-exhasution, Heatsroke, Heatstroke, Heet, Humidity heat, Hyperthermia, Melt heat, Overheat, Overheated, Sleep heat, Steam heat, Summer heat, Sun-strok, Sunstroke, This heat, Unbearable heat

#### Google search

Google search data can provide complementary information to Twitter. While Twitter data are only freely accessible over the preceding seven days (Twitter Search API) or in real time (Twitter Streaming API), the Google search website provides historic search data from 2004 to present on a daily or weekly basis. However, Google only reports a relative search popularity index. Regardless of the total number of searches, every keyword’s popularity is relatively rescaled to an index that ranges from 0 to 100. This makes it difficult to directly compare keywords’ relative importance. This procedure could be problematic for less common search keywords with a larger search volume variation.

In terms of spatial resolution, Google search reports search volumes at the aggregated data and marketing association commercial boundary level (Additional file 1: Figure S1). This differs from Twitter which either reports a user’s exact location (i.e. latitude and longitude) or general city of origin. In this study, we used five commercial areas which encompass Duval (Duval County), Leon & Panama City (Leon County), Miami-Dade & Ft. Lauderdale (Miami-Dade County), Orlando & Daytona Beach & Melbourne (Orange County), and Tampa & St. Petersburg (Hillsborough County).

We collected Google search data using the gtrendsR package provided in the R statistical computing and analysis program. This package provided the interface for retrieving and displaying the Google search results. For this research, we collected daily Google search volumes for 181 days from May 7 to November 3, 2014. Each Google search term’s volumes were automatically rescaled with a value from 0 to 100.

In general, people use search engines (e.g. Google) to obtain information rather than to express their feelings or thoughts. We, thus, separately selected 11 specific Google search themes which include AC repair, beer, drink, heat exhaustion, heat stroke, hot weather, park, pool, swim, tired, and water. In this process, we chose themes which are related to weather, health, recreational information, and adaption method. The recreational information corresponds to previous studies of heat risk factors. Staying hydrated (“drink” and “water”) decreases while alcohol consumption (“beer” and “drink”) increases the risk of heat-related illnesses [43–45]. Moving to a cooler environment can ameliorate heat stress [46]. We also selected “park,” “pool,” and “swim” which are commonly used to adapt to extreme heat [47–49]. In addition, adaptation method focused on “AC repair.” AC is generally regarded as one of the most important adaptions during heat waves [38–42]. Thus, a total of 13 terms (2 themes from Twitter and 11 themes from Google) were used to find the relationship between web data and health outcomes.

#### Weather data

To examine excessive heat, we used daily observation data, Global Summary of the Day, provided by the US National Oceanic and Atmospheric Administration National Centers for Environmental Information. We selected all weather stations within each research area that missed less than 10% of observations over the study period. Next, we created a research-area-wide average of temperature (maximum, minimum, average) and humidity. We also created a discomfort index (DI) from temperature and relative humidity using Eq. (1) [50].

1 $$ \mathrm{DI}=\mathrm{T}-0.55\ast \left(1-0.01\ast \mathrm{RH}\right)\ast \left(\mathrm{T}-14.5\right) $$

Where T is the air temperature (°C) and RH is the relative humidity (%). After sensitivity testing, we selected maximum temperature for the principal metric, which tended to have the best model fit with health data (lowest Akaike information criterion (AIC)). The descriptive summary of weather conditions and AICs for each research area is tabulated in Additional file 1: Table S2.

#### Health data

ED and hospitalization data were collected by the Florida Agency for Health Care Administration (AHCA). AHCA is Florida’s principal health policy and planning agency that manages Medicaid, health care facility licensing, and distribution of health care data. We selected five broad heat sensitive disease or symptom classes from the International Classification of Diseases Clinical Modification (ICD-CM, 9th revision): cardiovascular disease (390–459), dehydration (276.51), heat-related illness (992), renal disease (5845–5849), and respiratory disease (460–466, 470–496, 510–519) [11, 51, 52]. The records report a patient’s primary diagnoses, demographics, and billing information (e.g. ZIP Code). In this study, we separately analyze ED and hospitalization counts due to their different weekly and seasonal cycles.

### Twitter data preprocessing

We briefly summarize the data preprocessing and validation steps that are outlined in detail in Jung and Uejio [35]. Preprocessing removed all retweets, irrelevant tweets, and tweets posted by users who used the same keywords more than 10 times (to avoid possible advertisements) in the research period. After this process, the proportion of relevant heat-related tweets was 89.5% for the AC and heat themes. We then accessed or inferred the spatial location of each tweet at the county-level analysis. Each tweet’s metadata had specific and general spatial data with different strengths and weaknesses. Only 4 to 6% of tweets reported the user’s precise latitude and longitude (e.g. 30.418842, − 84.268449). On the other hand, approximately 70% of all tweets contained a more uncertain self-reported location field (e.g. Tallahassee, Florida). To maximize data availability, we assigned the city’s geographic center to meaningful location fields. We verified the accuracy of this process by measuring the Euclidean distance between the inferred city center and specific latitude and longitude for the subset of tweets that contained both pieces of information. Previous validation effort found almost 80% of the tweets were made within 100 km of the user’s location field [35].

### Statistical analysis

First, exploratory Pearson’s correlation coefficients were used to check for potential multicollinearity between maximum temperature and Twitter and Google search volumes. In a statistical model, very strong multicolinearity may inflate the beta coefficient variance and even change the signs of the coefficient. Second, the analysis used two types of statistical models, ARMA-GARCH and GLMs. The analysis initially used GLMs with the appropriate statistical distribution families. If the model exhibited any significant residual temporal autocorrelation, we used ARMA-GARCH models to control unexplained autocorrelation. Conventional autocorrelation (ACF) and partial autocorrelation functions (PACF) were tested for significant residual autocorrelation.

Separate statistical models analyzed each health outcome category as a dependent variable (i.e. cardiovascular disease, dehydration, heat-related illness, renal disease, and respiratory disease); the day of week, maximum temperature, and web data were independent variables. In the analysis, we compared three increasingly complex statistical models. The initial or null model only considered the day of week. The second model added maximum temperature to the first model. The third group of models separately added one web search term to the second model. We used AIC to compare the model performance.

GLMs are a generalization of ordinary least squares regression. They can flexibly analyze dependent variables following different statistical distributions using a range of statistical families and link functions. We used three GLM models depending on the distribution of health outcomes: Gaussian, negative binomial, and logit. The Gaussian distribution uses an identity link function. The negative binomial distribution and 1/x link function models overdispersed counts (variance > mean). Lastly, logistic regression using logit link models examines a binary dependent variable. Most of the case counts followed normal or negative binomial statistical distributions. Relatively rare health outcomes such as heat-related illness were reclassified into days with one or more versus no cases and analyzed with logistic regression.

ARIMA (m, d, n) is a time series model that is usually used to forecast future values based on a linear relationship with past values [53]. The model considers the process of autoregressive order m, the degree of differencing d, and moving average order n. We added exogenous explanatory variables in this model, which is called ARIMAX, to find the relationship between dependent variable (health outcome) and exogenous variables (temperature and web data). This model assumes constant mean (stationarity) and variance (homoscedasticity) of data. In addition, the residuals are also expected to be randomly distributed with a mean of zero, constant variance, and follow a normal distribution.

ARMA-GARCH (p, q) is a more flexible time series model used to measure non-constant health outcome variance and unexplained autocorrelation [54]. We modeled the mean equation with an ARMA process and the variance equation with a GARCH process where *P* is the autoregressive and *q* the moving average order. We also added exogenous explanatory variables in this model to find the association between dependent variable (health outcome) and exogenous variables (temperature and web data). The GARCH model uses a non-constant conditional variance (heteroscedasticity) which reverts back to a constant unconditional variance (homoscedasticity). The conditional variance is explicitly modeled using the squared values of preceding observations and variances [55].

To identify and select the best model, we first used 1) a time series plot, 2) ACF and PACF of the data, and 3) ACF and PACF of the squared data. Next, we tested and compared the model fit (AICs) of all possible models up to four orders of AR and MA. If the models had similar goodness of fit (AICs difference < 1, or area under the receiver operating characteristics curve [AUC] < 0.01), we selected the most parsimonious model. In a similar vein if the AICs difference between models were higher than 1, we considered it as model improvement. Lastly, we rechecked the statistical assumptions of the final model such as 1) no autocorrelations of the residuals (ACF/PACF) and 2) standardized residuals tests.

## Results

Table 2 summarizes the demographic characteristics of each county’s cases. Similar to each county’s population size, Miami-Dade County had the highest number of patients and Leon County had the lowest. Among the five study counties, cardiovascular disease was the most common illness, followed by respiratory, renal, dehydration, and heat-related illness. Respiratory disease and heat-related illness had more ED than hospitalization cases while the reverse was true for dehydration and renal disease. Cardiovascular disease had a similar number of ED and hospitalization cases. In terms of age, hospitalized patients tended to be older than ED patients for all health outcomes. The median age of respiratory disease, heat-related illness, and dehydration ED patients was between 20 and 30 years old, whereas the median cardiovascular and renal disease patient age was > 50. We found a disproportionate disease burden by sex for some disease categories. There were proportionately more male than female heat-related illness and renal disease cases while the opposite was true for cardiovascular disease, respiratory disease, and dehydration. We also compared the patient demographics to countywide demographics from the 2014 U.S. census [36]. In terms of race, we found proportionately more African-Americans and fewer Anglo Americans ED and hospitalization cases compared to their population size. This trend was especially apparent in cardiovascular, respiratory, renal, and heat-related illness ED cases.

**Table 2:** Demographic characteristics of patients in each county (*ED* emergency department, *HSP* hospitalization, *M* male, *F* female, *W* white, *B* black, *H* Hispanic, *N-H* non-Hispanic)

County	Variable	Cardiovascular		Dehydration		Heat-related		Renal		Respiratory	
		ED	HSP	ED	HSP	ED	HSP	ED	HSP	ED	HSP
Duval	Total case (#)	39,156	38,662	2843	4040	226	45	241	9024	32,463	18,273
	Age (median, yr)	54	64	35	62	33	46	58	67	25	62
	Sex (M/F, %)	41/60	46/54	39/61	41/59	70/30	89/11	59/41	51/49	39/61	42/58
	Race (W/B/Other, %)	45/49/6	61/34/5	55/37/7	62/32/5	48/42/8	56/33/11	48/46/5	57/38/5	36/55/8	63/32/5
	Ethnicity (H/N-H, %)	4/95	3/96	5/94	4/96	4/95	11/89	3/95	3/96	6/93	3/96
Hillsborough	Total case (#)	51,731	43,848	3154	4209	259	76	283	7631	46,800	22,673
	Age (median, yr)	55	65	33	62	31	43	59	68	25	62
	Sex (M/F, %)	41/59	47/53	41/59	47/53	73/27	91/9	66/34	56/44	41/59	44/56
	Race (W/B/Other, %)	63/27/10	71/19/9	68/23/9	72/20/7	61/30/9	63/27/11	66/26/8	70/21/8	57/30/12	72/19/8
	Ethnicity (H/N-H, %)	20/80	15/84	25/75	17/83	21/79	20/80	16/83	14/85	29/71	16/84
Leon	Total case (#)	9184	6981	1210	853	49	10	109	1437	8854	3265
	Age (median, yr)	54	66	38	65	36	46	58	66	26	61
	Sex (M/F, %)	38/62	46/54	40/60	45/55	78/22	80/20	58/42	51/49	38/62	44/56
	Race (W/B/Other, %)	44/54/2	63/35/2	61/37/2	65/33/2	50/50/0	60/40/0	45/51/3	58/40/2	39/58/3	64/34/2
	Ethnicity (H/N-H, %)	2/97	2/96	2/96	2/96	0/100	0/100	1/97	2/96	3/96	2/96
Miami-Dade	Total case (#)	93,331	93,505	4490	6995	165	36	1147	16,018	73,609	41,179
	Age (median, yr)	61	69	44	69	37	52	67	74	16	68
	Sex (M/F, %)	43/57	49/51	42/59	47/53	66/34	81/19	65/35	57/43	45/55	48/52
	Race (W/B/Other, %)	69/26/5	73/21/5	78/17/4	77/18/4	56/33/9	78/11/8	70/22/7	72/23/5	68/27/4	73/21/5
	Ethnicity (H/N-H, %)	60/37	61/36	64/33	63/35	54/41	75/22	55/41	59/39	62/36	61/37
Orange	Total case (#)	42,537	40,449	2075	3235	161	32	450	7321	39,829	18,596
	Age (median, yr)	55	64	31	56	35	50	60	67	23	60
	Sex (M/F, %)	42/58	47/53	40/60	48/52	71/29	78/22	61/39	56/44	42/58	44/57
	Race (W/B/Other, %)	52/33/14	63/24/11	57/26/15	60/25/13	56/34/10	38/34/25	51/35/12	61/27/10	43/34/22	64/23/12
	Ethnicity (H/N-H, %)	26/70	21/75	30/66	22/75	23/71	22/75	21/74	18/77	36/61	22/75

Exploratory linear regression investigated potential multicollinearity between maximum temperature and web keywords (Additional file 1: Figure S2). The overall mean Pearson Coefficient was 0.35 (0.02 ~ 0.57) for heat and 0.34 (0.18 ~ 0.50) for AC. Nearly all Twitter keywords were significantly related (*p* < 0.05) to maximum temperature for all study counties except Miami-Dade County. We suspect “heat” tweets in the county might contain non-relevant reference to the Miami Heat professional basketball team. For example, some ambiguous tweets could refer either to the weather or the professional basketball team (e.g. heat!). In addition, the concurrent day’s maximum temperature exhibited the strongest relationship to Twitter data than temperature over the preceding three days. As the temporal lag increased, the Pearson’s correlation coefficient correspondingly decreased, and *p*-value increased.

For Google search keywords, the pool and tired keywords show the highest and lowest overall mean Pearson’s correlation coefficient corresponding to 0.34 and 0.05. Several Google search keywords such as park, pool, swim, and water were significantly related with maximum temperature in at least four out of five counties at the *p* < 0.05 level. Other keywords including AC repair, heat exhaustion, and heat stroke were also significantly associated in two or three out of five counties. On the other hand, beer and tired were insignificantly related. While there were some significant associations between web data and maximum temperature, the consistency of the association for both Twitter and Google, < 0.60 Pearson correlation coefficient, suggests multicollinearity would not be problematic.

### Heat-related illness

We first analyzed the relationship between heat-related illness and web data using a GLM (logit family, binary link function) due to the small number of cases. We tested three different models to see how additional independent variables improved model fit: the initial model (days of week), the second model (days of week and maximum temperature), and the third group of models (days of week, maximum temperature, and one web data term). In almost all models, heat-related illness cases didn’t exhibit a weekly cycle (Additional file 1: Table S3, S4). Only hospitalization cases in Orange showed a weak weekly cycle, having more patients on Thursday compared to Tuesday. Not surprisingly, including maximum temperature significantly improved model performance in all five counties for both ED and hospitalizations. Hillsborough County exhibited the largest AUC improvement 0.53 to 0.79 while Miami-Dade County showed the smallest from 0.67 to 0.75. Correspondingly, AICs also significantly decreased after adding maximum temperature in the model, ranging from 15.81 (Miami-Dade) to 48.15 (Duval). Heat-related illness cases tended to be significantly and positively related to the maximum temperature at the *p* < 0.05 level. The odds ratios of maximum temperature for both ED and hospitalization cases ranged from 1.19 to 1.52, which means one unit change in maximum temperature increased the odds of a heat-related illness by 19 to 52%.

**Fig. 2:**
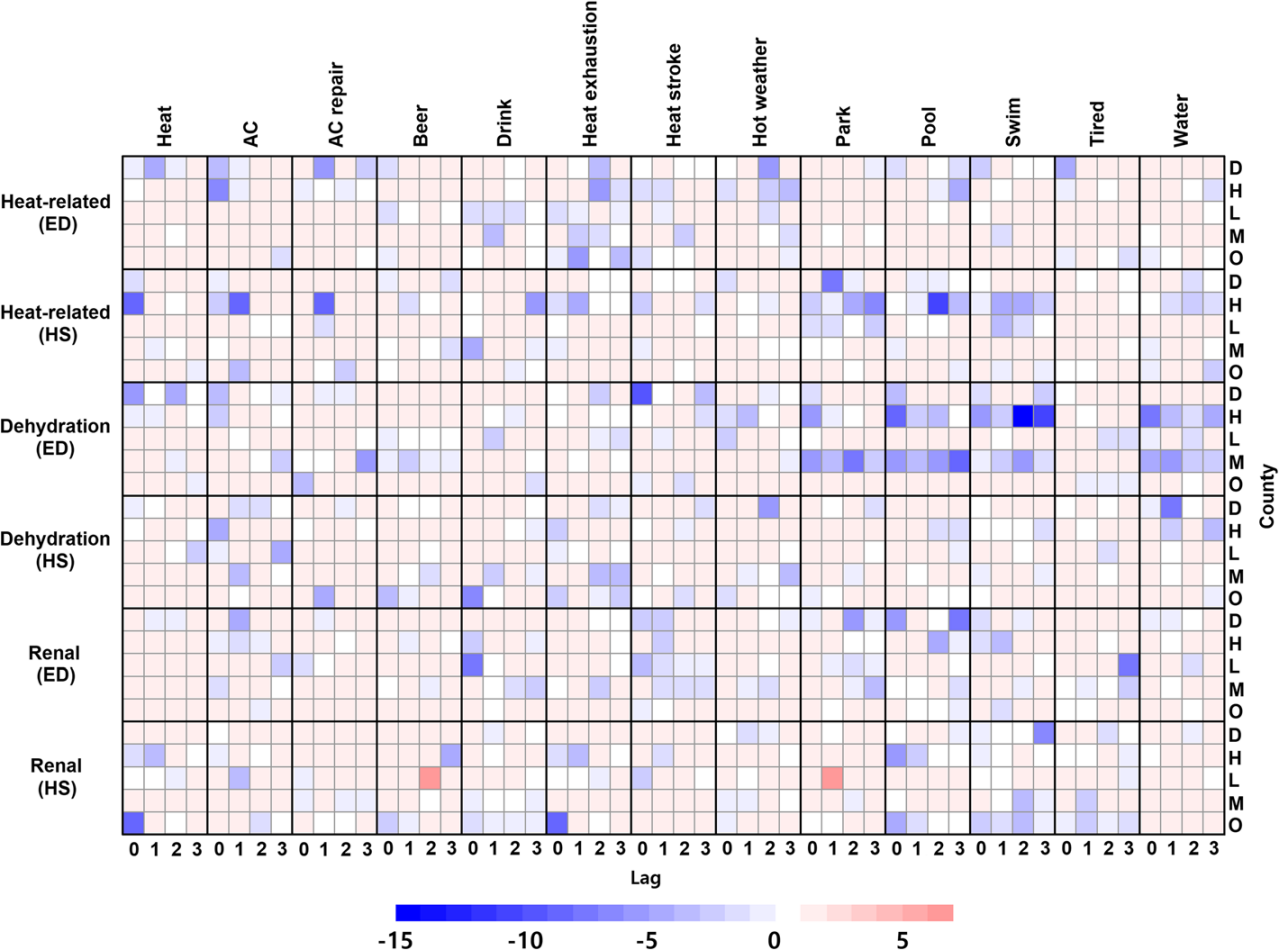
AIC changes after adding one of web data to the second model (days of week and maximum temperature). Minus (blue) means model improvement

Adding one of web data (up to 3 lag days) term modestly improved model fit (Fig. 2). At lag 0, multiple keywords including AC, beer, drink, heat exhaustion, heat stroke, hot weather, pool, swim, and tired for ED and AC, drink, heat, heat exhaustion, heat stroke, hot weather, and park for hospitalizations showed model improvement (AICs difference > 1). The largest AICs decrease was 6.17 for ED and 8.91 for hospitalizations. In particular, AC, beer, and heat stroke for ED and heat and park for hospitalization improved the models in two out of five counties. At lags 1 to 3, we also observed model improvement from AC, AC repair, drink, heat, heat exhaustion, heat stroke, hot weather, pool, swim, tired, and water for ED and AC, AC repair, beer, drink, heat exhaustion, heat stroke, park, pool, swim, and water for hospitalizations. To provide more detail, heat exhaustion and hot weather improved ED models in four out of five counties. Drink, heat stroke, and pool also improved ED models in two out of five counties. On the other hand, AC repair and park improved hospitalization models in three out of five counties. AICs decreased up to 5.89 for ED and 8.74 for hospitalization after adding one lagged web data term to the model. More details can be found from Additional file 1: Tables S5 and S6.

**Fig. 3:**
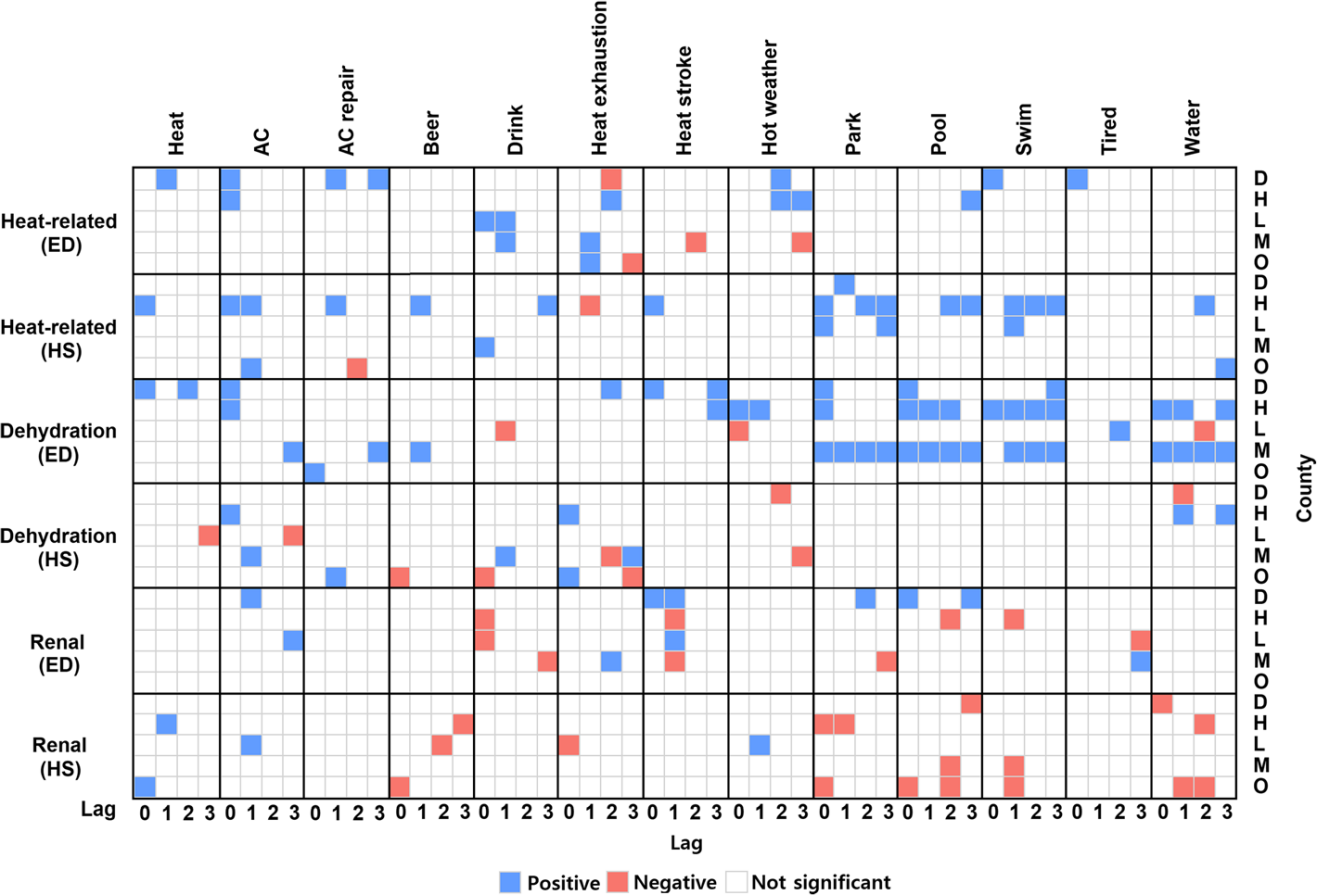
The significant beta coefficients of all web data up to 3 lags

To succinctly illustrate web search and health outcome relationships, Fig. 3 reports the direction of the significant beta coefficients (i.e. plus or minus). Overall, the web search relationships were consistent across the five counties. Heat-related illness cases showed positive associations with most of the search terms (heat, AC, drink, heat stroke, park, swim, and tired) at lag 0. We also found positive relationships between the preceding day(s) search terms for heat, AC, AC repair, beer, drink, park, pool, swim, and water and heat-related illness. Notably, AC, drink, park, and swim had positive relationships with the number of heat-related illness cases in three out of five study counties. The odds ratio of AC at lag 0 was 1.49 (95% confidence interval (CI): 1.04, 2.13) for Duval County and 1.35 (95% CI: 1.08, 1.68) for Hillsborough County. The search term drink had relatively low odds ratios: Leon County (1.03, 95% CI: 1.00, 1.06) and Miami-Dade County (1.05, 95% CI: 1.01, 1.10). Similarly, park also exhibited low odds ratios: Leon County (1.06, 95% CI: 1.00, 1.12) and Hillsborough County (1.05, 95% CI: 1.00, 1.11). All of these results indicate that more Twitter tweets or Google search results could be linked to more heat-related patients. However, we also found that the direction of the associations was counterintuitive for heat exhaustion and heat stroke, particularly at longer temporal lags.

### Dehydration

Next, GLM with a negative binomial distribution examined dehydration’s relationship to the weekly cycle, maximum temperature, and web data. Dehydration hospitalizations but not ED exhibited a strong weekly cycle where weekends tended to have a lower number of cases than weekdays (Additional file 1: Tables S7, S8). Interestingly, adding maximum temperature in the model did not always translate into model improvements. Only Duval, Hillsborough, and Miami-Dade County showed small improvements. Relatedly, maximum temperature marginally to significantly (beta coefficients range from 0.01 to 0.39) increased the risk of ED and hospitalization.

Comparable to temperature, adding one web data term moderately improved model fit (Fig. 2). At lag 0, the following terms were significant for ED: AC, AC repair, heat, heat stroke, hot weather, park, pool, swim, and water. Relatedly, AC, beer, drink, heat exhaustion, and hot weather web data were systematically related to hospitalizations. Park and pool, in particular, improved ED models in three out of five counties. For hospitalization cases, only the heat exhaustion search term improved fit in two out of five counties. AICs decreased up to 9.95 for ED and 6.13 for hospitalization. At lags of 1–3 days, all keywords from ED and hospitalization showed improvement. The search terms heat exhaustion, swim, and water improved ED while AC and heat exhaustion improved hospitalization models in three out of five counties. AICs decreased up to 14.93 for ED and 7.25 for hospitalization. More details can be found from Additional file 1: Tables S9 and S10.

Most of the web data were positively associated with dehydration ED cases (Fig. 3). Dehydration was positively correlated with heat, AC, AC repair, heat stroke, park, pool, swim, and water. Distinctly, park, pool, swim, and water tended to show a consistent positive relationship from lag 0 to lag 3. The beta coefficients for park and pool ranged from 0.00 to 0.01 in three counties at lag 0. AC tweets exhibited higher beta coefficients ranging from 0.02 to 0.04 in two counties at lag 0. Thus, a ten unit change in search volume increases the number of dehydration patients by up to 0.4.

### Renal illness

Negative binomial regression also investigated the relationship between renal illness, the weekly cycle, maximum temperature, and web data. Similar to dehydration, renal illness exhibited a weak ED but strong hospitalization weekly cycle (Additional file 1: Tables S11, S12). More patients sought health care during weekdays than weekends. Compared to heat-related illness, maximum temperature was inconsistently associated with the number of renal illnesses. Maximum temperature only significantly increased renal illness cases in Miami-Dade County for ED (beta coefficient: 0.13~0.17) and in Duval County for hospitalization (beta coefficient: 0.18~0.26).

Including a web search term slightly improved the model fit for both ED and hospitalizations (Fig. 2). At lag 0, keywords such as AC, AC repair, and pool improved ED models in one county. Drink, heat stroke, and swim were related to renal ED visits in two counties. Correspondingly, beer, drink, heat exhaustion, and pool improved one county’s hospitalization models and heat, park, and water improved hospitalization models in two counties. AICs decreased up to 5.89 for ED and 8.57 for hospitalization. At lags of 1 to 3 days, AC, drink, heat exhaustion, heat stroke, hot weather, park, pool, swim, tired, and water were significantly related to ED visits. Similarly, lagged AC, beer, heat, heat exhaustion, heat stroke, park, pool, swim, and water terms were related to hospitalizations. AICs decreased up to 7.07 for ED and 7.52 for hospitalization. Unlike other illnesses, we found inconsistent and place-specific associations from heat stroke, park, pool, and tired. In addition, web data except for heat and AC showed negative association with health outcomes. These results suggest the people at risk may be not well captured by Twitter or Google search. More details can be found from Additional file 1: Tables S13 and S14.

### ARMA-GARCH

#### Cardiovascular illness

**Fig. 4:**
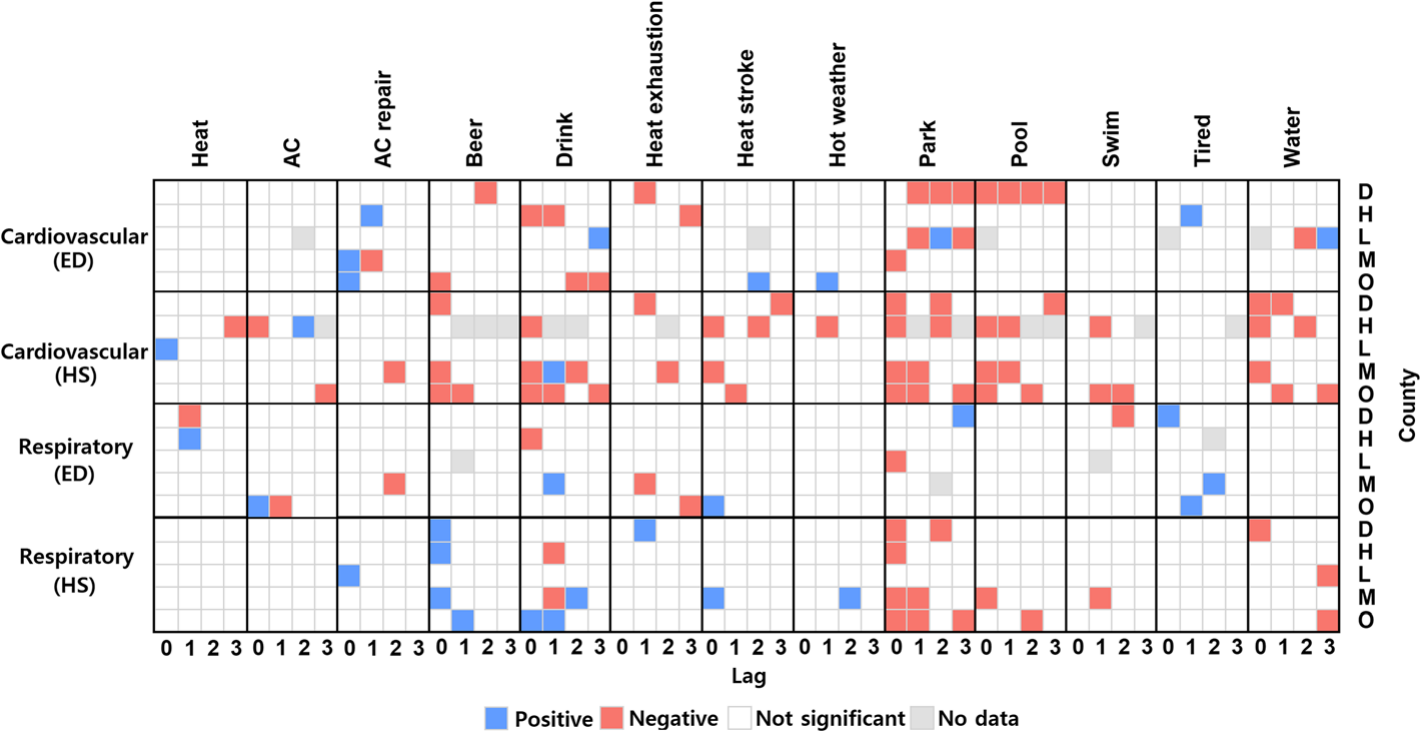
The significant beta coefficients of all web data up to 3 lags

We used ARMA-GARCH models to investigate the relationship between cardiovascular illness and web data after controlling for temporal autocorrelation. Adding the weekly cycle improved model performance based on mean absolute error and AIC (Additional file 1: Tables S15, S16). Not surprisingly, more patients sought healthcare on Mondays and other weekdays compared to weekends. Adding maximum temperature improved models for ED cases in Hillsborough and Miami-Dade Counties and hospitalization cases in Duval, Hillsborough, and Leon County. The significant temperature beta coefficients fell between 0.90 and 2.05 for ED. Somewhat surprisingly, Leon and Duval County’s ED visits were negatively associated with hospitalizations which contradicts the study hypothesis. The results suggest ED visits are more sensitive than hospitalizations to temperature increases. Figure 4 summarizes the direction of the ARMA-GARCH model beta coefficients. We found beer, drink, heat exhaustion, park, pool, and water tend to consistently have a negative relationship with cardiovascular illness cases in three out of five counties. Other keywords tended to show relatively inconsistent associations. Adding web data only slightly improved some ED and hospitalization models (Additional file 1: Figure S3, Tables S17, S18).

#### Respiratory illness

In the case of respiratory illness, we observed strong weekly cycles where weekdays had more cases than weekends (Additional file 1: Tables S19, S20). Including maximum temperature did not improve ED but slightly improved hospitalization models in Hillsborough and Orange County. Based on the significant negative beta coefficients, fewer patients were hospitalized when the temperature rose. Compared to other illnesses, respiratory illnesses showed more inconsistent associations between web data and health outcomes. The terms park and beer respectively exhibited the strongest negative and positive relationships with respiratory illnesses cases. While web search terms were significantly related to cases, the strength of the association did not improve either ED or hospitalization models (Additional file 1: Figure S3, Tables S21, S22).

## Discussion

The study examined the relationship between heat sensitive health outcomes and web data including Twitter and Google search volumes over time. We hypothesized that more frequent Twitter tweets or Google search results mentioning heat-related keywords are positively associated with health outcomes. The results partially supported our hypothesis. We found that heat-related illness and dehydration cases are positively associated with the concurrent day’s keywords such as heat, AC, park, pool, swim, and water. Other keywords were also positively associated with health outcomes when lagged effects were considered. The best fitting temporal lag varied by keyword. For example, the concurrent day’s AC and heat keywords from Twitter showed the strongest association with health outcomes. This finding aligns with how people use Twitter to express their feelings and thoughts in real time. On the other hand, Google search tended to show lagged effects between search terms and health outcomes. Specifically, park, pool, swim, and water search volumes increased before heat-related illness and dehydration cases. This finding might reflect behavioral patterns. For example, when temperature is forecast to rise, people may start making plans to visit cool places. For cardiovascular and renal disease, we were not able to find any strong association with web data. This is likely impacted by the age of patients suffering these illnesses. Because these illnesses are more common to advanced age, they might not Twitter and Google users.

Our study builds upon Li et al. [32] which examines Baidu web searches and heat stroke in Shanghai, China. Our paper investigates a wider range of heat sensitive outcomes, considers both social media and search engine data, and uses statistical analysis that controls for residual temporal autocorrelation. Our study shows that Twitter has slightly stronger and consistent associations with health outcomes than Google search. The Shanghai study found that the incidence of heat stroke exhibited a stronger correlation with heat stroke Internet searches than maximum temperature. Our study found the opposite where temperature accounted for more variability. This may be related to different climate conditions. Florida experiences higher and more extreme temperature events than Shanghai. These harsh climate environments could possibly give higher impacts on human health compared to Shanghai. The different media environment in China may also factor into this result. China’s state-run media may more effectively spread heat stroke prevention messages compared to other countries. The present study also found a consistent relationship between searching for water and dehydration cases. We speculate that migrant or minorities households may use search engines to look for filtered or bottled water [56]. The results suggest web data could enhance existing heat-related and dehydration surveillance systems.

This paper also suggests that activity patterns for reducing heat stress are associated with several health outcomes. More heat-related illness and dehydration patients were found when more people searched for park, pool and swim. This result is well aligned with Lane et al. [57]‘s qualitative heat study. They reported that about 51% of people went to cool places such as public place, place of business, and other places during very hot weather. On the other hand, Hayden [58] reported that people are not going to the park when temperature is too high in Phoenix, Arizona. We think this opposite behavioral pattern could stem from regional differences in climate and heat coping strategies. Notably, the results suggest web data can be as useful as maximum temperature for anticipating health outcomes that are exacerbated by heat. However, somewhat unexpectedly, park and pool keywords were negatively related with the number of cardiovascular and respiratory patients. It is possible that these terms are more commonly paired with non-heat related activities such as parking space or pool games.

We also investigated the relationship between maximum temperature and five different types of health outcomes. Overall, maximum temperature provided the strongest and most consistent relationships with the number of heat-related illness cases. This result aligns with previously published papers. Basu et al. [59] reported high ED heat-related illness risk (393.3%) during high temperature days. Ostro et al. [18] similarly showed an increase in heat stroke hospitalizations (166%) per 10 °F change in maximum temperature. The other four disease categories, however, showed a less consistent association with maximum temperature. For dehydration, Duval, Hillsborough, and Miami-Dade County showed positive relationship with maximum temperature whereas Leon and Orange County did not show any significant relationships. We also found a positive relationship between renal illness cases and maximum temperature in Duval and Miami-Dade County. On the other hand, Hillsborough, Leon, and Orange County did not show any significant temperature associations. We suspect the relationships reflected demographic characteristics of each county. Some types of illnesses such as renal and cardiovascular illness are more common with advanced age. They tend to be more sensitive to high temperature due to degraded organ functions and high prevalence of chronic and degenerative diseases. Relatively younger median ages in Leon and Orange County could be one reason for insignificant association with maximum temperature. In addition, our study’s ED/hospitalization dataset does not capture heat-related illness from tourists whose billing address is outside of the state. Thus, heat-related illness may be underreported from Orange County which hosts many theme parks frequented by domestic and international tourists.

This paper contains several key limitations. First, the Internet and Twitter users may not be representative of the U.S. population. Perrin and Duggan [60] described that only 58% of American adults over 65 years old use the Internet. They also pointed out important racial differences. They found that smaller percentages of African-Americans adults (78%) and Hispanics (81%) use the Internet than whites (85%) and English-speaking Asian Americans (97%). In addition, the level of education and average income are related to the internet use. Ünver [61] showed people with more education and higher average income are more likely to use internet than those with less education and lower average income. Web data could underestimate the cases for less educated and low income people who generally lie at high heat-related risk. Twitter users may be even less representative of the general population. Greenwood et al. [62] reported that while more than one third of Internet users between 18 and 29 years old use Twitter, only 10 % of Internet users over 65 years old use Twitter. Duggan and Brenner [63] also shows that non-Caucasian people uses Twitter more commonly. Furthermore, we only used English language Tweets and Google search terms, which could possible miss or underestimate Hispanic people. These demographic discrepancies add additional uncertainty and errors to the web data. Therefore, this technique is not a perfect stand-alone tool for heat surveillance but may be used in conjunction with other surveillance tools.

This study uses ICD billing codes to classify heat-related health outcomes. Several papers point out that the billing codes contain multiple errors [64, 65]. For example, O’Malley et al. [66] enumerated steps that may affect coding accuracy: 1) the communication between the patient and his/her admitting clerk or physician, 2) clinician’s knowledge about the best diagnostic tests and procedures, 3) clinicians’ delay in gaining new medical advances or diagnostic tools, 4) the use of synonyms and abbreviations, 5) physicians’ or other staffs’ mistakes including omissions and transcription (from voice to record), 6) physician’s effort to check the accuracy of the codes, 7) coders’ experience, attentions, and persistence, and 8) upcoding (selecting codes of high reimbursement value). These potential errors could also introduce additional uncertainty and error into the analysis.

Furthermore, the discrepancy between the health and web search geographic boundaries may weaken the strength of observed associations. To recap, the Google search data are aggregated to commercial direct marketing area boundaries which include several counties. In contrast, the heat-related health outcomes are reported at the county level. The modifiable areal unit problem, called MAUP, could be one major error when aggregating data into a certain type of spatial partition. This problem is well studied by many researchers [67, 68].

In spite of these weaknesses, public health researchers will continue to explore the utility of web search data due to its low maintenance/operating cost and promptness in detecting illnesses. Social media and web search data could be most helpful for jurisdictions without real time surveillance or an extreme heat early warning system. We understand that a small number of governments can operate web data-based surveillance system due to limited resources, employees, and familiarity with technology. Perhaps the federal government or a consortium of governments, academics, and non-profits may pool their resources to disseminate this information. We hope this research will stimulate other researchers to improve current surveillance systems.

## Additional file

Additional file 1: **Figure S1.** Google commercial boundary. Research areas are in dark grey. **Figure S2.** Pearson correlation coefficients between maximum temperature and web data. We only colored significant Pearson’s correlation coefficients. Maximum temperatures up to three lag days were considered. **Figure S3.** AIC changes after adding one of web data to the second model (days of week and maximum temperature). Minus (blue) means model improvement. **Table S1.** Demographic estimate summary of each county in 2014 from the U.S. Census Bureau’s American Community Survey. **Table S2.** Descriptive summary on weather conditions and AICs. **Table S3.** Heat-related-illness ED model specifications (*** < 0.05, ** 0.05 ~ 0.10, * 0.10 ~ 0.15). **Table S4.** Heat-related-illness hospitalization model specifications (*** < 0.05, ** 0.05 ~ 0.10, * 0.10 ~ 0.15). **Table S5.** Heat-related-illness ED model specifications up to three lag days (*** < 0.05, ** 0.05 ~ 0.10, * 0.10 ~ 0.15). **Table S6.** Heat-related-illness hospitalization model specifications up to three lag days (*** < 0.05, ** 0.05 ~ 0.10, * 0.10 ~ 0.15). **Table S7.** Dehydration ED model specifications (*** < 0.05, ** 0.05 ~ 0.10, * 0.10 ~ 0.15). **Table S8.** Dehydration hospitalization model specifications (*** < 0.05, ** 0.05 ~ 0.10, * 0.10 ~ 0.15). **Table S9.** Dehydration ED model specifications up to three lag days (*** < 0.05, ** 0.05 ~ 0.10, * 0.10 ~ 0.15). **Table S10.** Dehydration hospitalization model specifications up to three lag days (*** < 0.05, ** 0.05 ~ 0.10, * 0.10 ~ 0.15). **Table S11.** Renal illness ED model specifications (*** < 0.05, ** 0.05 ~ 0.10, * 0.10 ~ 0.15). **Table S12.** Renal illness hospitalization model specifications (*** < 0.05, ** 0.05 ~ 0.10, * 0.10 ~ 0.15). **Table S13.** Renal illness ED model specifications up to three lag days (*** < 0.05, ** 0.05 ~ 0.10, * 0.10 ~ 0.15). **Table S14.** Renal illness hospitalization model specifications up to three lag days (*** < 0.05, ** 0.05 ~ 0.10, * 0.10 ~ 0.15). **Table S15.** Cardiovascular illness ED model specifications (*** < 0.05, ** 0.05 ~ 0.10, * 0.10 ~ 0.15). **Table S16.** Cardiovascular illness hospitalization model specifications (*** < 0.05, ** 0.05 ~ 0.10, * 0.10 ~ 0.15). **Table S17.** Cardiovascular illness ED model specifications up to three lag days (*** < 0.05, ** 0.05 ~ 0.10, * 0.10 ~ 0.15). **Table S18.** Cardiovascular illness hospitalization model specifications up to three lag days (*** < 0.05, ** 0.05 ~ 0.10, * 0.10 ~ 0.15). **Table S19.** Respiratory illness ED model specifications (*** < 0.05, ** 0.05 ~ 0.10, * 0.10 ~ 0.15). **Table S20.** Respiratory illness hospitalization model specifications (*** < 0.05, ** 0.05 ~ 0.10, * 0.10 ~ 0.15). **Table S21.** Respiratory illness ED model specifications up to three lag dayss (*** < 0.05, ** 0.05 ~ 0.10, * 0.10 ~ 0.15). **Table S22.** Respiratory illness hospitalization model specifications up to three lag days (*** < 0.05, ** 0.05 ~ 0.10, * 0.10 ~ 0.15). (DOCX 1020 kb)

## Data Availability

The weather and social media datasets used in this study are available from the corresponding author upon request. Based on our data use agreement, we cannot share the health outcomes dataset. The data can be requested from Florida’s Agency for Health Care Administration.
