# Supplementary material for: Using web data to improve surveillance for heat sensitive health outcomes
Source: Environ Health. 2019 Jul 9;18:59. doi: 10.1186/s12940-019-0499-x (PMC6615306; doi:10.1186/s12940-019-0499-x)
Supplement: Supplementary file 1 — Figure S1. Google commercial boundary. Research areas are in dark grey. Figure S2. Pearson correlation coefficients between maximum temperature and web data. We only colored significant Pearson’s correlation coefficients. Maximum temperatures up to three lag days were considered. Figure S3. AIC changes after adding one of web data to the second model (days of week and maximum temperature). Minus (blue) means model improvement. Table S1. Demographic estimate summary of each county in 2014 from the U.S. Census Bureau’s American Community Survey. Table S2. Descriptive summary on weather conditions and AICs. Table S3. Heat-related-illness ED model specifications (*** < 0.05, ** 0.05 ~ 0.10, * 0.10 ~ 0.15). Table S4. Heat-related-illness hospitalization model specifications (*** < 0.05, ** 0.05 ~ 0.10, * 0.10 ~ 0.15). Table S5. Heat-related-illness ED model specifications up to three lag days (*** < 0.05, ** 0.05 ~ 0.10, * 0.10 ~ 0.15). Table S6. Heat-related-illness hospitalization model specifications up to three lag days (*** < 0.05, ** 0.05 ~ 0.10, * 0.10 ~ 0.15). Table S7. Dehydration ED model specifications (*** < 0.05, ** 0.05 ~ 0.10, * 0.10 ~ 0.15). Table S8. Dehydration hospitalization model specifications (*** < 0.05, ** 0.05 ~ 0.10, * 0.10 ~ 0.15). Table S9. Dehydration ED model specifications up to three lag days (*** < 0.05, ** 0.05 ~ 0.10, * 0.10 ~ 0.15). Table S10. Dehydration hospitalization model specifications up to three lag days (*** < 0.05, ** 0.05 ~ 0.10, * 0.10 ~ 0.15). Table S11. Renal illness ED model specifications (*** < 0.05, ** 0.05 ~ 0.10, * 0.10 ~ 0.15). Table S12. Renal illness hospitalization model specifications (*** < 0.05, ** 0.05 ~ 0.10, * 0.10 ~ 0.15). Table S13. Renal illness ED model specifications up to three lag days (*** < 0.05, ** 0.05 ~ 0.10, * 0.10 ~ 0.15). Table S14. Renal illness hospitalization model specifications up to three lag days (*** < 0.05, ** 0.05 ~ 0.10, * 0.10 ~ 0.15). Table S15. Cardiovascular illness ED model [file 12940_2019_499_MOESM1_ESM.docx]

Additional file 1


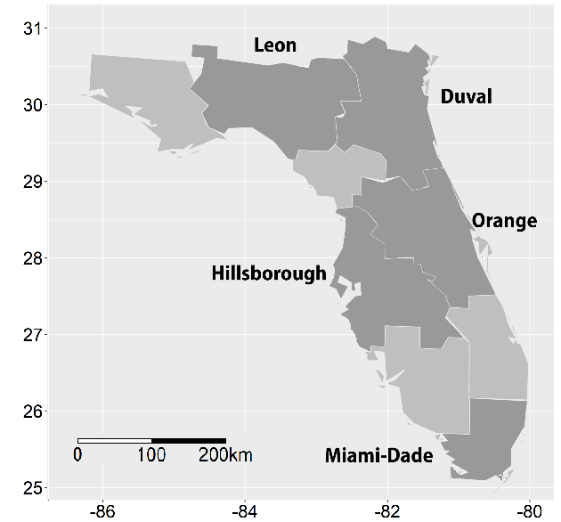


Figure S1. Google commercial boundary. Research areas are in dark grey.


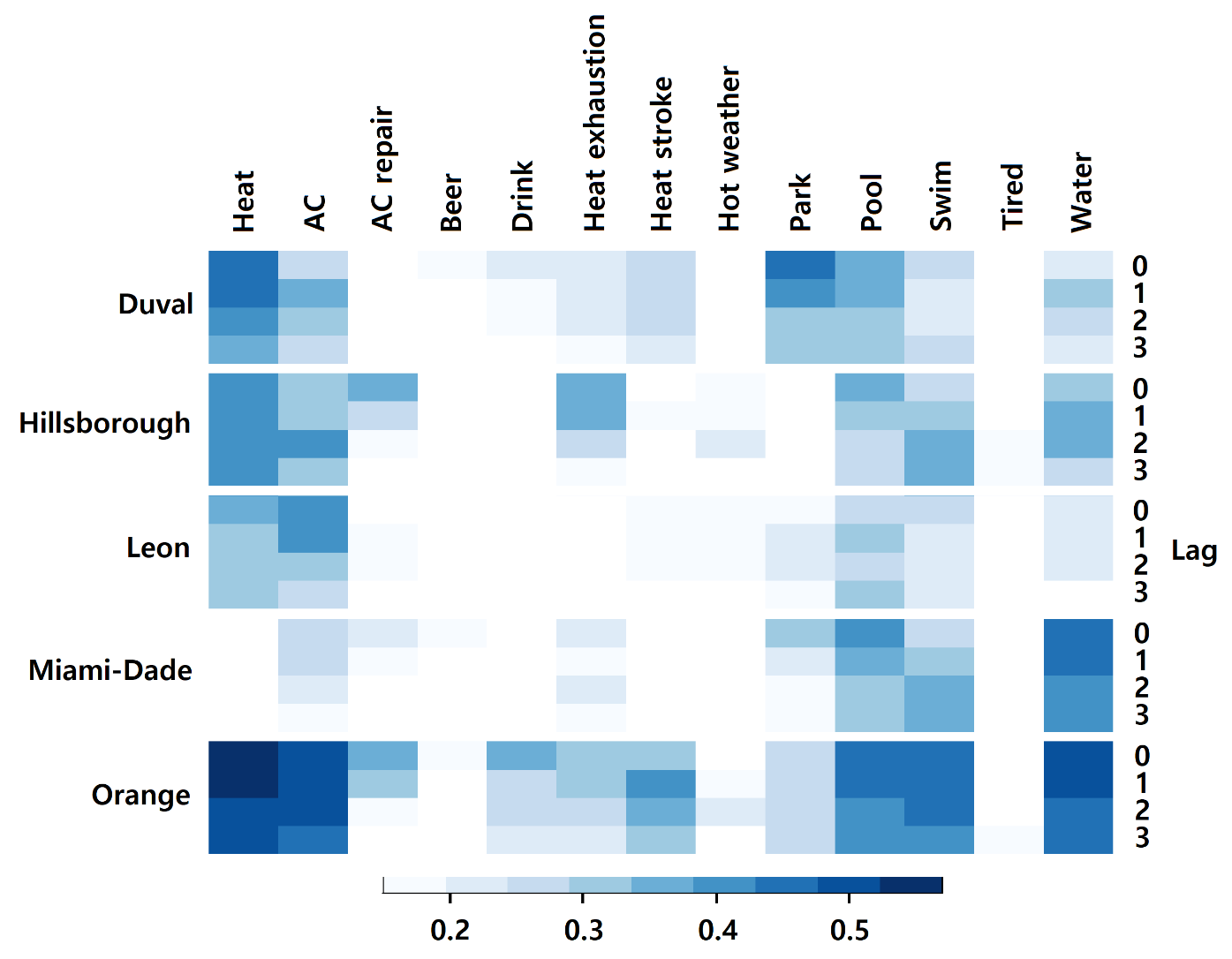


Figure S2. Pearson correlation coefficients between maximum temperature and web data. We only colored significant Pearson’s correlation coefficients. Maximum temperatures up to three lag days were considered.


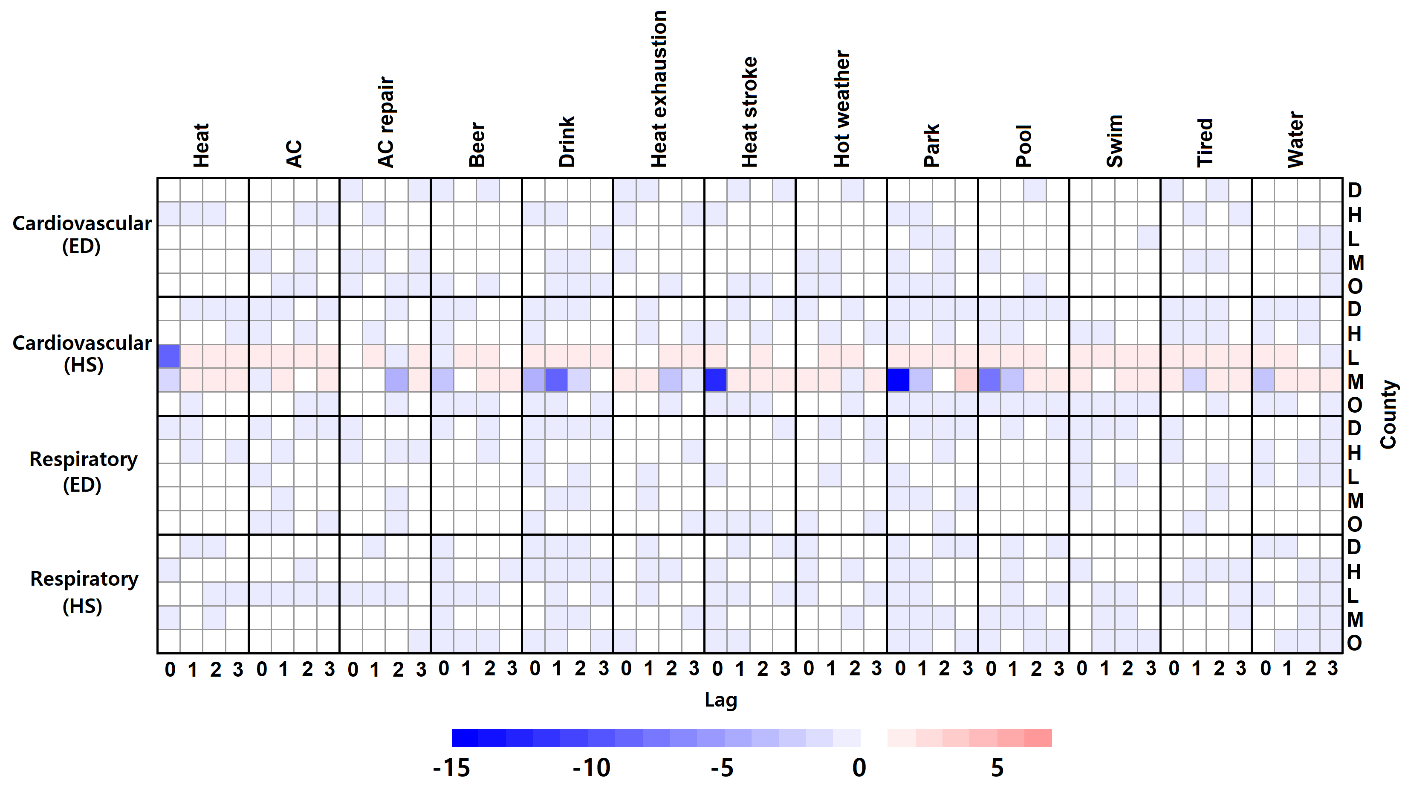


Figure S3. AIC changes after adding one of web data to the second model (days of week and maximum temperature). Minus (blue) means model improvement.

Table S1. Demographic estimate summary of each county in 2014 from the U.S. Census Bureau’s American Community Survey

| Variable | sub-variable | Duval | Hillsborough | Leon | Miami-Dade | Orange |
| --- | --- | --- | --- | --- | --- | --- |
| Population | Total (numbers) | 898,372 | 1,317,116 | 284,053 | 2,667,299 | 1,253,631 |
| Age | Median (years) | 36.1 | 36.7 | 30.3 | 39.4 | 34.5 |
|  | 5 < (%) | 6.8 | 6.4 | 5.2 | 5.8 | 6.3 |
|  | 65 > (%) | 12.7 | 13.1 | 11.4 | 15.2 | 10.8 |
|  | Male (%) | 48.4 | 48.7 | 47.6 | 48.5 | 49.2 |
|  | Female (%) | 51.6 | 51.3 | 52.4 | 51.5 | 50.8 |
| Race | White (%) | 61.8 | 75.4 | 62.5 | 78.0 | 68.7 |
|  | Black (%) | 30.2 | 17.5 | 31.5 | 18.9 | 22.5 |
|  | Others (%) | 8.0 | 7.2 | 5.9 | 3.1 | 8.8 |
| Ethnicity | Non-Hispanic (%) | 91.4 | 73.4 | 94.0 | 33.4 | 70.7 |
|  | Hispanic (%) | 8.6 | 26.6 | 6.0 | 66.6 | 29.3 |

Table S2. Descriptive summary on weather conditions and AICs

|  | Temperature (℉) | | | Discomfort index | | |
| --- | --- | --- | --- | --- | --- | --- |
|  | Maximum | Mean | Minimum | Maximum | Mean | Minimum |
| Duval | 90.0 | 78.4 | 69.6 | 27.3 | 24.0 | 20.6 |
| Hillsborough | 89.2 | 79.7 | 72.2 | 27.2 | 24.5 | 21.9 |
| Leon | 91.6 | 77.8 | 66.3 | 27.3 | 23.5 | 18.9 |
| Miami-Dade | 89.5 | 81.3 | 74.5 | 27.7 | 25.4 | 23.1 |
| Orange | 90.6 | 78.6 | 70.0 | 27.7 | 24.3 | 21.1 |
| AICs | 966 | 966 | 968 | 967 | 967 | 970 |

Table S3. Heat-related-illness ED model specifications (*** <0.05, ** 0.05 ~ 0.10, * 0.10 ~ 0.15)

| County | Keywords | | Wed | Thu | Fri | Sat | Sun | Mon | Max T | keyword | AUC | AIC |
| --- | --- | --- | --- | --- | --- | --- | --- | --- | --- | --- | --- | --- |
| Duval | None | | 0.25 | 0.74 | -0.25 | 0.42 | 0.25 | -0.09 | - | - | 0.58 | 237.44 |
|  |  |  | 0.04 | 0.64 | -0.78 | 0.24 | 0.22 | -0.15 | 0.27*** | - | 0.81 | 189.29 |
|  | Twitter | Heat | 0.01 | 0.74 | -0.77 | 0.29 | 0.31 | -0.15 | 0.23*** | 0.14** | 0.82 | 188.43 |
|  |  | AC | -0.09 | 0.75 | -0.99 | 0.25 | 0.19 | -0.03 | 0.25*** | 0.40*** | 0.82 | 185.75 |
|  | Google Search | AC repair | 0.03 | 0.63 | -0.81 | 0.22 | 0.21 | -0.17 | 0.27*** | 0.00 | 0.81 | 191.25 |
|  |  | Beer | 0.09 | 0.65 | -0.66 | 0.65 | 0.60 | -0.15 | 0.28*** | -0.02** | 0.81 | 188.16 |
|  |  | Drink | 0.03 | 0.66 | -0.79 | 0.35 | 0.33 | -0.17 | 0.28*** | -0.01 | 0.81 | 190.65 |
|  |  | Heat exhaustion | 0.02 | 0.64 | -0.78 | 0.22 | 0.21 | -0.15 | 0.27*** | 0.00 | 0.81 | 191.19 |
|  |  | Heat stroke | 0.13 | 0.64 | -0.80 | 0.37 | 0.20 | -0.21 | 0.25*** | 0.02 | 0.82 | 189.31 |
|  |  | Hot weather | 0.02 | 0.64 | -0.78 | 0.32 | 0.27 | -0.11 | 0.27*** | -0.01 | 0.81 | 189.96 |
|  |  | Park | 0.04 | 0.67 | -0.89 | 0.06 | 0.17 | -0.19 | 0.25*** | 0.02 | 0.81 | 190.47 |
|  |  | Pool | 0.10 | 0.59 | -0.86 | 0.04 | 0.00 | -0.20 | 0.24*** | 0.02** | 0.82 | 188.07 |
|  |  | Swim | 0.17 | 0.69 | -0.53 | 0.21 | 0.16 | -0.09 | 0.25*** | 0.02*** | 0.83 | 186.67 |
|  |  | Tired | -0.24 | 0.41 | -0.90 | -0.01 | -0.06 | -0.20 | 0.28*** | 0.03*** | 0.82 | 184.50 |
|  |  | water | 0.04 | 0.65 | -0.78 | 0.25 | 0.23 | -0.14 | 0.27*** | 0.00 | 0.81 | 191.27 |
| Hillsborough | None | | 0.07 | 0.00 | 0.07 | 0.07 | -0.11 | -0.27 | - | - | 0.53 | 236.55 |
|  |  |  | -0.11 | -0.23 | 0.16 | 0.03 | -0.16 | -0.33 | 0.37*** | - | 0.79 | 191.07 |
|  | Twitter | Heat | -0.17 | -0.27 | 0.03 | 0.00 | -0.13 | -0.34 | 0.34*** | 0.07 | 0.80 | 191.52 |
|  |  | AC | -0.24 | -0.26 | 0.08 | 0.10 | -0.11 | -0.53 | 0.33*** | 0.30*** | 0.82 | 184.90 |
|  | Google Search | AC repair | -0.16 | -0.33 | 0.04 | -0.29 | -0.18 | -0.44 | 0.34*** | 0.02** | 0.80 | 190.16 |
|  |  | Beer | -0.12 | -0.38 | -0.05 | -0.42 | -0.46 | -0.42 | 0.36*** | 0.02 | 0.79 | 192.17 |
|  |  | Drink | -0.09 | -0.23 | 0.16 | -0.01 | -0.19 | -0.30 | 0.37*** | 0.00 | 0.79 | 193.03 |
|  |  | Heat exhaustion | -0.11 | -0.30 | 0.16 | -0.01 | -0.08 | -0.29 | 0.40*** | -0.01 | 0.80 | 192.21 |
|  |  | Heat stroke | -0.13 | -0.38 | 0.13 | -0.09 | -0.31 | -0.52 | 0.39*** | -0.01** | 0.80 | 190.06 |
|  |  | Hot weather | -0.03 | -0.33 | 0.18 | 0.00 | -0.25 | -0.49 | 0.42*** | -0.02** | 0.81 | 189.41 |
|  |  | Park | -0.11 | -0.23 | 0.14 | -0.01 | -0.18 | -0.33 | 0.37*** | 0.00 | 0.79 | 193.06 |
|  |  | Pool | -0.11 | -0.24 | 0.15 | -0.01 | -0.19 | -0.35 | 0.37*** | 0.00 | 0.79 | 193.04 |
|  |  | Swim | -0.12 | -0.21 | 0.15 | -0.03 | -0.19 | -0.37 | 0.36*** | 0.01 | 0.79 | 192.71 |
|  |  | Tired | -0.10 | -0.20 | 0.22 | 0.02 | -0.23 | -0.41 | 0.37*** | 0.01 | 0.80 | 191.06 |
|  |  | water | -0.09 | -0.25 | 0.16 | 0.05 | -0.19 | -0.37 | 0.36*** | 0.02 | 0.80 | 192.47 |
| Leon | None | | -0.90 | 0.00 | -0.57 | -0.29 | -0.90 | -0.57 | - | - | 0.60 | 170.79 |
|  |  |  | -1.10 | -0.18 | -0.94 | -0.70 | -1.29* | -0.85 | 0.22*** | - | 0.76 | 151.76 |
|  | Twitter | Heat | -1.10 | -0.17 | -0.94 | -0.70 | -1.29* | -0.83 | 0.23*** | -0.01 | 0.76 | 153.75 |
|  |  | AC | -1.15 | -0.17 | -0.96 | -0.70 | -1.20 | -0.81 | 0.21*** | 0.12 | 0.76 | 153.36 |
|  | Google Search | AC repair | -1.11 | -0.26 | -0.93 | -0.77 | -1.30* | -0.90 | 0.23*** | 0.01 | 0.76 | 153.21 |
|  |  | Beer | -1.15 | 0.08 | -0.76 | -0.38 | -1.21* | -0.97 | 0.25*** | -0.02** | 0.79 | 150.45 |
|  |  | Drink | -1.17 | -0.11 | -1.12 | -1.15 | -1.83*** | -0.86 | 0.23*** | 0.03*** | 0.78 | 150.03 |
|  |  | Heat exhaustion | -0.97 | -0.19 | -0.97 | -0.73 | -1.31* | -0.57 | 0.24*** | -0.27 | 0.78 | 150.58 |
|  |  | Heat stroke | -1.10 | -0.16 | -0.95 | -0.69 | -1.28* | -0.85 | 0.22*** | 0.00 | 0.76 | 153.74 |
|  |  | Hot weather | -1.15 | -0.24 | -1.03 | -0.73 | -1.25* | -0.86 | 0.21*** | 0.01 | 0.76 | 153.19 |
|  |  | Park | -1.10 | -0.18 | -0.95 | -0.70 | -1.28* | -0.85 | 0.22*** | 0.00 | 0.76 | 153.74 |
|  |  | Pool | -1.10 | -0.17 | -0.93 | -0.73 | -1.33* | -0.85 | 0.22*** | 0.00 | 0.76 | 153.68 |
|  |  | Swim | -1.06 | -0.09 | -0.86 | -0.61 | -1.24* | -0.78 | 0.23*** | -0.01 | 0.78 | 152.57 |
|  |  | Tired | -1.11 | -0.13 | -0.87 | -0.70 | -1.33* | -0.82 | 0.23*** | 0.01 | 0.77 | 153.16 |
|  |  | water | -1.12 | -0.22 | -0.99 | -0.73 | -1.29* | -0.85 | 0.23*** | -0.01 | 0.76 | 153.50 |
| Miami-Dade | None | | -0.78 | -0.53 | 0.61 | 1.01* | -0.60 | 0.25 | - | - | 0.67 | 229.08 |
|  |  |  | -0.85 | -0.64 | 0.57 | 0.90 | -0.68 | 0.35 | 0.25*** | - | 0.75 | 213.27 |
|  | Twitter | Heat | -0.84 | -0.64 | 0.59 | 0.90 | -0.68 | 0.36 | 0.25*** | 0.00 | 0.75 | 215.11 |
|  |  | AC | -0.86 | -0.59 | 0.56 | 0.97* | -0.62 | 0.33 | 0.23*** | 0.04 | 0.75 | 214.55 |
|  | Google Search | AC repair | -0.84 | -0.63 | 0.58 | 0.94 | -0.61 | 0.35 | 0.24*** | 0.01 | 0.75 | 214.87 |
|  |  | Beer | -0.86 | -0.65 | 0.48 | 0.74 | -0.84 | 0.33 | 0.24*** | 0.01 | 0.75 | 214.95 |
|  |  | Drink | -0.91 | -0.70 | 0.47 | 0.73 | -0.85 | 0.31 | 0.25*** | 0.01 | 0.75 | 214.86 |
|  |  | Heat exhaustion | -0.84 | -0.63 | 0.56 | 0.90 | -0.66 | 0.35 | 0.25*** | 0.00 | 0.76 | 215.00 |
|  |  | Heat stroke | -0.84 | -0.64 | 0.57 | 0.91 | -0.69 | 0.34 | 0.25*** | 0.00 | 0.75 | 215.24 |
|  |  | Hot weather | -0.85 | -0.64 | 0.57 | 0.90 | -0.68 | 0.35 | 0.25*** | 0.00 | 0.75 | 215.27 |
|  |  | Park | -0.86 | -0.68 | 0.44 | 0.64 | -0.90 | 0.25 | 0.23*** | 0.02 | 0.75 | 214.53 |
|  |  | Pool | -0.82 | -0.64 | 0.56 | 0.84 | -0.82 | 0.30 | 0.23*** | 0.01 | 0.75 | 214.72 |
|  |  | Swim | -0.86 | -0.64 | 0.60 | 0.91 | -0.68 | 0.32 | 0.23*** | 0.01 | 0.75 | 214.68 |
|  |  | Tired | -0.85 | -0.63 | 0.58 | 0.90 | -0.68 | 0.35 | 0.25*** | 0.00 | 0.75 | 215.25 |
|  |  | water | -0.88 | -0.63 | 0.52 | 0.88 | -0.82 | 0.24 | 0.21*** | 0.03 | 0.75 | 213.82 |
| Orange | None | | -0.26 | 0.56 | 0.07 | -0.43 | -0.43 | -0.43 | - | - | 0.59 | 238.94 |
|  |  |  | -0.54 | 0.36 | -0.21 | -0.72 | -0.65 | -0.64 | 0.25*** | - | 0.76 | 211.55 |
|  | Twitter | Heat | -0.54 | 0.43 | -0.19 | -0.71 | -0.61 | -0.62 | 0.22*** | 0.03 | 0.76 | 212.86 |
|  |  | AC | -0.51 | 0.42 | -0.15 | -0.67 | -0.61 | -0.60 | 0.24*** | 0.03 | 0.76 | 213.39 |
|  | Google Search | AC repair | -0.55 | 0.38 | -0.24 | -0.74 | -0.68 | -0.65 | 0.25*** | 0.00 | 0.76 | 213.43 |
|  |  | Beer | -0.57 | 0.34 | -0.45 | -1.45** | -1.31** | -0.61 | 0.23*** | 0.03* | 0.76 | 211.29 |
|  |  | Drink | -0.62 | 0.28 | -0.34 | -1.14* | -0.94 | -0.66 | 0.23*** | 0.02 | 0.76 | 212.40 |
|  |  | Heat exhaustion | -0.53 | 0.45 | -0.10 | -0.67 | -0.61 | -0.52 | 0.23*** | 0.01* | 0.76 | 211.43 |
|  |  | Heat stroke | -0.62 | 0.25 | -0.19 | -0.72 | -0.77 | -0.67 | 0.22*** | 0.02** | 0.76 | 210.42 |
|  |  | Hot weather | -0.55 | 0.38 | -0.20 | -0.75 | -0.65 | -0.65 | 0.25*** | 0.00 | 0.76 | 213.32 |
|  |  | Park | -0.54 | 0.36 | -0.23 | -0.78 | -0.67 | -0.65 | 0.24*** | 0.00 | 0.76 | 213.51 |
|  |  | Pool | -0.54 | 0.35 | -0.22 | -0.83 | -0.76 | -0.68 | 0.23*** | 0.01 | 0.76 | 213.19 |
|  |  | Swim | -0.55 | 0.35 | -0.21 | -0.71 | -0.65 | -0.64 | 0.25*** | 0.00 | 0.76 | 213.50 |
|  |  | Tired | -0.52 | 0.30 | -0.34 | -0.89 | -0.72 | -0.77 | 0.26*** | -0.02** | 0.77 | 210.84 |
|  |  | water | -0.49 | 0.44 | -0.16 | -0.78 | -0.83 | -0.82 | 0.21*** | 0.04** | 0.76 | 210.87 |

Table S4. Heat-related-illness hospitalization model specifications (*** <0.05, ** 0.05 ~ 0.10, * 0.10 ~ 0.15)

| County | Keywords | | Wed | Thu | Fri | Sat | Sun | Mon | Max T | keyword | AUC | AIC |
| --- | --- | --- | --- | --- | --- | --- | --- | --- | --- | --- | --- | --- |
| Duval | None | | 0.56 | 1.07 | 0.56 | 1.20* | 0.29 | 0.29 | - | - | 0.61 | 183.01 |
|  |  |  | 0.39 | 0.83 | 0.06 | 0.92 | -0.04 | 0.25 | 0.30*** | - | 0.81 | 152.25 |
|  | Twitter | Heat | 0.55 | 1.06 | 0.27 | 1.15 | 0.28 | 0.28 | 0.23*** | 0.15** | 0.82 | 150.66 |
|  |  | AC | 0.32 | 0.86 | -0.11 | 0.85 | -0.16 | 0.30 | 0.29*** | 0.24* | 0.82 | 151.84 |
|  | Google Search | AC repair | 0.39 | 0.83 | 0.01 | 0.88 | -0.06 | 0.21 | 0.30*** | 0.00 | 0.81 | 154.16 |
|  |  | Beer | 0.30 | 0.63 | 0.13 | 1.27* | 0.26 | 0.23 | 0.33*** | -0.02 | 0.82 | 152.15 |
|  |  | Drink | 0.35 | 0.83 | 0.03 | 0.83 | -0.18 | 0.23 | 0.29*** | 0.01 | 0.82 | 153.86 |
|  |  | Heat exhaustion | 0.39 | 0.83 | 0.06 | 0.91 | -0.04 | 0.24 | 0.30*** | 0.00 | 0.81 | 154.24 |
|  |  | Heat stroke | 0.39 | 0.83 | 0.08 | 0.89 | -0.02 | 0.27 | 0.30*** | 0.00 | 0.81 | 154.16 |
|  |  | Hot weather | 0.35 | 0.75 | 0.07 | 1.03 | 0.03 | 0.27 | 0.32*** | -0.02** | 0.83 | 150.50 |
|  |  | Park | 0.35 | 0.81 | -0.16 | 0.68 | -0.14 | 0.14 | 0.29*** | 0.02 | 0.81 | 153.41 |
|  |  | Pool | 0.40 | 0.78 | 0.00 | 0.75 | -0.19 | 0.22 | 0.29*** | 0.01 | 0.81 | 153.66 |
|  |  | Swim | 0.46 | 0.85 | 0.27 | 0.95 | -0.10 | 0.27 | 0.29*** | 0.01 | 0.81 | 152.98 |
|  |  | Tired | 0.46 | 0.93 | 0.09 | 0.98 | 0.02 | 0.29 | 0.30*** | -0.01 | 0.81 | 154.02 |
|  |  | water | 0.44 | 0.89 | 0.09 | 0.97 | 0.03 | 0.29 | 0.31*** | -0.01 | 0.81 | 154.06 |
| Hillsborough | None | | 0.35 | -0.86 | 0.35 | 0.53 | 0.15 | -0.57 | - | - | 0.62 | 201.68 |
|  |  |  | 0.21 | -1.21* | 0.40 | 0.52 | 0.09 | -0.66 | 0.26*** | - | 0.76 | 182.21 |
|  | Twitter | Heat | 0.12 | -1.36* | 0.14 | 0.54 | 0.23 | -0.80 | 0.18*** | 0.17*** | 0.78 | 173.30 |
|  |  | AC | 0.17 | -1.24* | 0.45 | 0.71 | 0.26 | -0.68 | 0.23*** | 0.20*** | 0.77 | 179.99 |
|  | Google Search | AC repair | 0.22 | -1.18 | 0.42 | 0.58 | 0.11 | -0.65 | 0.27*** | 0.00 | 0.76 | 184.12 |
|  |  | Beer | 0.21 | -1.23* | 0.37 | 0.45 | 0.05 | -0.68 | 0.26*** | 0.00 | 0.76 | 184.18 |
|  |  | Drink | 0.32 | -1.22* | 0.41 | 0.25 | -0.11 | -0.53 | 0.26*** | 0.02 | 0.77 | 182.25 |
|  |  | Heat exhaustion | 0.10 | -1.48** | 0.39 | 0.46 | 0.12 | -0.59 | 0.31*** | -0.02** | 0.77 | 180.87 |
|  |  | Heat stroke | 0.21 | -1.07 | 0.38 | 0.70 | 0.20 | -0.44 | 0.26*** | 0.02*** | 0.77 | 179.68 |
|  |  | Hot weather | 0.21 | -1.22* | 0.40 | 0.51 | 0.09 | -0.68 | 0.26*** | 0.00 | 0.76 | 184.14 |
|  |  | Park | 0.27 | -1.15 | -0.01 | -0.22 | -0.37 | -0.78 | 0.26*** | 0.05*** | 0.77 | 180.08 |
|  |  | Pool | 0.25 | -1.23* | 0.37 | 0.21 | -0.20 | -0.83 | 0.25*** | 0.02 | 0.76 | 182.39 |
|  |  | Swim | 0.16 | -1.08 | 0.41 | 0.39 | 0.04 | -0.79 | 0.25*** | 0.02* | 0.76 | 181.91 |
|  |  | Tired | 0.21 | -1.20* | 0.41 | 0.51 | 0.08 | -0.67 | 0.26*** | 0.00 | 0.76 | 184.18 |
|  |  | water | 0.24 | -1.19* | 0.39 | 0.56 | 0.04 | -0.73 | 0.25*** | 0.03 | 0.76 | 182.93 |
| Leon | None | | 0.69 | -16.48 | -0.04 | -16.48 | 1.15 | 0.69 | - | - | 0.73 | 76.16 |
|  |  |  | 0.66 | -16.54 | -0.25 | -16.67 | 1.01 | 0.57 | 0.17** | - | 0.83 | 72.99 |
|  | Twitter | Heat | 0.62 | -16.58 | -0.29 | -16.68 | 1.03 | 0.47 | 0.16* | 0.05 | 0.83 | 74.81 |
|  |  | AC | 0.64 | -16.53 | -0.26 | -16.67 | 1.18 | 0.66 | 0.16* | 0.18 | 0.82 | 74.70 |
|  | Google Search | AC repair | 0.65 | -16.59 | -0.24 | -16.72 | 1.01 | 0.54 | 0.17** | 0.00 | 0.83 | 74.93 |
|  |  | Beer | 0.65 | -16.34 | -0.11 | -16.47 | 1.09 | 0.50 | 0.18*** | -0.02 | 0.84 | 74.51 |
|  |  | Drink | 0.63 | -17.46 | -0.40 | -18.11 | 0.55 | 0.59 | 0.18** | 0.03 | 0.84 | 73.61 |
|  |  | Heat exhaustion | 0.80 | -17.54 | -0.27 | -17.69 | 1.02 | 0.80 | 0.19*** | -0.30 | 0.84 | 73.43 |
|  |  | Heat stroke | 0.65 | -17.64 | -0.18 | -17.78 | 0.99 | 0.59 | 0.17** | 0.01 | 0.84 | 74.31 |
|  |  | Hot weather | 0.67 | -16.52 | -0.23 | -16.66 | 1.00 | 0.57 | 0.17** | 0.00 | 0.83 | 74.98 |
|  |  | Park | 0.62 | -17.78 | -0.89 | -17.96 | 1.07 | 0.43 | 0.15** | 0.06*** | 0.88 | 71.27 |
|  |  | Pool | 0.68 | -16.48 | -0.19 | -16.84 | 0.83 | 0.58 | 0.16** | 0.02 | 0.83 | 74.32 |
|  |  | Swim | 0.61 | -16.64 | -0.37 | -16.79 | 0.91 | 0.45 | 0.17** | 0.01 | 0.84 | 74.27 |
|  |  | Tired | 0.66 | -16.52 | -0.24 | -16.67 | 1.01 | 0.58 | 0.17** | 0.00 | 0.83 | 74.98 |
|  |  | water | 0.68 | -17.41 | -0.11 | -17.61 | 1.00 | 0.57 | 0.17** | 0.02 | 0.83 | 74.21 |
| Miami-Dade | None | | -1.58 | -0.34 | 0.46 | -0.05 | 0.46 | -0.84 | - | - | 0.66 | 150.76 |
|  |  |  | -1.64 | -0.40 | 0.36 | -0.24 | 0.48 | -0.86 | 0.27*** | - | 0.74 | 142.89 |
|  | Twitter | Heat | -1.63 | -0.41 | 0.46 | -0.24 | 0.49 | -0.84 | 0.29*** | -0.01 | 0.74 | 144.31 |
|  |  | AC | -1.67 | -0.50 | 0.34 | -0.38 | 0.38 | -0.79 | 0.31*** | -0.07 | 0.75 | 143.79 |
|  | Google Search | AC repair | -1.65 | -0.41 | 0.34 | -0.26 | 0.44 | -0.88 | 0.28*** | 0.00 | 0.74 | 144.82 |
|  |  | Beer | -1.72* | -0.48 | 0.05 | -0.74 | 0.04 | -0.88 | 0.26*** | 0.02 | 0.75 | 143.73 |
|  |  | Drink | -2.17** | -1.10 | -0.28 | -1.45* | -0.54 | -1.23 | 0.29*** | 0.05*** | 0.79 | 137.93 |
|  |  | Heat exhaustion | -1.71* | -0.53 | 0.40 | -0.26 | 0.38 | -0.99 | 0.23*** | 0.01** | 0.76 | 142.10 |
|  |  | Heat stroke | -1.57 | -0.36 | 0.46 | -0.20 | 0.40 | -1.13 | 0.26*** | 0.01** | 0.76 | 142.24 |
|  |  | Hot weather | -1.61 | -0.38 | 0.36 | -0.24 | 0.47 | -0.88 | 0.27*** | 0.00 | 0.74 | 144.67 |
|  |  | Park | -1.65 | -0.44 | 0.13 | -0.63 | 0.17 | -1.01 | 0.24*** | 0.03 | 0.74 | 143.77 |
|  |  | Pool | -1.54 | -0.38 | 0.36 | -0.51 | 0.08 | -1.00 | 0.22*** | 0.03** | 0.76 | 142.03 |
|  |  | Swim | -1.64 | -0.39 | 0.37 | -0.27 | 0.47 | -0.90 | 0.26*** | 0.01 | 0.74 | 144.32 |
|  |  | Tired | -1.63 | -0.45 | 0.31 | -0.22 | 0.49 | -0.92 | 0.28*** | -0.01 | 0.74 | 144.65 |
|  |  | water | -1.72* | -0.41 | 0.22 | -0.43 | 0.18 | -1.17 | 0.21*** | 0.05** | 0.76 | 142.09 |
| Orange | None | | 1.76* | 2.46*** | 1.76* | 0.69 | 1.48 | 1.48 | - | - | 0.67 | 158.10 |
|  |  |  | 1.62 | 2.37*** | 1.56 | 0.44 | 1.33 | 1.34 | 0.22*** | - | 0.76 | 148.94 |
|  | Twitter | Heat | 1.62 | 2.39*** | 1.58 | 0.46 | 1.36 | 1.38 | 0.20*** | 0.01 | 0.77 | 150.80 |
|  |  | AC | 1.64 | 2.40*** | 1.60 | 0.47 | 1.37 | 1.37 | 0.21*** | 0.02 | 0.76 | 150.89 |
|  | Google Search | AC repair | 1.61 | 2.40*** | 1.52 | 0.45 | 1.31 | 1.34 | 0.23*** | 0.00 | 0.76 | 150.81 |
|  |  | Beer | 1.57 | 2.34*** | 1.35 | -0.23 | 0.74 | 1.35 | 0.21*** | 0.02 | 0.76 | 149.70 |
|  |  | Drink | 1.59 | 2.33*** | 1.50 | 0.21 | 1.17 | 1.33 | 0.21*** | 0.01 | 0.76 | 150.73 |
|  |  | Heat exhaustion | 1.60 | 2.36*** | 1.52 | 0.40 | 1.29 | 1.26 | 0.23*** | -0.01 | 0.77 | 150.61 |
|  |  | Heat stroke | 1.59 | 2.33*** | 1.58 | 0.45 | 1.27 | 1.36 | 0.20*** | 0.01 | 0.77 | 150.43 |
|  |  | Hot weather | 1.64 | 2.44*** | 1.62 | 0.43 | 1.36 | 1.39 | 0.22*** | 0.01 | 0.77 | 150.47 |
|  |  | Park | 1.61 | 2.33*** | 1.44 | 0.16 | 1.21 | 1.30 | 0.21*** | 0.02 | 0.76 | 150.45 |
|  |  | Pool | 1.62 | 2.36*** | 1.55 | 0.39 | 1.29 | 1.33 | 0.22*** | 0.00 | 0.76 | 150.92 |
|  |  | Swim | 1.63 | 2.38*** | 1.56 | 0.43 | 1.33 | 1.35 | 0.22*** | 0.00 | 0.76 | 150.94 |
|  |  | Tired | 1.67* | 2.29*** | 1.50 | 0.32 | 1.32 | 1.30 | 0.23*** | -0.01 | 0.77 | 149.74 |
|  |  | water | 1.64 | 2.42*** | 1.55 | 0.22 | 1.02 | 1.05 | 0.17*** | 0.05* | 0.77 | 148.73 |

Table S5. Heat-related-illness ED model specifications (*** <0.05, ** 0.05 ~ 0.10, * 0.10 ~ 0.15)

|  |  |  | Lag 0 | | | Lag 1 | | | Lag 2 | | | Lag 3 | | |
| --- | --- | --- | --- | --- | --- | --- | --- | --- | --- | --- | --- | --- | --- | --- |
|  | Keywords | | keyword | AUC | AIC | keyword | AUC | AIC | keyword | AUC | AIC | keyword | AUC | AIC |
| Duval | Twitter | Heat | 0.14** | 0.82 | 188.43 | 0.22*** | 0.83 | 184.81 | 0.13* | 0.81 | 188.56 | 0.05 | 0.81 | 190.62 |
|  |  | AC | 0.40*** | 0.82 | 185.75 | -0.26** | 0.82 | 188.54 | 0.05 | 0.81 | 191.17 | -0.04 | 0.81 | 191.20 |
|  | Google Search | AC repair | 0.00 | 0.81 | 191.25 | 0.03*** | 0.83 | 183.40 | 0.01 | 0.81 | 190.79 | 0.02*** | 0.82 | 186.61 |
|  |  | Beer | -0.02** | 0.81 | 188.16 | -0.01 | 0.81 | 190.99 | -0.01 | 0.81 | 190.54 | 0.00 | 0.81 | 191.24 |
|  |  | Drink | -0.01 | 0.81 | 190.65 | 0.00 | 0.81 | 191.22 | 0.01 | 0.81 | 189.78 | 0.00 | 0.81 | 191.20 |
|  |  | Heat exhaustion | 0.00 | 0.81 | 191.19 | 0.01 | 0.81 | 190.05 | -0.02*** | 0.83 | 186.21 | 0.01 | 0.81 | 190.45 |
|  |  | Heat stroke | 0.02 | 0.82 | 189.31 | 0.01 | 0.81 | 190.54 | 0.02 | 0.82 | 189.49 | 0.01 | 0.81 | 190.29 |
|  |  | Hot weather | -0.01 | 0.81 | 189.96 | 0.01 | 0.81 | 190.46 | 0.03*** | 0.83 | 183.72 | 0.00 | 0.81 | 191.29 |
|  |  | Park | 0.02 | 0.81 | 190.47 | 0.02 | 0.81 | 190.31 | 0.02 | 0.81 | 190.50 | 0.03* | 0.82 | 188.88 |
|  |  | Pool | 0.02** | 0.82 | 188.07 | 0.01 | 0.81 | 190.42 | 0.01 | 0.81 | 189.68 | 0.02** | 0.82 | 187.96 |
|  |  | Swim | 0.02*** | 0.83 | 186.67 | 0.01 | 0.81 | 190.38 | 0.01 | 0.81 | 189.61 | 0.01 | 0.81 | 189.54 |
|  |  | Tired | 0.03*** | 0.82 | 184.50 | 0.01 | 0.81 | 191.00 | 0.00 | 0.81 | 191.29 | 0.00 | 0.81 | 191.28 |
|  |  | water | 0.00 | 0.81 | 191.27 | 0.01 | 0.81 | 191.19 | -0.01 | 0.81 | 191.10 | 0.01 | 0.81 | 191.19 |
| Hillsborough | Twitter | Heat | 0.07 | 0.80 | 191.52 | 0.03 | 0.79 | 192.86 | 0.04 | 0.79 | 192.61 | -0.02 | 0.80 | 192.87 |
|  |  | AC | 0.30*** | 0.82 | 184.90 | 0.17** | 0.81 | 190.08 | 0.00 | 0.79 | 193.07 | 0.01 | 0.79 | 193.05 |
|  | Google Search | AC repair | 0.02** | 0.80 | 190.16 | 0.02 | 0.80 | 191.63 | 0.02* | 0.80 | 190.70 | 0.01 | 0.79 | 191.81 |
|  |  | Beer | 0.02 | 0.79 | 192.17 | -0.01 | 0.79 | 192.42 | 0.01 | 0.79 | 192.96 | -0.02 | 0.80 | 192.16 |
|  |  | Drink | 0.00 | 0.79 | 193.03 | -0.01 | 0.79 | 192.59 | -0.02 | 0.80 | 192.03 | 0.00 | 0.79 | 193.04 |
|  |  | Heat exhaustion | -0.01 | 0.80 | 192.21 | -0.01 | 0.80 | 192.82 | 0.03*** | 0.81 | 185.95 | 0.02** | 0.81 | 189.24 |
|  |  | Heat stroke | -0.01** | 0.80 | 190.06 | -0.01** | 0.80 | 189.90 | 0.01 | 0.80 | 192.53 | -0.01 | 0.79 | 192.47 |
|  |  | Hot weather | -0.02** | 0.81 | 189.41 | -0.01 | 0.80 | 192.36 | 0.02*** | 0.81 | 188.45 | 0.03*** | 0.81 | 187.25 |
|  |  | Park | 0.00 | 0.79 | 193.06 | 0.00 | 0.79 | 193.07 | 0.02 | 0.80 | 192.46 | 0.02 | 0.80 | 192.58 |
|  |  | Pool | 0.00 | 0.79 | 193.04 | 0.01 | 0.79 | 192.70 | 0.02** | 0.81 | 190.29 | 0.04*** | 0.82 | 186.81 |
|  |  | Swim | 0.01 | 0.79 | 192.71 | 0.02 | 0.80 | 191.17 | 0.01 | 0.80 | 192.17 | 0.01 | 0.79 | 192.54 |
|  |  | Tired | 0.01 | 0.80 | 191.06 | -0.01 | 0.79 | 192.48 | -0.01 | 0.80 | 191.43 | 0.00 | 0.79 | 193.06 |
|  |  | water | 0.02 | 0.80 | 192.47 | -0.01 | 0.79 | 192.95 | 0.03 | 0.80 | 191.19 | 0.04** | 0.80 | 189.79 |
| Leon | Twitter | Heat | -0.01 | 0.76 | 153.75 | -0.05 | 0.77 | 153.04 | 0.00 | 0.76 | 153.76 | -0.01 | 0.76 | 153.71 |
|  |  | AC | 0.12 | 0.76 | 153.36 | 0.16 | 0.76 | 153.06 | -0.13 | 0.76 | 153.34 | -0.14 | 0.76 | 153.22 |
|  | Google Search | AC repair | 0.01 | 0.76 | 153.21 | 0.00 | 0.76 | 153.76 | 0.00 | 0.76 | 153.76 | 0.01 | 0.77 | 153.33 |
|  |  | Beer | -0.02** | 0.79 | 150.45 | -0.02 | 0.77 | 152.06 | -0.01 | 0.76 | 153.19 | -0.02 | 0.77 | 152.48 |
|  |  | Drink | 0.03*** | 0.78 | 150.03 | 0.03*** | 0.78 | 149.90 | -0.03** | 0.79 | 150.09 | 0.02 | 0.77 | 152.75 |
|  |  | Heat exhaustion | -0.27 | 0.78 | 150.58 | -0.26 | 0.78 | 150.83 | 0.01 | 0.76 | 153.59 | -0.26 | 0.77 | 151.17 |
|  |  | Heat stroke | 0.00 | 0.76 | 153.74 | -0.02 | 0.77 | 151.72 | 0.01 | 0.77 | 153.05 | 0.00 | 0.76 | 153.71 |
|  |  | Hot weather | 0.01 | 0.76 | 153.19 | 0.00 | 0.76 | 153.61 | -0.02* | 0.77 | 150.40 | -0.01 | 0.77 | 153.31 |
|  |  | Park | 0.00 | 0.76 | 153.74 | 0.00 | 0.76 | 153.76 | -0.01 | 0.76 | 153.59 | 0.01 | 0.76 | 153.17 |
|  |  | Pool | 0.00 | 0.76 | 153.68 | 0.00 | 0.76 | 153.76 | -0.02 | 0.77 | 151.77 | 0.00 | 0.76 | 153.67 |
|  |  | Swim | -0.01 | 0.78 | 152.57 | -0.01 | 0.77 | 153.20 | -0.01 | 0.76 | 152.81 | 0.01 | 0.77 | 153.34 |
|  |  | Tired | 0.01 | 0.77 | 153.16 | 0.00 | 0.76 | 153.75 | -0.01 | 0.76 | 152.87 | 0.00 | 0.76 | 153.66 |
|  |  | water | -0.01 | 0.76 | 153.50 | 0.01 | 0.76 | 153.60 | -0.01 | 0.77 | 153.13 | -0.02 | 0.77 | 152.12 |
| Miami-Dade | Twitter | Heat | 0.00 | 0.75 | 215.11 | 0.00 | 0.75 | 215.14 | -0.01 | 0.75 | 214.27 | -0.01 | 0.76 | 214.43 |
|  |  | AC | 0.04 | 0.75 | 214.55 | 0.01 | 0.75 | 215.24 | -0.01 | 0.75 | 215.22 | 0.01 | 0.75 | 215.14 |
|  | Google Search | AC repair | 0.01 | 0.75 | 214.87 | 0.00 | 0.75 | 215.12 | -0.01 | 0.75 | 214.86 | 0.00 | 0.75 | 215.09 |
|  |  | Beer | 0.01 | 0.75 | 214.95 | 0.01 | 0.76 | 214.76 | 0.00 | 0.75 | 215.26 | 0.00 | 0.75 | 215.24 |
|  |  | Drink | 0.01 | 0.75 | 214.86 | 0.04*** | 0.77 | 209.61 | 0.00 | 0.75 | 215.26 | 0.02 | 0.76 | 214.00 |
|  |  | Heat exhaustion | 0.00 | 0.76 | 215.00 | 0.02*** | 0.76 | 211.01 | -0.01** | 0.76 | 211.89 | 0.01 | 0.76 | 213.34 |
|  |  | Heat stroke | 0.00 | 0.75 | 215.24 | 0.00 | 0.76 | 215.16 | -0.02*** | 0.77 | 210.79 | 0.00 | 0.75 | 215.27 |
|  |  | Hot weather | 0.00 | 0.75 | 215.27 | 0.00 | 0.76 | 214.80 | -0.01 | 0.76 | 214.12 | -0.01*** | 0.77 | 211.41 |
|  |  | Park | 0.02 | 0.75 | 214.53 | 0.02 | 0.76 | 214.10 | 0.01 | 0.75 | 214.87 | 0.03 | 0.76 | 213.47 |
|  |  | Pool | 0.01 | 0.75 | 214.72 | 0.00 | 0.75 | 215.24 | -0.01 | 0.75 | 214.90 | 0.01 | 0.75 | 215.03 |
|  |  | Swim | 0.01 | 0.75 | 214.68 | 0.02** | 0.76 | 211.96 | 0.01 | 0.75 | 214.69 | 0.00 | 0.75 | 215.07 |
|  |  | Tired | 0.00 | 0.75 | 215.25 | 0.00 | 0.75 | 215.27 | 0.01 | 0.75 | 215.09 | -0.01 | 0.75 | 214.91 |
|  |  | Water | 0.03 | 0.75 | 213.82 | 0.00 | 0.75 | 215.26 | -0.02 | 0.75 | 214.52 | 0.00 | 0.75 | 215.27 |
| Orange | Twitter | Heat | 0.03 | 0.76 | 212.86 | 0.02 | 0.76 | 213.10 | 0.00 | 0.76 | 213.55 | 0.00 | 0.76 | 213.52 |
|  |  | AC | 0.03 | 0.76 | 213.39 | 0.02 | 0.76 | 213.48 | 0.03 | 0.76 | 213.35 | 0.13** | 0.77 | 210.36 |
|  | Google Search | AC repair | 0.00 | 0.76 | 213.43 | -0.01 | 0.76 | 212.80 | -0.01 | 0.77 | 212.74 | 0.01 | 0.76 | 211.91 |
|  |  | Beer | 0.03* | 0.76 | 211.29 | 0.01 | 0.76 | 213.16 | 0.00 | 0.76 | 213.55 | 0.00 | 0.76 | 213.49 |
|  |  | Drink | 0.02 | 0.76 | 212.40 | 0.01 | 0.76 | 213.14 | 0.00 | 0.76 | 213.51 | 0.02 | 0.76 | 212.03 |
|  |  | Heat exhaustion | 0.01* | 0.76 | 211.43 | 0.02*** | 0.79 | 206.16 | -0.01 | 0.76 | 212.44 | -0.02*** | 0.77 | 208.48 |
|  |  | Heat stroke | 0.02** | 0.76 | 210.42 | -0.01 | 0.76 | 212.51 | -0.01 | 0.76 | 211.72 | 0.00 | 0.76 | 213.30 |
|  |  | Hot weather | 0.00 | 0.76 | 213.32 | 0.00 | 0.77 | 213.50 | -0.01 | 0.77 | 212.80 | -0.01* | 0.77 | 211.18 |
|  |  | Park | 0.00 | 0.76 | 213.51 | 0.00 | 0.76 | 213.54 | 0.00 | 0.76 | 213.52 | 0.02 | 0.76 | 212.94 |
|  |  | Pool | 0.01 | 0.76 | 213.19 | 0.00 | 0.76 | 213.52 | -0.01 | 0.77 | 212.80 | 0.00 | 0.76 | 213.55 |
|  |  | Swim | 0.00 | 0.76 | 213.50 | 0.00 | 0.76 | 213.43 | 0.00 | 0.76 | 213.47 | 0.01 | 0.76 | 212.63 |
|  |  | Tired | -0.02** | 0.77 | 210.84 | 0.01 | 0.76 | 212.59 | -0.01 | 0.77 | 212.37 | 0.02** | 0.78 | 210.51 |
|  |  | water | 0.04** | 0.76 | 210.87 | 0.03 | 0.75 | 211.87 | 0.00 | 0.76 | 213.53 | 0.03 | 0.77 | 211.61 |

Table S6. Heat-related-illness hospitalization model specifications (*** <0.05, ** 0.05 ~ 0.10, * 0.10 ~ 0.15)

|  |  |  | Lag 0 | | | Lag 1 | | | Lag 2 | | | Lag 3 | | |
| --- | --- | --- | --- | --- | --- | --- | --- | --- | --- | --- | --- | --- | --- | --- |
|  | Keywords | | keyword | AUC | AIC | keyword | AUC | AIC | keyword | AUC | AIC | keyword | AUC | AIC |
| Duval | Twitter | Heat | 0.15** | 0.82 | 150.66 | -0.07 | 0.81 | 153.39 | -0.01 | 0.81 | 154.24 | -0.01 | 0.81 | 154.20 |
|  |  | AC | 0.24* | 0.82 | 151.84 | 0.10 | 0.82 | 153.79 | 0.16 | 0.82 | 153.26 | 0.02 | 0.81 | 154.24 |
|  | Google Search | AC repair | 0.00 | 0.81 | 154.16 | 0.00 | 0.81 | 154.19 | 0.00 | 0.81 | 154.21 | 0.01 | 0.81 | 153.78 |
|  |  | Beer | -0.02 | 0.82 | 152.15 | -0.01 | 0.82 | 153.61 | 0.00 | 0.81 | 154.20 | -0.03** | 0.82 | 150.44 |
|  |  | Drink | 0.01 | 0.82 | 153.86 | -0.01 | 0.82 | 153.57 | 0.01 | 0.81 | 153.60 | -0.01 | 0.81 | 153.91 |
|  |  | Heat exhaustion | 0.00 | 0.81 | 154.24 | 0.00 | 0.81 | 153.93 | -0.01 | 0.82 | 152.54 | 0.01 | 0.82 | 153.15 |
|  |  | Heat stroke | 0.00 | 0.81 | 154.16 | -0.02 | 0.82 | 152.59 | -0.01 | 0.82 | 153.19 | 0.00 | 0.81 | 154.24 |
|  |  | Hot weather | -0.02** | 0.83 | 150.50 | 0.01 | 0.81 | 153.59 | 0.00 | 0.81 | 154.18 | 0.00 | 0.81 | 154.19 |
|  |  | Park | 0.02 | 0.81 | 153.41 | 0.07*** | 0.85 | 144.92 | 0.03* | 0.82 | 151.92 | 0.02 | 0.82 | 153.49 |
|  |  | Pool | 0.01 | 0.81 | 153.66 | 0.02** | 0.82 | 151.45 | 0.02** | 0.83 | 151.49 | 0.02 | 0.82 | 152.70 |
|  |  | Swim | 0.01 | 0.81 | 152.98 | 0.01 | 0.81 | 153.93 | 0.00 | 0.82 | 154.12 | 0.00 | 0.81 | 154.24 |
|  |  | Tired | -0.01 | 0.81 | 154.02 | -0.02 | 0.82 | 152.30 | 0.01 | 0.81 | 153.80 | 0.01 | 0.82 | 152.89 |
|  |  | water | -0.01 | 0.81 | 154.06 | 0.00 | 0.81 | 154.25 | 0.04** | 0.82 | 151.02 | 0.02 | 0.81 | 152.92 |
| Hillsborough | Twitter | Heat | 0.17*** | 0.78 | 173.30 | 0.03 | 0.76 | 183.72 | 0.06 | 0.76 | 182.66 | 0.00 | 0.76 | 184.21 |
|  |  | AC | 0.20*** | 0.77 | 179.99 | 0.31*** | 0.79 | 173.89 | 0.09 | 0.76 | 183.35 | 0.07 | 0.76 | 183.75 |
|  | Google Search | AC repair | 0.00 | 0.76 | 184.12 | 0.04*** | 0.79 | 173.47 | 0.00 | 0.76 | 184.10 | 0.01 | 0.75 | 183.29 |
|  |  | Beer | 0.00 | 0.76 | 184.18 | 0.04*** | 0.77 | 180.29 | -0.02 | 0.76 | 183.09 | 0.00 | 0.76 | 184.20 |
|  |  | Drink | 0.02 | 0.77 | 182.25 | 0.01 | 0.76 | 183.80 | -0.01 | 0.76 | 184.09 | 0.05*** | 0.79 | 176.32 |
|  |  | Heat exhaustion | -0.02** | 0.77 | 180.87 | -0.03*** | 0.78 | 177.74 | 0.01 | 0.77 | 182.41 | 0.01 | 0.76 | 183.03 |
|  |  | Heat stroke | 0.02*** | 0.77 | 179.68 | -0.01 | 0.76 | 183.24 | 0.00 | 0.76 | 183.93 | -0.02** | 0.77 | 180.67 |
|  |  | Hot weather | 0.00 | 0.76 | 184.14 | -0.01 | 0.77 | 182.72 | 0.02* | 0.77 | 181.88 | 0.01 | 0.76 | 183.64 |
|  |  | Park | 0.05*** | 0.77 | 180.08 | 0.04* | 0.76 | 181.91 | 0.06*** | 0.77 | 177.88 | 0.07*** | 0.78 | 175.91 |
|  |  | Pool | 0.02 | 0.76 | 182.39 | 0.02* | 0.76 | 181.95 | 0.06*** | 0.80 | 171.99 | 0.04*** | 0.77 | 178.94 |
|  |  | Swim | 0.02* | 0.76 | 181.91 | 0.04*** | 0.78 | 177.55 | 0.04*** | 0.77 | 177.31 | 0.03*** | 0.77 | 179.64 |
|  |  | Tired | 0.00 | 0.76 | 184.18 | 0.00 | 0.76 | 184.18 | 0.00 | 0.76 | 184.14 | -0.01 | 0.76 | 182.79 |
|  |  | water | 0.03 | 0.76 | 182.93 | 0.05** | 0.76 | 180.46 | 0.06*** | 0.76 | 179.75 | 0.05** | 0.76 | 180.89 |
| Leon | Twitter | Heat | 0.05 | 0.83 | 74.81 | 0.02 | 0.82 | 74.97 | 0.04 | 0.82 | 74.76 | 0.07 | 0.81 | 74.47 |
|  |  | AC | 0.18 | 0.82 | 74.70 | 0.21 | 0.83 | 74.55 | 0.38 | 0.82 | 73.40 | 0.36 | 0.83 | 73.42 |
|  | Google Search | AC repair | 0.00 | 0.83 | 74.93 | -0.38 | 0.85 | 71.55 | 0.00 | 0.83 | 74.96 | -0.01 | 0.84 | 74.44 |
|  |  | Beer | -0.02 | 0.84 | 74.51 | -0.02 | 0.84 | 74.36 | -0.02 | 0.83 | 74.13 | -0.01 | 0.83 | 74.91 |
|  |  | Drink | 0.03 | 0.84 | 73.61 | 0.02 | 0.83 | 74.25 | -0.01 | 0.83 | 74.78 | 0.00 | 0.83 | 74.96 |
|  |  | Heat exhaustion | -0.30 | 0.84 | 73.43 | -0.25 | 0.83 | 74.67 | -0.29 | 0.83 | 74.31 | -0.19 | 0.83 | 74.91 |
|  |  | Heat stroke | 0.01 | 0.84 | 74.31 | 0.01 | 0.82 | 74.59 | 0.00 | 0.82 | 74.98 | 0.02 | 0.83 | 73.69 |
|  |  | Hot weather | 0.00 | 0.83 | 74.98 | 0.02 | 0.81 | 73.58 | 0.00 | 0.82 | 74.97 | 0.01 | 0.83 | 74.32 |
|  |  | Park | 0.06*** | 0.88 | 71.27 | 0.06** | 0.85 | 71.75 | 0.03 | 0.83 | 73.29 | 0.05*** | 0.84 | 70.96 |
|  |  | Pool | 0.02 | 0.83 | 74.32 | 0.02 | 0.85 | 73.18 | 0.02 | 0.84 | 73.76 | 0.01 | 0.83 | 74.90 |
|  |  | Swim | 0.01 | 0.84 | 74.27 | 0.04*** | 0.87 | 69.58 | 0.03** | 0.86 | 71.89 | 0.02 | 0.85 | 73.78 |
|  |  | Tired | 0.00 | 0.83 | 74.98 | 0.00 | 0.83 | 74.99 | 0.01 | 0.83 | 74.79 | -0.01 | 0.83 | 74.79 |
|  |  | water | 0.02 | 0.83 | 74.21 | -0.01 | 0.83 | 74.87 | 0.00 | 0.82 | 74.96 | 0.00 | 0.83 | 74.98 |
| Miami-Dade | Twitter | Heat | -0.01 | 0.74 | 144.31 | 0.01* | 0.76 | 142.80 | 0.01 | 0.75 | 143.77 | 0.01 | 0.74 | 144.53 |
|  |  | AC | -0.07 | 0.75 | 143.79 | -0.04 | 0.74 | 144.50 | 0.01 | 0.74 | 144.83 | 0.02 | 0.74 | 144.64 |
|  | Google Search | AC repair | 0.00 | 0.74 | 144.82 | 0.00 | 0.74 | 144.73 | 0.00 | 0.74 | 144.87 | -0.01 | 0.75 | 144.29 |
|  |  | Beer | 0.02 | 0.75 | 143.73 | 0.01 | 0.75 | 144.69 | -0.03 | 0.75 | 143.03 | -0.04** | 0.77 | 141.09 |
|  |  | Drink | 0.05*** | 0.79 | 137.93 | -0.02 | 0.75 | 144.13 | -0.01 | 0.74 | 144.63 | -0.03 | 0.76 | 142.80 |
|  |  | Heat exhaustion | 0.01** | 0.76 | 142.10 | 0.00 | 0.74 | 144.72 | 0.00 | 0.74 | 144.87 | -0.01 | 0.74 | 144.46 |
|  |  | Heat stroke | 0.01** | 0.76 | 142.24 | 0.00 | 0.74 | 144.74 | 0.01 | 0.74 | 144.48 | 0.00 | 0.74 | 144.85 |
|  |  | Hot weather | 0.00 | 0.74 | 144.67 | -0.01 | 0.75 | 144.54 | -0.01 | 0.74 | 143.21 | 0.01 | 0.75 | 143.14 |
|  |  | Park | 0.03 | 0.74 | 143.77 | 0.03 | 0.75 | 143.51 | 0.02 | 0.74 | 144.16 | 0.02 | 0.75 | 144.10 |
|  |  | Pool | 0.03** | 0.76 | 142.03 | 0.00 | 0.74 | 144.88 | 0.00 | 0.74 | 144.89 | 0.00 | 0.74 | 144.88 |
|  |  | Swim | 0.01 | 0.74 | 144.32 | 0.00 | 0.74 | 144.85 | 0.01 | 0.74 | 144.61 | 0.00 | 0.74 | 144.89 |
|  |  | Tired | -0.01 | 0.74 | 144.65 | -0.01 | 0.74 | 144.59 | 0.01 | 0.74 | 144.17 | 0.02 | 0.74 | 143.78 |
|  |  | Water | 0.05** | 0.76 | 142.09 | 0.00 | 0.74 | 144.88 | 0.01 | 0.74 | 144.77 | -0.04 | 0.75 | 143.32 |
| Orange | Twitter | Heat | 0.01 | 0.77 | 150.80 | 0.02 | 0.76 | 150.55 | 0.00 | 0.76 | 150.93 | 0.04* | 0.79 | 148.77 |
|  |  | AC | 0.02 | 0.76 | 150.89 | 0.20*** | 0.80 | 145.08 | 0.06 | 0.77 | 150.44 | 0.06 | 0.76 | 150.40 |
|  | Google Search | AC repair | 0.00 | 0.76 | 150.81 | -0.01 | 0.77 | 149.85 | -0.03*** | 0.78 | 146.16 | 0.00 | 0.76 | 150.93 |
|  |  | Beer | 0.02 | 0.76 | 149.70 | 0.01 | 0.76 | 150.76 | -0.01 | 0.76 | 150.85 | 0.01 | 0.76 | 150.78 |
|  |  | Drink | 0.01 | 0.76 | 150.73 | 0.02 | 0.77 | 150.37 | -0.03* | 0.78 | 148.65 | 0.03 | 0.77 | 148.98 |
|  |  | Heat exhaustion | -0.01 | 0.77 | 150.61 | 0.00 | 0.76 | 150.78 | 0.00 | 0.76 | 150.85 | 0.01 | 0.76 | 150.67 |
|  |  | Heat stroke | 0.01 | 0.77 | 150.43 | 0.00 | 0.76 | 150.71 | -0.02 | 0.77 | 149.17 | 0.01 | 0.77 | 149.96 |
|  |  | Hot weather | 0.01 | 0.77 | 150.47 | 0.00 | 0.76 | 150.93 | -0.01 | 0.76 | 150.42 | 0.00 | 0.76 | 150.94 |
|  |  | Park | 0.02 | 0.76 | 150.45 | 0.00 | 0.76 | 150.93 | 0.03 | 0.77 | 149.88 | 0.02 | 0.76 | 150.17 |
|  |  | Pool | 0.00 | 0.76 | 150.92 | 0.01 | 0.76 | 150.82 | 0.01 | 0.76 | 150.71 | 0.03* | 0.77 | 148.57 |
|  |  | Swim | 0.00 | 0.76 | 150.94 | 0.02* | 0.77 | 148.57 | 0.01 | 0.76 | 150.78 | 0.02* | 0.77 | 148.32 |
|  |  | Tired | -0.01 | 0.77 | 149.74 | -0.02 | 0.77 | 149.14 | 0.01 | 0.76 | 150.71 | 0.00 | 0.76 | 150.82 |
|  |  | water | 0.05* | 0.77 | 148.73 | -0.02 | 0.77 | 150.58 | 0.03 | 0.76 | 150.22 | 0.07*** | 0.78 | 146.16 |

Table S7. Dehydration ED model specifications (*** <0.05, ** 0.05 ~ 0.10, * 0.10 ~ 0.15)

| County | Keywords | | Wed | Thu | Fri | Sat | Sun | Mon | Max T | keyword | MAE | AIC |
| --- | --- | --- | --- | --- | --- | --- | --- | --- | --- | --- | --- | --- |
| Duval | None | | 0.03 | 0.01 | -0.04 | -0.11 | -0.09 | -0.06 | - | - | 3.58 | 985.25 |
|  |  |  | 0.02 | -0.01 | -0.05 | -0.12* | -0.09 | -0.05 | 0.01*** | - | 3.51 | 977.07 |
|  | Twitter | Heat | 0.02 | 0.00 | -0.05 | -0.13* | -0.07 | -0.06 | 0.01** | 0.02*** | 3.47 | 971.72 |
|  |  | AC | 0.00 | -0.01 | -0.07 | -0.13* | -0.10 | -0.05 | 0.01*** | 0.04*** | 3.43 | 973.98 |
|  | Google Search | AC repair | 0.01 | -0.01 | -0.06 | -0.13* | -0.09 | -0.06 | 0.01*** | 0.00 | 3.51 | 978.76 |
|  |  | Beer | 0.02 | -0.01 | -0.05 | -0.10 | -0.07 | -0.05 | 0.01*** | 0.00 | 3.51 | 978.67 |
|  |  | Drink | 0.01 | -0.01 | -0.06 | -0.13* | -0.10 | -0.05 | 0.01*** | 0.00 | 3.50 | 978.72 |
|  |  | Heat exhaustion | 0.03 | -0.01 | -0.05 | -0.11 | -0.09 | -0.05 | 0.01*** | 0.00 | 3.47 | 977.29 |
|  |  | Heat stroke | 0.03 | -0.01 | -0.06 | -0.09 | -0.11 | -0.07 | 0.01*** | 0.00*** | 3.37 | 967.12 |
|  |  | Hot weather | 0.02 | -0.01 | -0.05 | -0.12* | -0.09 | -0.05 | 0.01*** | 0.00 | 3.51 | 978.96 |
|  |  | Park | 0.01 | -0.01 | -0.09 | -0.17*** | -0.11 | -0.07 | 0.01*** | 0.00*** | 3.44 | 975.10 |
|  |  | Pool | 0.03 | -0.02 | -0.06 | -0.16*** | -0.12* | -0.06 | 0.01*** | 0.00*** | 3.40 | 973.45 |
|  |  | Swim | 0.03 | -0.01 | -0.03 | -0.12* | -0.09 | -0.05 | 0.01*** | 0.00** | 3.47 | 975.82 |
|  |  | Tired | 0.03 | 0.01 | -0.05 | -0.11 | -0.07 | -0.05 | 0.01*** | 0.00 | 3.50 | 977.33 |
|  |  | water | 0.02 | 0.00 | -0.05 | -0.11 | -0.08 | -0.05 | 0.01*** | 0.00 | 3.50 | 978.50 |
| Hillsborough | None | | 0.00 | 0.02 | 0.08 | 0.03 | 0.04 | 0.04 | - | - | 3.54 | 994.33 |
|  |  |  | -0.01 | 0.01 | 0.08 | 0.03 | 0.05 | 0.04 | 0.01*** | - | 3.50 | 990.57 |
|  | Twitter | Heat | -0.01 | 0.01 | 0.07 | 0.03 | 0.05 | 0.04 | 0.01* | 0.01* | 3.47 | 990.05 |
|  |  | AC | -0.01 | 0.01 | 0.08 | 0.04 | 0.06 | 0.04 | 0.01** | 0.02*** | 3.40 | 988.18 |
|  | Google Search | AC repair | -0.01 | 0.01 | 0.08 | 0.02 | 0.05 | 0.04 | 0.01*** | 0.00 | 3.50 | 992.38 |
|  |  | Beer | -0.01 | 0.02 | 0.10 | 0.06 | 0.07 | 0.05 | 0.01*** | 0.00 | 3.48 | 992.10 |
|  |  | Drink | -0.01 | 0.01 | 0.08 | 0.03 | 0.05 | 0.04 | 0.01*** | 0.00 | 3.50 | 992.50 |
|  |  | Heat exhaustion | 0.00 | 0.03 | 0.08 | 0.03 | 0.04 | 0.03 | 0.01** | 0.00 | 3.49 | 990.90 |
|  |  | Heat stroke | -0.01 | 0.02 | 0.08 | 0.04 | 0.06 | 0.05 | 0.01*** | 0.00 | 3.49 | 990.66 |
|  |  | Hot weather | -0.01 | 0.02 | 0.09 | 0.03 | 0.05 | 0.05 | 0.01*** | 0.00*** | 3.51 | 988.65 |
|  |  | Park | 0.00 | 0.01 | 0.02 | -0.08 | -0.02 | 0.03 | 0.01*** | 0.01*** | 3.49 | 985.47 |
|  |  | Pool | 0.00 | 0.00 | 0.07 | -0.05 | -0.02 | 0.00 | 0.01 | 0.01*** | 3.43 | 982.04 |
|  |  | Swim | -0.02 | 0.03 | 0.08 | 0.00 | 0.03 | 0.02 | 0.01* | 0.00*** | 3.44 | 985.04 |
|  |  | Tired | -0.01 | 0.01 | 0.08 | 0.03 | 0.05 | 0.05 | 0.01*** | 0.00 | 3.51 | 991.79 |
|  |  | water | 0.00 | 0.01 | 0.08 | 0.04 | 0.04 | 0.02 | 0.01 | 0.01*** | 3.42 | 983.49 |
| Leon | None | | -0.43 | -0.22 | -0.55 | -0.68 | -1.68*** | 0.45 | - | - | 2.00 | 791.77 |
|  |  |  | -0.43 | -0.22 | -0.56 | -0.69 | -1.67*** | 0.45 | 0.01 | - | 2.00 | 793.65 |
|  | Twitter | Heat | -0.43 | -0.22 | -0.56 | -0.69 | -1.67*** | 0.45 | 0.01 | 0.00 | 2.00 | 795.64 |
|  |  | AC | -0.43 | -0.22 | -0.56 | -0.69 | -1.66*** | 0.45 | 0.01 | 0.02 | 2.00 | 795.64 |
|  | Google Search | AC repair | -0.43 | -0.27 | -0.55 | -0.72 | -1.67*** | 0.44 | 0.01 | 0.00 | 2.00 | 795.44 |
|  |  | Beer | -0.48 | -0.41 | -0.76 | -0.98 | -1.82*** | 0.44 | 0.01 | 0.02* | 1.98 | 793.16 |
|  |  | Drink | -0.40 | -0.22 | -0.52 | -0.55 | -1.52*** | 0.44 | 0.01 | -0.01 | 2.01 | 795.01 |
|  |  | Heat exhaustion | -0.41 | -0.22 | -0.56 | -0.69 | -1.66*** | 0.47 | 0.01 | 0.00 | 2.00 | 795.60 |
|  |  | Heat stroke | -0.43 | -0.22 | -0.56 | -0.69 | -1.67*** | 0.45 | 0.01 | 0.00 | 2.00 | 795.65 |
|  |  | Hot weather | -0.30 | -0.06 | -0.37 | -0.62 | -1.68*** | 0.55 | 0.02 | -0.02*** | 1.96 | 791.19 |
|  |  | Park | -0.44 | -0.16 | -0.50 | -0.62 | -1.73*** | 0.51 | 0.02 | -0.02 | 2.00 | 794.54 |
|  |  | Pool | -0.43 | -0.22 | -0.53 | -0.78 | -1.75*** | 0.45 | 0.00 | 0.01 | 2.01 | 795.08 |
|  |  | Swim | -0.43 | -0.25 | -0.58 | -0.71 | -1.70*** | 0.43 | 0.01 | 0.00 | 2.01 | 795.54 |
|  |  | Tired | -0.43 | -0.24 | -0.58 | -0.69 | -1.69*** | 0.43 | 0.01 | 0.00 | 2.01 | 795.43 |
|  |  | water | -0.46 | -0.13 | -0.44 | -0.56 | -1.64*** | 0.41 | 0.00 | 0.02* | 2.00 | 792.94 |
| Miami-Dade | None | | 0.00 | 0.03 | 0.03 | 0.01 | -0.02 | 0.20*** | - | - | 4.58 | 1057.99 |
|  |  |  | 0.01 | 0.03 | 0.02 | -0.01 | -0.02 | 0.20*** | 0.02*** | - | 4.38 | 1051.74 |
|  | Twitter | Heat | 0.00 | 0.03 | 0.01 | -0.01 | -0.02 | 0.20*** | 0.02*** | 0.00 | 4.39 | 1052.89 |
|  |  | AC | 0.00 | 0.03 | 0.02 | 0.00 | -0.01 | 0.20*** | 0.02*** | 0.00 | 4.37 | 1053.33 |
|  | Google Search | AC repair | 0.00 | 0.03 | 0.02 | -0.01 | -0.03 | 0.20*** | 0.02*** | 0.00 | 4.42 | 1052.39 |
|  |  | Beer | 0.00 | 0.02 | -0.01 | -0.06 | -0.06 | 0.20*** | 0.01*** | 0.00** | 4.37 | 1050.81 |
|  |  | Drink | 0.01 | 0.03 | 0.03 | 0.01 | 0.00 | 0.21*** | 0.02*** | 0.00 | 4.38 | 1053.23 |
|  |  | Heat exhaustion | 0.00 | 0.03 | 0.02 | -0.01 | -0.02 | 0.20*** | 0.02*** | 0.00 | 4.41 | 1053.24 |
|  |  | Heat stroke | 0.00 | 0.03 | 0.02 | -0.01 | -0.02 | 0.21*** | 0.02*** | 0.00 | 4.39 | 1053.34 |
|  |  | Hot weather | 0.01 | 0.03 | 0.02 | -0.01 | -0.02 | 0.20*** | 0.02*** | 0.00 | 4.38 | 1053.55 |
|  |  | Park | 0.00 | 0.02 | -0.03 | -0.09 | -0.09 | 0.17*** | 0.01** | 0.01*** | 4.28 | 1045.89 |
|  |  | Pool | 0.01 | 0.03 | 0.02 | -0.04 | -0.07 | 0.19*** | 0.01* | 0.00*** | 4.25 | 1046.55 |
|  |  | Swim | 0.00 | 0.03 | 0.02 | -0.01 | -0.02 | 0.20*** | 0.01*** | 0.00* | 4.32 | 1051.52 |
|  |  | Tired | 0.01 | 0.02 | 0.02 | -0.01 | -0.02 | 0.20*** | 0.02*** | 0.00 | 4.37 | 1053.42 |
|  |  | water | 0.00 | 0.03 | 0.01 | -0.02 | -0.05 | 0.18*** | 0.01* | 0.01*** | 4.27 | 1047.30 |
| Orange | None | | 1.59* | -0.04 | 1.22 | 0.55 | -0.33 | 0.88 | - | - | 2.92 | 917.91 |
|  |  |  | 1.52 | -0.12 | 1.14 | 0.51 | -0.32 | 0.86 | 0.08 | - | 2.93 | 918.58 |
|  | Twitter | Heat | 1.52 | -0.15 | 1.12 | 0.50 | -0.33 | 0.84 | 0.11 | -0.03 | 2.93 | 920.15 |
|  |  | AC | 1.52 | -0.12 | 1.14 | 0.51 | -0.32 | 0.87 | 0.07 | 0.00 | 2.93 | 920.58 |
|  | Google Search | AC repair | 1.55* | -0.36 | 1.49 | 0.57 | -0.08 | 0.90 | 0.01 | 0.04*** | 2.85 | 915.21 |
|  |  | Beer | 1.54 | -0.10 | 1.31 | 1.04 | 0.15 | 0.84 | 0.09 | -0.02 | 2.95 | 920.13 |
|  |  | Drink | 1.60* | -0.03 | 1.27 | 0.98 | 0.00 | 0.87 | 0.10 | -0.02 | 2.94 | 920.01 |
|  |  | Heat exhaustion | 1.50 | -0.16 | 1.06 | 0.45 | -0.35 | 0.75 | 0.09 | -0.01 | 2.93 | 920.04 |
|  |  | Heat stroke | 1.40 | -0.31 | 1.15 | 0.48 | -0.47 | 0.82 | 0.04 | 0.02** | 2.88 | 917.79 |
|  |  | Hot weather | 1.54 | -0.17 | 1.11 | 0.59 | -0.31 | 0.84 | 0.08 | -0.01 | 2.92 | 920.01 |
|  |  | Park | 1.51 | -0.06 | 1.33 | 1.01 | -0.12 | 0.93 | 0.09 | -0.03 | 2.94 | 919.80 |
|  |  | Pool | 1.52 | -0.13 | 1.13 | 0.46 | -0.38 | 0.84 | 0.07 | 0.00 | 2.93 | 920.55 |
|  |  | Swim | 1.51 | -0.14 | 1.13 | 0.52 | -0.31 | 0.86 | 0.08 | 0.00 | 2.93 | 920.55 |
|  |  | Tired | 1.56 | -0.23 | 1.02 | 0.39 | -0.38 | 0.77 | 0.08 | -0.02 | 2.93 | 919.66 |
|  |  | water | 1.47 | -0.17 | 1.10 | 0.56 | -0.17 | 1.02 | 0.11 | -0.03 | 2.96 | 919.92 |

Table S8. Dehydration hospitalization model specifications (*** <0.05, ** 0.05 ~ 0.10, * 0.10 ~ 0.15)

| County | Keywords | | Wed | Thu | Fri | Sat | Sun | Mon | Max T | keyword | MAE | AIC |
| --- | --- | --- | --- | --- | --- | --- | --- | --- | --- | --- | --- | --- |
| Duval | None | | -1.48 | -1.74 | -0.77 | -2.61** | -4.61*** | 1.52 | - | - | 3.99 | 1026.48 |
|  |  |  | -1.64 | -1.91 | -0.95 | -2.71** | -4.58*** | 1.59 | 0.16*** | - | 3.86 | 1022.69 |
|  | Twitter | Heat | -1.64 | -1.84 | -0.88 | -2.74** | -4.45*** | 1.47 | 0.11 | 0.24** | 3.79 | 1021.79 |
|  |  | AC | -1.62 | -1.91 | -0.92 | -2.70** | -4.57*** | 1.59 | 0.16*** | -0.05 | 3.86 | 1024.67 |
|  | Google Search | AC repair | -1.64 | -1.90 | -0.91 | -2.67** | -4.56*** | 1.63 | 0.16*** | 0.00 | 3.86 | 1024.68 |
|  |  | Beer | -1.64 | -1.93 | -0.88 | -2.55* | -4.44*** | 1.59 | 0.17*** | -0.01 | 3.85 | 1024.57 |
|  |  | Drink | -1.64 | -1.91 | -0.95 | -2.71** | -4.59*** | 1.60 | 0.16*** | 0.00 | 3.86 | 1024.69 |
|  |  | Heat exhaustion | -1.61 | -1.90 | -0.94 | -2.68** | -4.58*** | 1.60 | 0.16*** | 0.00 | 3.87 | 1024.63 |
|  |  | Heat stroke | -1.72 | -1.92 | -0.93 | -2.83** | -4.50*** | 1.68 | 0.18*** | -0.02 | 3.86 | 1023.95 |
|  |  | Hot weather | -1.66 | -1.94 | -0.92 | -2.62** | -4.53*** | 1.62 | 0.16*** | -0.01 | 3.86 | 1024.31 |
|  |  | Park | -1.63 | -1.92 | -0.79 | -2.45* | -4.50*** | 1.68 | 0.18*** | -0.02 | 3.87 | 1024.35 |
|  |  | Pool | -1.70 | -1.88 | -0.93 | -2.47* | -4.38*** | 1.63 | 0.18*** | -0.02 | 3.86 | 1024.07 |
|  |  | Swim | -1.76 | -1.93 | -1.16 | -2.67** | -4.52*** | 1.54 | 0.18*** | -0.02 | 3.87 | 1023.57 |
|  |  | Tired | -1.34 | -1.51 | -0.84 | -2.47** | -4.35*** | 1.68 | 0.16*** | -0.03 | 3.86 | 1022.90 |
|  |  | water | -1.46 | -1.70 | -0.97 | -2.46** | -4.33*** | 1.79 | 0.18*** | -0.05 | 3.83 | 1022.56 |
| Hillsborough | None | | 1.25 | -0.30 | 0.13 | -3.50*** | -5.00*** | 0.63 | - | - | 3.82 | 1003.65 |
|  |  |  | 1.17 | -0.40 | 0.15 | -3.47*** | -4.95*** | 0.68 | 0.12 | - | 3.78 | 1003.89 |
|  | Twitter | Heat | 1.11 | -0.49 | -0.09 | -3.52*** | -4.94*** | 0.60 | 0.07 | 0.13 | 3.72 | 1004.09 |
|  |  | AC | 1.02 | -0.43 | 0.12 | -3.24*** | -4.77*** | 0.55 | 0.05 | 0.49*** | 3.65 | 999.36 |
|  | Google Search | AC repair | 1.29 | -0.17 | 0.33 | -3.00*** | -4.82*** | 0.87 | 0.17** | -0.04 | 3.77 | 1003.90 |
|  |  | Beer | 1.18 | -0.33 | 0.24 | -3.28** | -4.82*** | 0.71 | 0.12 | -0.01 | 3.78 | 1005.85 |
|  |  | Drink | 1.12 | -0.39 | 0.16 | -3.36*** | -4.87*** | 0.60 | 0.12 | -0.01 | 3.78 | 1005.80 |
|  |  | Heat exhaustion | 1.35 | 0.00 | 0.19 | -3.38*** | -5.09*** | 0.47 | 0.05 | 0.04*** | 3.75 | 1001.24 |
|  |  | Heat stroke | 1.17 | -0.26 | 0.16 | -3.34*** | -4.81*** | 0.85 | 0.11 | 0.02 | 3.75 | 1004.81 |
|  |  | Hot weather | 1.12 | -0.34 | 0.17 | -3.44*** | -4.95*** | 0.75 | 0.11 | 0.01 | 3.76 | 1005.35 |
|  |  | Park | 1.16 | -0.38 | 0.27 | -3.23*** | -4.80*** | 0.70 | 0.12 | -0.02 | 3.78 | 1005.78 |
|  |  | Pool | 1.22 | -0.45 | 0.10 | -3.89*** | -5.32*** | 0.48 | 0.08 | 0.03 | 3.76 | 1004.95 |
|  |  | Swim | 1.08 | -0.26 | 0.10 | -3.73*** | -5.10*** | 0.48 | 0.08 | 0.04 | 3.77 | 1004.12 |
|  |  | Tired | 1.18 | -0.37 | 0.17 | -3.50*** | -5.00*** | 0.64 | 0.12 | 0.01 | 3.77 | 1005.75 |
|  |  | water | 1.14 | -0.37 | 0.16 | -3.53*** | -4.91*** | 0.75 | 0.14* | -0.04 | 3.77 | 1005.36 |
| Leon | None | | 0.55 | 0.22 | 0.22 | -0.86 | -0.45 | -0.28 | - | - | 1.63 | 734.04 |
|  |  |  | 0.56 | 0.22 | 0.23 | -0.85 | -0.45 | -0.29 | -0.01 | - | 1.63 | 735.82 |
|  | Twitter | Heat | 0.55 | 0.22 | 0.22 | -0.85 | -0.45 | -0.29 | -0.01 | 0.01 | 1.63 | 737.81 |
|  |  | AC | 0.53 | 0.24 | 0.17 | -0.86 | -0.33 | -0.27 | -0.03 | 0.25* | 1.64 | 735.37 |
|  | Google Search | AC repair | 0.56 | 0.28 | 0.22 | -0.82 | -0.45 | -0.27 | -0.01 | 0.00 | 1.61 | 737.41 |
|  |  | Beer | 0.58 | 0.32 | 0.33 | -0.70 | -0.38 | -0.28 | -0.01 | -0.01 | 1.63 | 736.91 |
|  |  | Drink | 0.58 | 0.23 | 0.26 | -0.73 | -0.32 | -0.30 | -0.01 | -0.01 | 1.63 | 737.19 |
|  |  | Heat exhaustion | 0.67 | 0.22 | 0.22 | -0.86 | -0.37 | -0.14 | -0.01 | -0.02** | 1.62 | 735.00 |
|  |  | Heat stroke | 0.57 | 0.26 | 0.22 | -0.83 | -0.43 | -0.28 | -0.01 | -0.01 | 1.63 | 737.32 |
|  |  | Hot weather | 0.58 | 0.26 | 0.26 | -0.84 | -0.45 | -0.26 | -0.01 | 0.00 | 1.63 | 737.56 |
|  |  | Park | 0.56 | 0.21 | 0.21 | -0.87 | -0.43 | -0.30 | -0.01 | 0.00 | 1.63 | 737.70 |
|  |  | Pool | 0.56 | 0.22 | 0.23 | -0.87 | -0.46 | -0.29 | -0.01 | 0.00 | 1.63 | 737.80 |
|  |  | Swim | 0.56 | 0.24 | 0.24 | -0.84 | -0.44 | -0.27 | -0.01 | 0.00 | 1.63 | 737.78 |
|  |  | Tired | 0.56 | 0.23 | 0.23 | -0.85 | -0.45 | -0.28 | -0.01 | 0.00 | 1.63 | 737.79 |
|  |  | water | 0.55 | 0.24 | 0.25 | -0.82 | -0.44 | -0.29 | -0.01 | 0.00 | 1.63 | 737.67 |
| Miami-Dade | None | | 0.01 | -0.04 | -0.91 | -7.49*** | -7.20*** | 0.47 | - | - | 4.78 | 1080.79 |
|  |  |  | 0.05 | -0.15 | -1.06 | -7.79*** | -7.18*** | 0.58 | 0.36*** | - | 4.68 | 1077.08 |
|  | Twitter | Heat | 0.06 | -0.14 | -1.03 | -7.79*** | -7.18*** | 0.59 | 0.36*** | 0.00 | 4.68 | 1079.06 |
|  |  | AC | 0.05 | -0.15 | -1.06 | -7.80*** | -7.18*** | 0.58 | 0.36*** | 0.00 | 4.68 | 1079.08 |
|  | Google Search | AC repair | 0.06 | -0.13 | -1.04 | -7.75*** | -7.09*** | 0.59 | 0.35*** | 0.01 | 4.69 | 1078.96 |
|  |  | Beer | 0.08 | -0.11 | -0.78 | -7.26*** | -6.72*** | 0.58 | 0.38*** | -0.03 | 4.70 | 1078.58 |
|  |  | Drink | 0.23 | 0.01 | -0.81 | -7.30*** | -6.69*** | 0.66 | 0.36*** | -0.03 | 4.66 | 1078.63 |
|  |  | Heat exhaustion | 0.11 | -0.11 | -1.06 | -7.78*** | -7.07*** | 0.64 | 0.38*** | -0.02 | 4.67 | 1078.50 |
|  |  | Heat stroke | 0.05 | -0.15 | -1.07 | -7.80*** | -7.17*** | 0.59 | 0.36*** | 0.00 | 4.68 | 1079.07 |
|  |  | Hot weather | 0.06 | -0.16 | -1.07 | -7.81*** | -7.21*** | 0.55 | 0.36*** | 0.00 | 4.68 | 1079.02 |
|  |  | Park | 0.10 | -0.08 | -0.76 | -7.21*** | -6.71*** | 0.78 | 0.39*** | -0.04 | 4.67 | 1078.57 |
|  |  | Pool | 0.06 | -0.15 | -1.06 | -7.82*** | -7.23*** | 0.56 | 0.35*** | 0.00 | 4.68 | 1079.07 |
|  |  | Swim | -0.02 | -0.18 | -0.94 | -7.82*** | -7.14*** | 0.45 | 0.29** | 0.04* | 4.62 | 1076.48 |
|  |  | Tired | 0.06 | -0.17 | -1.09 | -7.78*** | -7.18*** | 0.55 | 0.36*** | 0.00 | 4.67 | 1079.06 |
|  |  | water | 0.03 | -0.13 | -1.09 | -7.83*** | -7.27*** | 0.50 | 0.33** | 0.02 | 4.67 | 1078.95 |
| Orange | None | | -1.51 | -1.39 | -1.97* | -5.39*** | -5.72*** | -1.55 | - | - | 3.28 | 956.34 |
|  |  |  | -1.49 | -1.37 | -1.95* | -5.38*** | -5.72*** | -1.55 | -0.02 | - | 3.28 | 958.29 |
|  | Twitter | Heat | -1.49 | -1.37 | -1.95* | -5.38*** | -5.72*** | -1.55 | -0.02 | 0.00 | 3.28 | 960.28 |
|  |  | AC | -1.55 | -1.46 | -2.05** | -5.45*** | -5.79*** | -1.62 | 0.00 | -0.06 | 3.26 | 960.09 |
|  | Google Search | AC repair | -1.49 | -1.40 | -1.90* | -5.37*** | -5.69*** | -1.54 | -0.03 | 0.00 | 3.27 | 960.22 |
|  |  | Beer | -1.43 | -1.27 | -1.27 | -3.36*** | -3.91*** | -1.62 | 0.03 | -0.07*** | 3.27 | 955.03 |
|  |  | Drink | -1.16 | -0.99 | -1.37 | -3.40*** | -4.35*** | -1.50 | 0.08 | -0.09*** | 3.23 | 952.16 |
|  |  | Heat exhaustion | -1.44 | -1.26 | -1.72 | -5.19*** | -5.63*** | -1.21 | -0.06 | 0.03*** | 3.17 | 956.26 |
|  |  | Heat stroke | -1.59 | -1.54 | -1.94* | -5.40*** | -5.85*** | -1.58 | -0.05 | 0.02 | 3.26 | 958.60 |
|  |  | Hot weather | -1.42 | -1.48 | -2.01** | -5.16*** | -5.69*** | -1.61 | 0.00 | -0.02** | 3.28 | 957.24 |
|  |  | Park | -1.51 | -1.24 | -1.55 | -4.34*** | -5.30*** | -1.40 | 0.02 | -0.07* | 3.26 | 957.64 |
|  |  | Pool | -1.49 | -1.37 | -1.95* | -5.38*** | -5.73*** | -1.55 | -0.02 | 0.00 | 3.28 | 960.29 |
|  |  | Swim | -1.57 | -1.47 | -2.03** | -5.26*** | -5.63*** | -1.57 | 0.03 | -0.03 | 3.28 | 958.44 |
|  |  | Tired | -1.49 | -1.38 | -1.96* | -5.39*** | -5.73*** | -1.56 | -0.02 | 0.00 | 3.28 | 960.28 |
|  |  | water | -1.55 | -1.44 | -2.00* | -5.31*** | -5.52*** | -1.34 | 0.03 | -0.04 | 3.27 | 959.38 |

Table S9. Dehydration ED model specifications (*** <0.05, ** 0.05 ~ 0.10, * 0.10 ~ 0.15)

|  |  |  | Lag 0 | | | Lag 1 | | | Lag 2 | | | Lag 3 | | |
| --- | --- | --- | --- | --- | --- | --- | --- | --- | --- | --- | --- | --- | --- | --- |
|  | Keywords | | keyword | MAE | AIC | keyword | MAE | AIC | keyword | MAE | AIC | keyword | MAE | AIC |
| Duval | Twitter | Heat | 0.02*** | 3.47 | 971.72 | 0.01 | 3.50 | 977.63 | 0.02*** | 3.44 | 972.51 | 0.01 | 3.45 | 977.20 |
|  |  | AC | 0.04*** | 3.43 | 973.98 | -0.01 | 3.52 | 978.95 | 0.02 | 3.50 | 978.01 | 0.03* | 3.49 | 977.04 |
|  | Google Search | AC repair | 0.00 | 3.51 | 978.76 | 0.00** | 3.46 | 976.08 | 0.00* | 3.50 | 976.88 | 0.00 | 3.51 | 979.05 |
|  |  | Beer | 0.00 | 3.51 | 978.67 | 0.00 | 3.51 | 978.77 | 0.00 | 3.51 | 978.88 | 0.00 | 3.49 | 978.74 |
|  |  | Drink | 0.00 | 3.50 | 978.72 | 0.00 | 3.49 | 978.48 | 0.00 | 3.50 | 978.50 | 0.00 | 3.49 | 978.35 |
|  |  | Heat exhaustion | 0.00 | 3.47 | 977.29 | 0.00 | 3.51 | 979.03 | 0.00*** | 3.46 | 974.86 | 0.00 | 3.49 | 978.63 |
|  |  | Heat stroke | 0.00*** | 3.37 | 967.12 | 0.00 | 3.48 | 977.41 | 0.00 | 3.51 | 979.02 | 0.00*** | 3.39 | 973.96 |
|  |  | Hot weather | 0.00 | 3.51 | 978.96 | 0.00 | 3.51 | 978.95 | 0.00* | 3.48 | 977.04 | 0.00 | 3.50 | 977.90 |
|  |  | Park | 0.00*** | 3.44 | 975.10 | 0.00 | 3.51 | 978.91 | 0.00 | 3.51 | 978.77 | 0.00 | 3.50 | 978.89 |
|  |  | Pool | 0.00*** | 3.40 | 973.45 | 0.00 | 3.50 | 978.09 | 0.00 | 3.52 | 978.95 | 0.00 | 3.50 | 978.31 |
|  |  | Swim | 0.00** | 3.47 | 975.82 | 0.00 | 3.51 | 978.92 | 0.00 | 3.51 | 978.93 | 0.00*** | 3.44 | 974.70 |
|  |  | Tired | 0.00 | 3.50 | 977.33 | 0.00 | 3.51 | 978.92 | 0.00 | 3.50 | 978.43 | 0.00 | 3.51 | 978.59 |
|  |  | water | 0.00 | 3.50 | 978.50 | 0.00 | 3.51 | 979.05 | 0.00 | 3.51 | 979.05 | 0.00 | 3.52 | 978.96 |
| Hillsborough | Twitter | Heat | 0.01* | 3.47 | 990.05 | 0.01* | 3.45 | 990.01 | 0.00 | 3.49 | 992.18 | 0.01 | 3.49 | 991.39 |
|  |  | AC | 0.02*** | 3.40 | 988.18 | 0.01 | 3.49 | 991.81 | 0.00 | 3.49 | 992.44 | 0.01 | 3.48 | 990.92 |
|  | Google Search | AC repair | 0.00 | 3.50 | 992.38 | 0.00 | 3.49 | 991.78 | 0.00 | 3.49 | 991.59 | 0.00 | 3.50 | 991.86 |
|  |  | Beer | 0.00 | 3.48 | 992.10 | 0.00 | 3.50 | 992.40 | 0.00 | 3.50 | 992.16 | 0.00 | 3.51 | 992.15 |
|  |  | Drink | 0.00 | 3.50 | 992.50 | 0.00 | 3.44 | 990.75 | 0.00** | 3.47 | 989.85 | 0.00 | 3.50 | 991.61 |
|  |  | Heat exhaustion | 0.00 | 3.49 | 990.90 | 0.00 | 3.50 | 992.30 | 0.00 | 3.49 | 991.49 | 0.00 | 3.45 | 991.13 |
|  |  | Heat stroke | 0.00 | 3.49 | 990.66 | 0.00 | 3.50 | 992.52 | 0.00 | 3.48 | 992.28 | 0.00*** | 3.49 | 988.76 |
|  |  | Hot weather | 0.00*** | 3.51 | 988.65 | 0.00*** | 3.49 | 986.89 | 0.00 | 3.46 | 991.12 | 0.00 | 3.48 | 991.91 |
|  |  | Park | 0.01*** | 3.49 | 985.47 | 0.00* | 3.50 | 990.52 | 0.00 | 3.49 | 990.76 | 0.00 | 3.50 | 992.33 |
|  |  | Pool | 0.01*** | 3.43 | 982.04 | 0.00*** | 3.50 | 988.39 | 0.00*** | 3.49 | 986.99 | 0.00 | 3.49 | 990.62 |
|  |  | Swim | 0.00*** | 3.44 | 985.04 | 0.00*** | 3.45 | 987.91 | 0.01*** | 3.33 | 975.64 | 0.01*** | 3.44 | 979.90 |
|  |  | Tired | 0.00 | 3.51 | 991.79 | 0.00 | 3.52 | 990.81 | 0.00 | 3.50 | 991.92 | 0.00 | 3.49 | 992.51 |
|  |  | water | 0.01*** | 3.42 | 983.49 | 0.01*** | 3.46 | 986.72 | 0.00** | 3.49 | 989.39 | 0.01*** | 3.44 | 986.33 |
| Leon | Twitter | Heat | 0.00 | 2.00 | 795.64 | 0.01 | 2.00 | 795.64 | -0.03 | 2.01 | 795.40 | -0.06 | 2.01 | 794.71 |
|  |  | AC | 0.02 | 2.00 | 795.64 | -0.24 | 2.01 | 794.16 | 0.00 | 2.00 | 795.65 | -0.15 | 2.01 | 794.99 |
|  | Google Search | AC repair | 0.00 | 2.00 | 795.44 | 0.00 | 2.01 | 795.50 | 0.00 | 2.00 | 795.38 | -0.01 | 2.00 | 794.47 |
|  |  | Beer | 0.02* | 1.98 | 793.16 | 0.01 | 2.00 | 794.12 | 0.01 | 2.00 | 794.49 | 0.01 | 2.00 | 794.10 |
|  |  | Drink | -0.01 | 2.01 | 795.01 | -0.03*** | 1.97 | 791.40 | -0.01 | 2.00 | 795.06 | 0.01 | 2.00 | 795.23 |
|  |  | Heat exhaustion | 0.00 | 2.00 | 795.60 | 0.01 | 2.00 | 795.48 | 0.02 | 1.98 | 793.59 | 0.03** | 1.99 | 792.43 |
|  |  | Heat stroke | 0.00 | 2.00 | 795.65 | 0.02* | 1.98 | 793.43 | 0.00 | 2.00 | 795.63 | 0.01 | 1.98 | 794.87 |
|  |  | Hot weather | -0.02*** | 1.96 | 791.19 | 0.01 | 2.00 | 795.16 | 0.01 | 1.99 | 795.41 | 0.01 | 1.99 | 794.04 |
|  |  | Park | -0.02 | 2.00 | 794.54 | 0.00 | 2.00 | 795.63 | 0.00 | 2.00 | 795.65 | 0.01 | 2.01 | 795.50 |
|  |  | Pool | 0.01 | 2.01 | 795.08 | -0.01 | 2.00 | 795.38 | 0.00 | 2.00 | 795.64 | -0.01 | 1.99 | 794.71 |
|  |  | Swim | 0.00 | 2.01 | 795.54 | 0.01 | 2.01 | 794.24 | 0.00 | 2.00 | 795.65 | 0.01 | 2.00 | 794.82 |
|  |  | Tired | 0.00 | 2.01 | 795.43 | 0.00 | 2.00 | 795.59 | 0.02*** | 1.98 | 791.71 | 0.02** | 2.00 | 792.55 |
|  |  | water | 0.02* | 2.00 | 792.94 | 0.01 | 2.01 | 795.21 | -0.03*** | 1.97 | 791.74 | 0.00 | 2.00 | 795.63 |
| Miami-Dade | Twitter | Heat | 0.00 | 4.39 | 1052.89 | 0.00 | 4.36 | 1053.07 | 0.00** | 4.34 | 1051.04 | 0.00 | 4.39 | 1053.57 |
|  |  | AC | 0.00 | 4.37 | 1053.33 | 0.00 | 4.40 | 1053.02 | 0.00 | 4.37 | 1052.38 | 0.01*** | 4.34 | 1049.21 |
|  | Google Search | AC repair | 0.00 | 4.42 | 1052.39 | 0.00 | 4.36 | 1051.77 | 0.00 | 4.38 | 1053.60 | 0.00*** | 4.25 | 1046.13 |
|  |  | Beer | 0.00** | 4.37 | 1050.81 | 0.00*** | 4.33 | 1048.87 | 0.00* | 4.38 | 1051.38 | 0.00* | 4.32 | 1051.57 |
|  |  | Drink | 0.00 | 4.38 | 1053.23 | 0.00 | 4.39 | 1053.74 | 0.00 | 4.37 | 1052.82 | 0.00 | 4.37 | 1051.87 |
|  |  | Heat exhaustion | 0.00 | 4.41 | 1053.24 | 0.00 | 4.36 | 1051.79 | 0.00 | 4.35 | 1052.89 | 0.00 | 4.38 | 1052.26 |
|  |  | Heat stroke | 0.00 | 4.39 | 1053.34 | 0.00 | 4.38 | 1053.47 | 0.00 | 4.38 | 1053.74 | 0.00 | 4.38 | 1053.18 |
|  |  | Hot weather | 0.00 | 4.38 | 1053.55 | 0.00 | 4.39 | 1053.74 | 0.00 | 4.37 | 1052.90 | 0.00* | 4.36 | 1051.33 |
|  |  | Park | 0.01*** | 4.28 | 1045.89 | 0.00*** | 4.30 | 1048.69 | 0.01*** | 4.22 | 1044.66 | 0.00*** | 4.29 | 1048.95 |
|  |  | Pool | 0.00*** | 4.25 | 1046.55 | 0.00*** | 4.29 | 1048.09 | 0.00*** | 4.26 | 1046.01 | 0.00*** | 4.25 | 1042.84 |
|  |  | Swim | 0.00* | 4.32 | 1051.52 | 0.00*** | 4.26 | 1049.29 | 0.00*** | 4.27 | 1045.94 | 0.00*** | 4.31 | 1049.94 |
|  |  | Tired | 0.00 | 4.37 | 1053.42 | 0.00 | 4.37 | 1053.04 | 0.00 | 4.38 | 1053.73 | 0.00 | 4.37 | 1053.46 |
|  |  | Water | 0.01*** | 4.27 | 1047.30 | 0.01*** | 4.25 | 1046.29 | 0.00*** | 4.28 | 1049.12 | 0.00*** | 4.30 | 1049.30 |
| Orange | Twitter | Heat | -0.03 | 2.93 | 920.15 | -0.04 | 2.93 | 919.89 | 0.01 | 2.93 | 920.52 | 0.07** | 2.86 | 917.74 |
|  |  | AC | 0.00 | 2.93 | 920.58 | -0.04 | 2.93 | 920.43 | 0.09 | 2.93 | 919.90 | 0.03 | 2.93 | 920.52 |
|  | Google Search | AC repair | 0.04*** | 2.85 | 915.21 | 0.00 | 2.93 | 920.56 | -0.01 | 2.92 | 920.25 | 0.01 | 2.92 | 920.39 |
|  |  | Beer | -0.02 | 2.95 | 920.13 | 0.01 | 2.92 | 920.32 | -0.02 | 2.92 | 920.15 | -0.03 | 2.93 | 919.78 |
|  |  | Drink | -0.02 | 2.94 | 920.01 | -0.01 | 2.95 | 920.43 | 0.00 | 2.93 | 920.57 | 0.05** | 2.89 | 917.34 |
|  |  | Heat exhaustion | -0.01 | 2.93 | 920.04 | 0.01 | 2.92 | 920.16 | 0.00 | 2.93 | 920.58 | 0.00 | 2.93 | 920.58 |
|  |  | Heat stroke | 0.02** | 2.88 | 917.79 | 0.01 | 2.92 | 920.14 | 0.03** | 2.86 | 917.47 | 0.01 | 2.92 | 920.31 |
|  |  | Hot weather | -0.01 | 2.92 | 920.01 | 0.00 | 2.93 | 920.58 | 0.00 | 2.93 | 920.43 | -0.01 | 2.93 | 920.15 |
|  |  | Park | -0.03 | 2.94 | 919.80 | -0.01 | 2.94 | 920.50 | -0.03 | 2.94 | 919.83 | 0.02 | 2.92 | 920.36 |
|  |  | Pool | 0.00 | 2.93 | 920.55 | 0.00 | 2.93 | 920.57 | 0.01 | 2.93 | 920.52 | 0.02 | 2.91 | 919.88 |
|  |  | Swim | 0.00 | 2.93 | 920.55 | 0.01 | 2.92 | 920.34 | -0.01 | 2.93 | 920.18 | -0.01 | 2.94 | 920.24 |
|  |  | Tired | -0.02 | 2.93 | 919.66 | 0.02 | 2.88 | 918.46 | -0.03* | 2.94 | 918.21 | 0.03* | 2.91 | 918.29 |
|  |  | water | -0.03 | 2.96 | 919.92 | -0.01 | 2.93 | 920.44 | 0.04 | 2.90 | 919.46 | 0.01 | 2.93 | 920.45 |

Table S10. Dehydration hospitalization model specifications (*** <0.05, ** 0.05 ~ 0.10, * 0.10 ~ 0.15)

|  |  |  | Lag 0 | | | Lag 1 | | | Lag 2 | | | Lag 3 | | |
| --- | --- | --- | --- | --- | --- | --- | --- | --- | --- | --- | --- | --- | --- | --- |
|  | Keywords | | keyword | MAE | AIC | keyword | MAE | AIC | keyword | MAE | AIC | keyword | MAE | AIC |
| Duval | Twitter | Heat | 0.24** | 3.79 | 1021.79 | 0.20 | 3.84 | 1022.76 | -0.06 | 3.85 | 1024.48 | -0.06 | 3.86 | 1024.52 |
|  |  | AC | -0.05 | 3.86 | 1024.67 | 0.60** | 3.83 | 1021.26 | 0.56** | 3.89 | 1021.67 | 0.35 | 3.87 | 1023.48 |
|  | Google Search | AC repair | 0.00 | 3.86 | 1024.68 | -0.02 | 3.84 | 1023.75 | -0.03* | 3.84 | 1022.05 | 0.00 | 3.87 | 1024.64 |
|  |  | Beer | -0.01 | 3.85 | 1024.57 | -0.03 | 3.87 | 1023.18 | -0.03 | 3.84 | 1023.40 | 0.00 | 3.86 | 1024.69 |
|  |  | Drink | 0.00 | 3.86 | 1024.69 | 0.00 | 3.86 | 1024.69 | -0.01 | 3.89 | 1024.37 | -0.03 | 3.84 | 1023.33 |
|  |  | Heat exhaustion | 0.00 | 3.87 | 1024.63 | 0.00 | 3.86 | 1024.62 | 0.03** | 3.80 | 1021.38 | 0.03* | 3.85 | 1022.01 |
|  |  | Heat stroke | -0.02 | 3.86 | 1023.95 | -0.01 | 3.87 | 1024.56 | -0.02 | 3.87 | 1024.06 | -0.05** | 3.88 | 1021.07 |
|  |  | Hot weather | -0.01 | 3.86 | 1024.31 | -0.02 | 3.85 | 1024.00 | -0.05*** | 3.85 | 1017.48 | 0.01 | 3.85 | 1024.48 |
|  |  | Park | -0.02 | 3.87 | 1024.35 | -0.04 | 3.86 | 1023.67 | 0.00 | 3.86 | 1024.69 | -0.07** | 3.85 | 1021.60 |
|  |  | Pool | -0.02 | 3.86 | 1024.07 | -0.02 | 3.87 | 1023.80 | 0.00 | 3.86 | 1024.69 | -0.02 | 3.86 | 1024.22 |
|  |  | Swim | -0.02 | 3.87 | 1023.57 | 0.00 | 3.86 | 1024.66 | -0.01 | 3.87 | 1024.55 | 0.00 | 3.86 | 1024.64 |
|  |  | Tired | -0.03 | 3.86 | 1022.90 | 0.01 | 3.87 | 1024.46 | 0.02 | 3.83 | 1023.21 | -0.01 | 3.87 | 1024.19 |
|  |  | water | -0.05 | 3.83 | 1022.56 | -0.11*** | 3.80 | 1015.44 | -0.05 | 3.89 | 1023.08 | -0.04 | 3.86 | 1023.73 |
| Hillsborough | Twitter | Heat | 0.13 | 3.72 | 1004.09 | 0.06 | 3.76 | 1005.53 | -0.06 | 3.78 | 1005.51 | -0.12 | 3.74 | 1004.37 |
|  |  | AC | 0.49*** | 3.65 | 999.36 | 0.02 | 3.78 | 1005.88 | 0.06 | 3.78 | 1005.79 | -0.05 | 3.77 | 1005.83 |
|  | Google Search | AC repair | -0.04 | 3.77 | 1003.90 | -0.01 | 3.78 | 1005.81 | 0.01 | 3.78 | 1005.78 | -0.03 | 3.76 | 1004.17 |
|  |  | Beer | -0.01 | 3.78 | 1005.85 | 0.01 | 3.77 | 1005.75 | 0.03 | 3.78 | 1005.10 | 0.00 | 3.78 | 1005.88 |
|  |  | Drink | -0.01 | 3.78 | 1005.80 | 0.02 | 3.75 | 1005.63 | 0.04 | 3.73 | 1003.96 | -0.05* | 3.74 | 1003.53 |
|  |  | Heat exhaustion | 0.04*** | 3.75 | 1001.24 | -0.01 | 3.79 | 1005.80 | 0.01 | 3.79 | 1005.63 | 0.02 | 3.77 | 1004.96 |
|  |  | Heat stroke | 0.02 | 3.75 | 1004.81 | 0.01 | 3.78 | 1005.14 | -0.02 | 3.73 | 1003.86 | -0.02 | 3.75 | 1004.90 |
|  |  | Hot weather | 0.01 | 3.76 | 1005.35 | 0.00 | 3.78 | 1005.87 | -0.01 | 3.77 | 1005.61 | 0.01 | 3.78 | 1005.82 |
|  |  | Park | -0.02 | 3.78 | 1005.78 | -0.01 | 3.77 | 1005.84 | 0.03 | 3.79 | 1005.59 | 0.01 | 3.78 | 1005.85 |
|  |  | Pool | 0.03 | 3.76 | 1004.95 | 0.00 | 3.78 | 1005.89 | 0.05** | 3.76 | 1002.89 | 0.05** | 3.77 | 1002.44 |
|  |  | Swim | 0.04 | 3.77 | 1004.12 | 0.04 | 3.78 | 1004.42 | 0.03 | 3.77 | 1004.74 | 0.06** | 3.74 | 1002.63 |
|  |  | Tired | 0.01 | 3.77 | 1005.75 | 0.02 | 3.75 | 1004.52 | 0.00 | 3.78 | 1005.89 | 0.00 | 3.77 | 1005.86 |
|  |  | water | -0.04 | 3.77 | 1005.36 | 0.11*** | 3.74 | 1001.21 | -0.01 | 3.78 | 1005.83 | 0.11*** | 3.73 | 1000.76 |
| Leon | Twitter | Heat | 0.01 | 1.63 | 737.81 | -0.02 | 1.63 | 737.73 | 0.07 | 1.62 | 736.17 | -0.12*** | 1.60 | 733.44 |
|  |  | AC | 0.25* | 1.64 | 735.37 | -0.06 | 1.63 | 737.70 | -0.04 | 1.63 | 737.76 | -0.40*** | 1.61 | 730.83 |
|  | Google Search | AC repair | 0.00 | 1.61 | 737.41 | 0.00 | 1.63 | 737.80 | 0.00 | 1.63 | 737.82 | 0.01 | 1.63 | 737.15 |
|  |  | Beer | -0.01 | 1.63 | 736.91 | -0.01 | 1.63 | 737.53 | -0.01 | 1.61 | 735.97 | 0.01 | 1.64 | 737.41 |
|  |  | Drink | -0.01 | 1.63 | 737.19 | -0.01 | 1.62 | 737.59 | -0.01 | 1.63 | 737.19 | 0.00 | 1.63 | 737.82 |
|  |  | Heat exhaustion | -0.02** | 1.62 | 735.00 | -0.02 | 1.62 | 736.17 | 0.01 | 1.63 | 737.57 | -0.02 | 1.62 | 736.46 |
|  |  | Heat stroke | -0.01 | 1.63 | 737.32 | -0.01 | 1.62 | 737.18 | -0.01 | 1.62 | 737.34 | -0.01 | 1.62 | 737.33 |
|  |  | Hot weather | 0.00 | 1.63 | 737.56 | 0.00 | 1.63 | 737.70 | 0.01 | 1.63 | 737.06 | -0.01 | 1.64 | 736.52 |
|  |  | Park | 0.00 | 1.63 | 737.70 | 0.00 | 1.63 | 737.78 | 0.01 | 1.62 | 737.42 | -0.01 | 1.63 | 736.80 |
|  |  | Pool | 0.00 | 1.63 | 737.80 | 0.00 | 1.63 | 737.76 | 0.01 | 1.62 | 737.24 | -0.01 | 1.61 | 735.72 |
|  |  | Swim | 0.00 | 1.63 | 737.78 | -0.01 | 1.62 | 737.36 | -0.01 | 1.62 | 735.89 | 0.00 | 1.63 | 737.81 |
|  |  | Tired | 0.00 | 1.63 | 737.79 | 0.00 | 1.63 | 737.72 | 0.02** | 1.61 | 734.43 | 0.00 | 1.63 | 737.82 |
|  |  | water | 0.00 | 1.63 | 737.67 | 0.01 | 1.63 | 736.55 | 0.00 | 1.63 | 737.72 | 0.00 | 1.63 | 737.82 |
| Miami-Dade | Twitter | Heat | 0.00 | 4.68 | 1079.06 | 0.00 | 4.68 | 1079.07 | 0.02 | 4.67 | 1078.60 | 0.01 | 4.67 | 1078.74 |
|  |  | AC | 0.00 | 4.68 | 1079.08 | 0.23*** | 4.54 | 1073.54 | 0.07 | 4.66 | 1078.63 | 0.10 | 4.67 | 1077.94 |
|  | Google Search | AC repair | 0.01 | 4.69 | 1078.96 | 0.01 | 4.67 | 1078.87 | -0.02 | 4.66 | 1078.24 | 0.00 | 4.68 | 1079.05 |
|  |  | Beer | -0.03 | 4.70 | 1078.58 | 0.04 | 4.64 | 1077.91 | 0.07** | 4.58 | 1075.93 | -0.03 | 4.66 | 1078.41 |
|  |  | Drink | -0.03 | 4.66 | 1078.63 | 0.09*** | 4.59 | 1074.11 | 0.00 | 4.68 | 1079.07 | -0.07* | 4.63 | 1076.32 |
|  |  | Heat exhaustion | -0.02 | 4.67 | 1078.50 | -0.01 | 4.66 | 1078.75 | -0.05*** | 4.59 | 1073.12 | 0.05*** | 4.61 | 1073.99 |
|  |  | Heat stroke | 0.00 | 4.68 | 1079.07 | 0.02 | 4.62 | 1077.91 | 0.02 | 4.68 | 1078.34 | -0.01 | 4.66 | 1078.65 |
|  |  | Hot weather | 0.00 | 4.68 | 1079.02 | -0.03* | 4.68 | 1076.57 | -0.02 | 4.68 | 1077.48 | -0.04*** | 4.62 | 1073.32 |
|  |  | Park | -0.04 | 4.67 | 1078.57 | 0.05 | 4.62 | 1078.16 | 0.08* | 4.61 | 1076.76 | 0.05 | 4.63 | 1078.13 |
|  |  | Pool | 0.00 | 4.68 | 1079.07 | 0.05 | 4.59 | 1077.41 | 0.03 | 4.65 | 1078.52 | -0.02 | 4.69 | 1078.89 |
|  |  | Swim | 0.04* | 4.62 | 1076.48 | 0.00 | 4.68 | 1079.07 | 0.00 | 4.68 | 1079.06 | 0.04* | 4.60 | 1076.39 |
|  |  | Tired | 0.00 | 4.67 | 1079.06 | -0.02 | 4.67 | 1078.80 | 0.05 | 4.66 | 1077.22 | 0.02 | 4.67 | 1078.61 |
|  |  | Water | 0.02 | 4.67 | 1078.95 | 0.06 | 4.61 | 1077.92 | 0.04 | 4.67 | 1078.70 | -0.07 | 4.70 | 1077.84 |
| Orange | Twitter | Heat | 0.00 | 3.28 | 960.28 | 0.02 | 3.27 | 960.13 | -0.05 | 3.26 | 959.30 | 0.01 | 3.27 | 960.26 |
|  |  | AC | -0.06 | 3.26 | 960.09 | 0.14 | 3.27 | 959.15 | 0.00 | 3.28 | 960.29 | -0.01 | 3.28 | 960.28 |
|  | Google Search | AC repair | 0.00 | 3.27 | 960.22 | 0.05*** | 3.25 | 953.81 | 0.02 | 3.26 | 959.33 | -0.01 | 3.27 | 959.94 |
|  |  | Beer | -0.07*** | 3.27 | 955.03 | -0.05 | 3.24 | 958.28 | 0.00 | 3.28 | 960.27 | -0.01 | 3.27 | 960.11 |
|  |  | Drink | -0.09*** | 3.23 | 952.16 | 0.04 | 3.25 | 958.32 | -0.01 | 3.27 | 960.21 | 0.00 | 3.27 | 960.28 |
|  |  | Heat exhaustion | 0.03*** | 3.17 | 956.26 | -0.01 | 3.28 | 959.78 | -0.02* | 3.27 | 957.98 | -0.03*** | 3.28 | 956.26 |
|  |  | Heat stroke | 0.02 | 3.26 | 958.60 | -0.01 | 3.27 | 959.80 | 0.03** | 3.22 | 956.51 | -0.01 | 3.28 | 960.08 |
|  |  | Hot weather | -0.02** | 3.28 | 957.24 | 0.00 | 3.27 | 960.28 | 0.02 | 3.27 | 958.80 | 0.02 | 3.25 | 958.80 |
|  |  | Park | -0.07* | 3.26 | 957.64 | -0.05 | 3.27 | 958.69 | -0.01 | 3.28 | 960.24 | -0.03 | 3.27 | 959.62 |
|  |  | Pool | 0.00 | 3.28 | 960.29 | -0.01 | 3.27 | 960.07 | -0.03 | 3.26 | 958.85 | -0.03 | 3.26 | 959.11 |
|  |  | Swim | -0.03 | 3.28 | 958.44 | -0.01 | 3.26 | 959.88 | -0.01 | 3.27 | 959.84 | 0.00 | 3.28 | 960.29 |
|  |  | Tired | 0.00 | 3.28 | 960.28 | -0.01 | 3.27 | 960.06 | 0.02 | 3.27 | 959.33 | 0.02 | 3.26 | 959.58 |
|  |  | water | -0.04 | 3.27 | 959.38 | -0.04 | 3.28 | 959.50 | 0.00 | 3.27 | 960.28 | -0.06 | 3.25 | 958.17 |

Table S11. Renal illness ED model specifications (*** <0.05, ** 0.05 ~ 0.10, * 0.10 ~ 0.15)

| County | Keywords | | Wed | Thu | Fri | Sat | Sun | Mon | Max T | keyword | MAE | AIC |
| --- | --- | --- | --- | --- | --- | --- | --- | --- | --- | --- | --- | --- |
| Duval | None | | -0.13 | -0.65** | -1.34*** | -0.96*** | -1.29*** | -0.67** | - | - | 0.91 | 537.71 |
|  |  |  | -0.15 | -0.68*** | -1.36*** | -0.98*** | -1.29*** | -0.66** | 0.03** | - | 0.89 | 536.83 |
|  | Twitter | Heat | -0.15 | -0.68** | -1.36*** | -0.98*** | -1.28*** | -0.66** | 0.02 | 0.01 | 0.89 | 538.72 |
|  |  | AC | -0.13 | -0.69*** | -1.34*** | -0.96*** | -1.27*** | -0.66** | 0.03** | -0.06 | 0.89 | 538.16 |
|  | Google Search | AC repair | -0.15 | -0.68** | -1.36*** | -0.97*** | -1.28*** | -0.65** | 0.03* | 0.00 | 0.89 | 538.81 |
|  |  | Beer | -0.15 | -0.70*** | -1.33*** | -0.88*** | -1.21*** | -0.66** | 0.03** | -0.01 | 0.89 | 538.07 |
|  |  | Drink | -0.16 | -0.68*** | -1.37*** | -1.02*** | -1.34*** | -0.65** | 0.02* | 0.00 | 0.89 | 538.38 |
|  |  | Heat exhaustion | -0.17 | -0.68*** | -1.36*** | -0.99*** | -1.29*** | -0.66** | 0.03** | 0.00 | 0.89 | 538.64 |
|  |  | Heat stroke | -0.11 | -0.67*** | -1.38*** | -0.90*** | -1.34*** | -0.70*** | 0.02 | 0.01*** | 0.89 | 534.18 |
|  |  | Hot weather | -0.15 | -0.68** | -1.36*** | -0.98*** | -1.29*** | -0.66** | 0.03** | 0.00 | 0.89 | 538.83 |
|  |  | Park | -0.16 | -0.67** | -1.47*** | -1.15*** | -1.35*** | -0.72*** | 0.01 | 0.02** | 0.89 | 535.86 |
|  |  | Pool | -0.10 | -0.70*** | -1.38*** | -1.17*** | -1.45*** | -0.69*** | 0.01 | 0.02*** | 0.87 | 530.94 |
|  |  | Swim | -0.10 | -0.67** | -1.27*** | -0.99*** | -1.32*** | -0.63** | 0.02 | 0.01** | 0.89 | 534.95 |
|  |  | Tired | -0.13 | -0.65** | -1.36*** | -0.96*** | -1.27*** | -0.65** | 0.03** | 0.00 | 0.89 | 538.64 |
|  |  | water | -0.20 | -0.73*** | -1.36*** | -1.03*** | -1.35*** | -0.70*** | 0.02 | 0.01* | 0.88 | 536.64 |
| Hillsborough | None | | -0.07 | 0.13 | 0.12 | -0.01 | -0.10 | 0.09 | - | - | 1.05 | 544.46 |
|  |  |  | -0.08 | 0.12 | 0.12 | -0.01 | -0.10 | 0.11 | 0.02 | - | 1.05 | 545.02 |
|  | Twitter | Heat | -0.08 | 0.13 | 0.13 | -0.01 | -0.10 | 0.11 | 0.02 | -0.01 | 1.05 | 546.79 |
|  |  | AC | -0.07 | 0.12 | 0.12 | -0.04 | -0.12 | 0.12 | 0.03* | -0.05 | 1.04 | 544.99 |
|  | Google Search | AC repair | -0.07 | 0.14 | 0.14 | 0.04 | -0.08 | 0.12 | 0.03 | 0.00 | 1.05 | 546.44 |
|  |  | Beer | -0.08 | 0.13 | 0.13 | 0.02 | -0.08 | 0.11 | 0.02 | 0.00 | 1.05 | 547.00 |
|  |  | Drink | -0.15 | 0.11 | 0.13 | 0.13 | 0.00 | -0.01 | 0.03* | -0.01*** | 1.02 | 542.16 |
|  |  | Heat exhaustion | -0.10 | 0.08 | 0.11 | -0.02 | -0.09 | 0.12 | 0.03* | 0.00 | 1.04 | 546.16 |
|  |  | Heat stroke | -0.08 | 0.11 | 0.12 | -0.02 | -0.11 | 0.09 | 0.02 | 0.00 | 1.05 | 546.82 |
|  |  | Hot weather | -0.08 | 0.12 | 0.12 | -0.01 | -0.10 | 0.10 | 0.02 | 0.00 | 1.05 | 546.89 |
|  |  | Park | -0.09 | 0.12 | 0.14 | 0.04 | -0.07 | 0.11 | 0.02 | 0.00 | 1.05 | 546.91 |
|  |  | Pool | -0.09 | 0.13 | 0.12 | 0.05 | -0.05 | 0.13 | 0.03 | 0.00 | 1.05 | 546.45 |
|  |  | Swim | -0.06 | 0.08 | 0.13 | 0.06 | -0.06 | 0.16 | 0.03** | -0.01** | 1.04 | 543.44 |
|  |  | Tired | -0.08 | 0.13 | 0.13 | -0.01 | -0.12 | 0.09 | 0.02 | 0.00 | 1.04 | 546.64 |
|  |  | water | -0.08 | 0.12 | 0.11 | 0.00 | -0.11 | 0.09 | 0.02 | 0.01 | 1.04 | 546.37 |
| Leon | None | | 0.09 | 0.00 | 0.03 | 0.15 | -0.20 | -0.20 | - | - | 0.67 | 358.38 |
|  |  |  | 0.08 | -0.01 | 0.01 | 0.12 | -0.20 | -0.20 | 0.02 | - | 0.66 | 358.73 |
|  | Twitter | Heat | 0.07 | -0.03 | -0.01 | 0.12 | -0.21 | -0.23 | 0.02 | 0.02 | 0.66 | 360.45 |
|  |  | AC | 0.10 | -0.01 | 0.03 | 0.13 | -0.24 | -0.20 | 0.03* | -0.08 | 0.66 | 360.04 |
|  | Google Search | AC repair | 0.07 | -0.11 | 0.03 | 0.05 | -0.21 | -0.23 | 0.02 | 0.01** | 0.64 | 357.54 |
|  |  | Beer | 0.09 | 0.04 | 0.06 | 0.20 | -0.17 | -0.20 | 0.02 | 0.00 | 0.66 | 360.20 |
|  |  | Drink | 0.16 | -0.02 | 0.11 | 0.43 | 0.08 | -0.21 | 0.03** | -0.03*** | 0.63 | 351.73 |
|  |  | Heat exhaustion | 0.09 | -0.01 | 0.01 | 0.12 | -0.20 | -0.18 | 0.02 | 0.00 | 0.66 | 360.67 |
|  |  | Heat stroke | 0.08 | 0.05 | 0.00 | 0.16 | -0.17 | -0.20 | 0.03* | -0.02** | 0.64 | 355.28 |
|  |  | Hot weather | 0.11 | 0.02 | 0.05 | 0.14 | -0.21 | -0.18 | 0.03 | 0.00 | 0.66 | 360.07 |
|  |  | Park | 0.08 | -0.01 | 0.01 | 0.12 | -0.20 | -0.20 | 0.02 | 0.00 | 0.66 | 360.73 |
|  |  | Pool | 0.08 | -0.02 | 0.00 | 0.17 | -0.17 | -0.20 | 0.03 | 0.00 | 0.66 | 360.17 |
|  |  | Swim | 0.09 | 0.02 | 0.04 | 0.15 | -0.18 | -0.17 | 0.03 | 0.00 | 0.66 | 360.13 |
|  |  | Tired | 0.08 | -0.01 | 0.02 | 0.12 | -0.20 | -0.19 | 0.02 | 0.00 | 0.66 | 360.69 |
|  |  | water | 0.09 | -0.04 | -0.02 | 0.09 | -0.21 | -0.19 | 0.03 | -0.01 | 0.66 | 360.09 |
| Miami-Dade | None | | -0.95 | 0.65 | 0.09 | -0.91 | -2.66*** | 0.92 | - | - | 2.02 | 796.91 |
|  |  |  | -0.93 | 0.61 | 0.03 | -1.03 | -2.65*** | 0.97 | 0.14*** | - | 1.96 | 793.88 |
|  | Twitter | Heat | -0.93 | 0.61 | 0.03 | -1.03 | -2.65*** | 0.97 | 0.14*** | 0.00 | 1.96 | 795.87 |
|  |  | AC | -0.95 | 0.70 | -0.04 | -0.89 | -2.55*** | 0.91 | 0.11** | 0.07** | 1.94 | 792.75 |
|  | Google Search | AC repair | -0.94 | 0.59 | 0.01 | -1.07 | -2.74*** | 0.96 | 0.16*** | -0.01 | 1.96 | 795.29 |
|  |  | Beer | -0.95 | 0.58 | -0.17 | -1.41** | -2.98*** | 0.97 | 0.13*** | 0.02 | 1.95 | 794.44 |
|  |  | Drink | -0.95 | 0.59 | 0.00 | -1.09 | -2.71*** | 0.96 | 0.14*** | 0.00 | 1.96 | 795.84 |
|  |  | Heat exhaustion | -0.97 | 0.58 | 0.03 | -1.03 | -2.72*** | 0.93 | 0.13** | 0.01 | 1.95 | 794.43 |
|  |  | Heat stroke | -0.97 | 0.58 | -0.01 | -1.07 | -2.62*** | 1.03 | 0.15*** | -0.01 | 1.95 | 795.13 |
|  |  | Hot weather | -0.94 | 0.62 | 0.04 | -1.01 | -2.62*** | 1.00 | 0.15*** | 0.00 | 1.97 | 795.60 |
|  |  | Park | -0.93 | 0.61 | 0.03 | -1.03 | -2.66*** | 0.97 | 0.14*** | 0.00 | 1.96 | 795.87 |
|  |  | Pool | -0.95 | 0.61 | 0.04 | -0.91 | -2.45*** | 1.03 | 0.17*** | -0.02 | 1.97 | 794.84 |
|  |  | Swim | -0.92 | 0.62 | 0.00 | -1.02 | -2.66*** | 1.00 | 0.16*** | -0.01 | 1.95 | 795.19 |
|  |  | Tired | -0.94 | 0.69 | 0.12 | -1.08* | -2.65*** | 1.06 | 0.13*** | 0.02 | 1.98 | 794.56 |
|  |  | water | -0.93 | 0.61 | 0.04 | -1.02 | -2.62*** | 0.99 | 0.15*** | -0.01 | 1.96 | 795.82 |
| Orange | None | | 0.10 | 0.52 | -0.27 | -0.02 | -0.23 | -0.44 | - | - | 1.17 | 627.03 |
|  |  |  | 0.09 | 0.51 | -0.28 | -0.03 | -0.23 | -0.44 | 0.01 | - | 1.18 | 628.88 |
|  | Twitter | Heat | 0.09 | 0.52 | -0.28 | -0.03 | -0.23 | -0.44 | 0.00 | 0.01 | 1.18 | 630.75 |
|  |  | AC | 0.10 | 0.52 | -0.27 | -0.02 | -0.22 | -0.43 | 0.01 | 0.01 | 1.18 | 630.85 |
|  | Google Search | AC repair | 0.09 | 0.53 | -0.31 | -0.03 | -0.25 | -0.45 | 0.02 | 0.00 | 1.17 | 630.68 |
|  |  | Beer | 0.10 | 0.53 | -0.18 | 0.27 | 0.04 | -0.45 | 0.02 | -0.01 | 1.17 | 630.06 |
|  |  | Drink | 0.12 | 0.55 | -0.23 | 0.16 | -0.10 | -0.44 | 0.02 | -0.01 | 1.17 | 630.33 |
|  |  | Heat exhaustion | 0.10 | 0.52 | -0.26 | -0.01 | -0.22 | -0.41 | 0.01 | 0.00 | 1.17 | 630.60 |
|  |  | Heat stroke | 0.14 | 0.59 | -0.29 | -0.02 | -0.17 | -0.43 | 0.03 | -0.01* | 1.16 | 628.16 |
|  |  | Hot weather | 0.08 | 0.52 | -0.28 | -0.05 | -0.23 | -0.44 | 0.01 | 0.00 | 1.18 | 630.67 |
|  |  | Park | 0.09 | 0.52 | -0.26 | 0.04 | -0.20 | -0.43 | 0.01 | 0.00 | 1.17 | 630.80 |
|  |  | Pool | 0.09 | 0.52 | -0.27 | 0.08 | -0.11 | -0.41 | 0.02 | -0.01 | 1.17 | 630.23 |
|  |  | Swim | 0.07 | 0.49 | -0.30 | 0.00 | -0.21 | -0.45 | 0.02 | -0.01 | 1.17 | 630.03 |
|  |  | Tired | 0.07 | 0.56 | -0.23 | 0.02 | -0.21 | -0.40 | 0.01 | 0.01 | 1.17 | 629.79 |
|  |  | water | 0.08 | 0.50 | -0.30 | -0.01 | -0.19 | -0.40 | 0.02 | -0.01 | 1.17 | 630.53 |

Table S12. Renal illness hospitalization model specifications (*** <0.05, ** 0.05 ~ 0.10, * 0.10 ~ 0.15)

| County | Keywords | | Wed | Thu | Fri | Sat | Sun | Mon | Max T | keyword | MAE | AIC |
| --- | --- | --- | --- | --- | --- | --- | --- | --- | --- | --- | --- | --- |
| Duval | None | | -0.71 | -4.09** | -3.34* | -11.79*** | -14.17*** | -1.21 | - | - | 5.70 | 1139.98 |
|  |  |  | -0.90 | -4.29*** | -3.54** | -11.91*** | -14.14*** | -1.12 | 0.19*** | - | 5.64 | 1137.77 |
|  | Twitter | Heat | -0.90 | -4.27*** | -3.53** | -11.92*** | -14.11*** | -1.15 | 0.18** | 0.05 | 5.63 | 1139.70 |
|  |  | AC | -0.74 | -4.34*** | -3.34* | -11.82*** | -14.02*** | -1.18 | 0.22*** | -0.45 | 5.61 | 1138.76 |
|  | Google Search | AC repair | -0.87 | -4.22*** | -3.26* | -11.59*** | -13.96*** | -0.84 | 0.18** | 0.03 | 5.63 | 1139.06 |
|  |  | Beer | -0.89 | -4.38*** | -3.34* | -11.38*** | -13.67*** | -1.13 | 0.21*** | -0.03 | 5.63 | 1139.10 |
|  |  | Drink | -0.88 | -4.29*** | -3.50** | -11.74*** | -13.93*** | -1.15 | 0.20*** | -0.01 | 5.64 | 1139.58 |
|  |  | Heat exhaustion | -0.99 | -4.29*** | -3.55** | -11.99*** | -14.16*** | -1.12 | 0.20*** | -0.01 | 5.63 | 1139.53 |
|  |  | Heat stroke | -1.03 | -4.30*** | -3.51** | -12.12*** | -14.00*** | -0.98 | 0.22*** | -0.04 | 5.58 | 1138.75 |
|  |  | Hot weather | -0.92 | -4.32*** | -3.52** | -11.85*** | -14.10*** | -1.10 | 0.20*** | -0.01 | 5.64 | 1139.65 |
|  |  | Park | -0.88 | -4.34*** | -3.10* | -11.17*** | -13.89*** | -0.87 | 0.25*** | -0.07 | 5.63 | 1138.40 |
|  |  | Pool | -1.08 | -4.20*** | -3.49** | -11.19*** | -13.55*** | -1.01 | 0.26*** | -0.06** | 5.61 | 1136.96 |
|  |  | Swim | -0.90 | -4.29*** | -3.54** | -11.92*** | -14.14*** | -1.12 | 0.19** | 0.00 | 5.64 | 1139.77 |
|  |  | Tired | -0.62 | -3.92** | -3.44* | -11.69*** | -13.92*** | -1.04 | 0.19*** | -0.02 | 5.63 | 1138.98 |
|  |  | water | -0.43 | -3.77** | -3.60** | -11.28*** | -13.50*** | -0.61 | 0.25*** | -0.14*** | 5.43 | 1132.71 |
| Hillsborough | None | | -2.35 | -1.09 | -1.68 | -11.01*** | -12.43*** | -0.97 | - | - | 5.33 | 1132.40 |
|  |  |  | -2.47 | -1.23 | -1.65 | -10.98*** | -12.36*** | -0.89 | 0.19 | - | 5.27 | 1132.37 |
|  | Twitter | Heat | -2.60 | -1.43 | -2.12 | -11.06*** | -12.33*** | -1.05 | 0.09 | 0.25** | 5.35 | 1131.04 |
|  |  | AC | -2.60 | -1.26 | -1.67 | -10.77*** | -12.20*** | -1.00 | 0.13 | 0.42* | 5.35 | 1132.17 |
|  | Google Search | AC repair | -2.52 | -1.32 | -1.72 | -11.16*** | -12.41*** | -0.96 | 0.17 | 0.01 | 5.30 | 1134.22 |
|  |  | Beer | -2.45 | -1.08 | -1.43 | -10.52*** | -12.05*** | -0.81 | 0.20 | -0.02 | 5.27 | 1134.27 |
|  |  | Drink | -2.71 | -1.23 | -1.61 | -10.44*** | -12.00*** | -1.23 | 0.21* | -0.05 | 5.27 | 1133.49 |
|  |  | Heat exhaustion | -2.59 | -1.49 | -1.68 | -11.04*** | -12.27*** | -0.75 | 0.24** | -0.03 | 5.29 | 1133.46 |
|  |  | Heat stroke | -2.47 | -1.38 | -1.66 | -11.12*** | -12.51*** | -1.08 | 0.20* | -0.02 | 5.25 | 1133.78 |
|  |  | Hot weather | -2.52 | -1.18 | -1.62 | -10.95*** | -12.36*** | -0.82 | 0.18 | 0.01 | 5.27 | 1134.15 |
|  |  | Park | -2.60 | -1.07 | -0.11 | -8.00*** | -10.58*** | -0.68 | 0.24** | -0.19*** | 5.22 | 1127.36 |
|  |  | Pool | -2.60 | -1.09 | -1.52 | -9.95*** | -11.43*** | -0.40 | 0.28** | -0.07* | 5.21 | 1131.70 |
|  |  | Swim | -2.33 | -1.44 | -1.58 | -10.58*** | -12.14*** | -0.59 | 0.24** | -0.06 | 5.21 | 1132.51 |
|  |  | Tired | -2.45 | -1.12 | -1.54 | -11.07*** | -12.58*** | -1.05 | 0.18 | 0.03 | 5.25 | 1133.17 |
|  |  | water | -2.58 | -1.15 | -1.60 | -11.16*** | -12.22*** | -0.64 | 0.27** | -0.13** | 5.18 | 1131.23 |
| Leon | None | | 0.06 | 0.04 | 0.20*** | -0.19** | -0.08 | -0.04 | - | - | 2.31 | 829.21 |
|  |  |  | 0.06 | 0.04 | 0.20*** | -0.18* | -0.08 | -0.04 | 0.00 | - | 2.27 | 830.45 |
|  | Twitter | Heat | 0.05 | 0.03 | 0.19** | -0.18** | -0.09 | -0.05 | -0.01 | 0.01 | 2.26 | 831.13 |
|  |  | AC | 0.06 | 0.04 | 0.20*** | -0.18** | -0.08 | -0.04 | -0.01 | 0.01 | 2.27 | 832.25 |
|  | Google Search | AC repair | 0.06 | 0.06 | 0.20*** | -0.17* | -0.08 | -0.03 | 0.00 | 0.00* | 2.26 | 829.85 |
|  |  | Beer | 0.06 | 0.04 | 0.21*** | -0.18* | -0.08 | -0.04 | 0.00 | 0.00 | 2.27 | 832.42 |
|  |  | Drink | 0.06 | 0.04 | 0.21*** | -0.17* | -0.07 | -0.04 | 0.00 | 0.00 | 2.27 | 832.08 |
|  |  | Heat exhaustion | 0.08 | 0.04 | 0.20*** | -0.18** | -0.07 | -0.01 | 0.00 | -0.01*** | 2.28 | 828.22 |
|  |  | Heat stroke | 0.06 | 0.03 | 0.20*** | -0.19** | -0.09 | -0.04 | 0.00 | 0.00 | 2.26 | 832.06 |
|  |  | Hot weather | 0.06 | 0.04 | 0.20** | -0.18** | -0.08 | -0.04 | 0.00 | 0.00 | 2.27 | 832.35 |
|  |  | Park | 0.06 | 0.04 | 0.20*** | -0.19** | -0.08 | -0.04 | 0.00 | 0.00 | 2.27 | 832.34 |
|  |  | Pool | 0.06 | 0.04 | 0.20** | -0.16* | -0.07 | -0.04 | 0.00 | 0.00 | 2.25 | 831.23 |
|  |  | Swim | 0.06 | 0.04 | 0.20*** | -0.19** | -0.09 | -0.04 | 0.00 | 0.00 | 2.27 | 832.37 |
|  |  | Tired | 0.06 | 0.04 | 0.21*** | -0.18* | -0.08 | -0.04 | 0.00 | 0.00 | 2.28 | 832.29 |
|  |  | water | 0.06 | 0.03 | 0.19** | -0.20** | -0.09 | -0.03 | 0.00 | 0.00 | 2.26 | 830.98 |
| Miami-Dade | None | | -2.22 | -0.52 | -4.01* | -19.68*** | -21.80*** | 1.62 | - | - | 7.31 | 1214.58 |
|  |  |  | -2.21 | -0.53 | -4.02* | -19.71*** | -21.80*** | 1.63 | 0.04 | - | 7.31 | 1216.55 |
|  | Twitter | Heat | -2.27 | -0.56 | -4.20* | -19.71*** | -21.81*** | 1.53 | 0.02 | 0.02 | 7.28 | 1218.21 |
|  |  | AC | -2.20 | -0.61 | -3.97* | -19.82*** | -21.88*** | 1.67 | 0.06 | -0.06 | 7.29 | 1218.39 |
|  | Google Search | AC repair | -2.16 | -0.40 | -3.88* | -19.39*** | -21.15*** | 1.71 | -0.05 | 0.06* | 7.31 | 1216.03 |
|  |  | Beer | -2.16 | -0.46 | -3.46 | -18.64*** | -20.89*** | 1.64 | 0.07 | -0.06 | 7.30 | 1217.69 |
|  |  | Drink | -1.52 | 0.08 | -3.06 | -17.84*** | -19.95*** | 1.93 | 0.03 | -0.10** | 7.29 | 1215.73 |
|  |  | Heat exhaustion | -2.18 | -0.51 | -4.03* | -19.70*** | -21.74*** | 1.66 | 0.05 | -0.01 | 7.30 | 1218.47 |
|  |  | Heat stroke | -2.49 | -0.73 | -4.32* | -20.01*** | -21.57*** | 2.03 | 0.10 | -0.05** | 7.27 | 1215.76 |
|  |  | Hot weather | -2.24 | -0.50 | -3.98* | -19.66*** | -21.72*** | 1.72 | 0.05 | -0.01 | 7.32 | 1218.35 |
|  |  | Park | -2.11 | -0.37 | -3.32 | -18.35*** | -20.69*** | 2.10 | 0.12 | -0.09 | 7.30 | 1217.34 |
|  |  | Pool | -2.25 | -0.54 | -4.01* | -19.49*** | -21.43*** | 1.75 | 0.09 | -0.03 | 7.32 | 1218.28 |
|  |  | Swim | -2.16 | -0.51 | -4.11* | -19.68*** | -21.83*** | 1.72 | 0.09 | -0.03 | 7.33 | 1217.93 |
|  |  | Tired | -2.21 | -0.58 | -4.08* | -19.68*** | -21.80*** | 1.58 | 0.04 | -0.01 | 7.31 | 1218.52 |
|  |  | water | -2.10 | -0.59 | -3.87* | -19.55*** | -21.36*** | 2.00 | 0.16 | -0.09 | 7.25 | 1217.40 |
| Orange | None | | -0.67 | 0.00 | -2.50 | -7.83*** | -9.33*** | 2.17 | - | - | 5.21 | 1103.28 |
|  |  |  | -0.77 | -0.11 | -2.61 | -7.89*** | -9.33*** | 2.14 | 0.10 | - | 5.19 | 1104.48 |
|  | Twitter | Heat | -0.76 | 0.11 | -2.50 | -7.85*** | -9.25*** | 2.31 | -0.15 | 0.25*** | 5.11 | 1095.91 |
|  |  | AC | -0.61 | 0.17 | -2.30 | -7.66*** | -9.11*** | 2.34 | 0.04 | 0.18 | 5.19 | 1105.73 |
|  | Google Search | AC repair | -0.78 | 0.01 | -2.79* | -7.92*** | -9.45*** | 2.12 | 0.13 | -0.02 | 5.18 | 1106.05 |
|  |  | Beer | -0.67 | 0.04 | -1.60 | -4.90*** | -6.64*** | 2.03 | 0.17 | -0.11*** | 5.09 | 1101.69 |
|  |  | Drink | -0.42 | 0.28 | -2.01 | -5.85*** | -7.91*** | 2.19 | 0.20* | -0.09** | 5.11 | 1102.93 |
|  |  | Heat exhaustion | -0.75 | -0.09 | -2.56 | -7.84*** | -9.31*** | 2.22 | 0.09 | 0.01 | 5.19 | 1106.39 |
|  |  | Heat stroke | -0.95 | -0.42 | -2.59 | -7.94*** | -9.56*** | 2.07 | 0.04 | 0.04* | 5.16 | 1104.03 |
|  |  | Hot weather | -0.83 | -0.01 | -2.56 | -8.09*** | -9.36*** | 2.19 | 0.09 | 0.02 | 5.18 | 1105.38 |
|  |  | Park | -0.81 | 0.21 | -1.62 | -5.35*** | -8.30*** | 2.49 | 0.20** | -0.17*** | 5.07 | 1099.81 |
|  |  | Pool | -0.79 | 0.03 | -2.45 | -6.78*** | -8.10*** | 2.48 | 0.23** | -0.08*** | 5.08 | 1102.40 |
|  |  | Swim | -0.90 | -0.27 | -2.74* | -7.70*** | -9.17*** | 2.10 | 0.19* | -0.04 | 5.13 | 1104.36 |
|  |  | Tired | -0.69 | -0.28 | -2.80* | -8.08*** | -9.42*** | 1.98 | 0.12 | -0.03 | 5.19 | 1105.64 |
|  |  | water | -0.82 | -0.16 | -2.65 | -7.83*** | -9.16*** | 2.31 | 0.14 | -0.03 | 5.18 | 1106.21 |

Table S13. Renal illness ED model specifications (*** <0.05, ** 0.05 ~ 0.10, * 0.10 ~ 0.15)

|  |  |  | Lag 0 | | | Lag 1 | | | Lag 2 | | | Lag 3 | | |
| --- | --- | --- | --- | --- | --- | --- | --- | --- | --- | --- | --- | --- | --- | --- |
|  | Keywords | | keyword | MAE | AIC | keyword | MAE | AIC | keyword | MAE | AIC | keyword | MAE | AIC |
| Duval | Twitter | Heat | 0.01 | 0.89 | 538.72 | 0.05* | 0.89 | 536.55 | 0.05* | 0.89 | 536.09 | 0.03 | 0.89 | 538.17 |
|  |  | AC | -0.06 | 0.89 | 538.16 | 0.19*** | 0.89 | 532.20 | -0.03 | 0.89 | 538.65 | 0.03 | 0.89 | 538.69 |
|  | Google Search | AC repair | 0.00 | 0.89 | 538.81 | 0.01 | 0.89 | 536.71 | 0.00 | 0.89 | 538.80 | 0.00 | 0.90 | 538.23 |
|  |  | Beer | -0.01 | 0.89 | 538.07 | 0.00 | 0.89 | 538.77 | 0.00 | 0.89 | 538.80 | 0.00 | 0.89 | 538.21 |
|  |  | Drink | 0.00 | 0.89 | 538.38 | 0.00 | 0.89 | 538.44 | 0.00 | 0.89 | 538.70 | 0.00 | 0.89 | 537.94 |
|  |  | Heat exhaustion | 0.00 | 0.89 | 538.64 | 0.00 | 0.90 | 538.25 | 0.00 | 0.88 | 537.66 | 0.00 | 0.90 | 537.70 |
|  |  | Heat stroke | 0.01*** | 0.89 | 534.18 | 0.01*** | 0.88 | 534.19 | 0.00 | 0.89 | 538.20 | 0.00 | 0.89 | 538.44 |
|  |  | Hot weather | 0.00 | 0.89 | 538.83 | 0.00 | 0.89 | 538.55 | 0.01 | 0.89 | 536.95 | 0.01* | 0.88 | 536.63 |
|  |  | Park | 0.02** | 0.89 | 535.86 | 0.01 | 0.89 | 538.03 | 0.03*** | 0.90 | 531.66 | 0.02** | 0.89 | 536.04 |
|  |  | Pool | 0.02*** | 0.87 | 530.94 | 0.00 | 0.89 | 538.41 | 0.01 | 0.89 | 537.20 | 0.02*** | 0.88 | 529.76 |
|  |  | Swim | 0.01** | 0.89 | 534.95 | 0.00 | 0.90 | 538.42 | 0.01* | 0.89 | 536.36 | 0.00 | 0.89 | 538.14 |
|  |  | Tired | 0.00 | 0.89 | 538.64 | 0.00 | 0.89 | 538.53 | 0.00 | 0.89 | 538.82 | 0.00 | 0.89 | 538.44 |
|  |  | water | 0.01* | 0.88 | 536.64 | 0.01* | 0.89 | 536.29 | 0.01 | 0.90 | 537.67 | 0.00 | 0.89 | 538.83 |
| Hillsborough | Twitter | Heat | -0.01 | 1.05 | 546.79 | 0.00 | 1.05 | 547.02 | -0.01 | 1.05 | 546.57 | -0.01 | 1.05 | 546.46 |
|  |  | AC | -0.05 | 1.04 | 544.99 | -0.06** | 1.02 | 543.85 | -0.06** | 1.04 | 544.22 | -0.01 | 1.05 | 546.87 |
|  | Google Search | AC repair | 0.00 | 1.05 | 546.44 | 0.00 | 1.04 | 546.14 | -0.01 | 1.04 | 545.59 | 0.00 | 1.05 | 546.60 |
|  |  | Beer | 0.00 | 1.05 | 547.00 | 0.01** | 1.04 | 544.24 | -0.01 | 1.05 | 546.06 | 0.01 | 1.03 | 545.79 |
|  |  | Drink | -0.01*** | 1.02 | 542.16 | 0.00 | 1.04 | 546.90 | 0.00 | 1.04 | 546.40 | -0.01* | 1.04 | 544.98 |
|  |  | Heat exhaustion | 0.00 | 1.04 | 546.16 | 0.00 | 1.05 | 546.93 | 0.00 | 1.05 | 546.75 | 0.00 | 1.05 | 545.22 |
|  |  | Heat stroke | 0.00 | 1.05 | 546.82 | -0.01*** | 1.04 | 543.00 | 0.00 | 1.05 | 546.89 | 0.00 | 1.05 | 546.90 |
|  |  | Hot weather | 0.00 | 1.05 | 546.89 | 0.00 | 1.05 | 546.80 | 0.00 | 1.05 | 546.67 | 0.00 | 1.05 | 546.98 |
|  |  | Park | 0.00 | 1.05 | 546.91 | -0.01 | 1.05 | 546.27 | -0.01 | 1.04 | 545.48 | -0.01 | 1.04 | 545.66 |
|  |  | Pool | 0.00 | 1.05 | 546.45 | 0.00 | 1.05 | 546.28 | -0.01*** | 1.03 | 540.75 | -0.01* | 1.04 | 544.61 |
|  |  | Swim | -0.01** | 1.04 | 543.44 | -0.01*** | 1.04 | 541.46 | 0.00 | 1.05 | 546.57 | 0.00 | 1.05 | 546.99 |
|  |  | Tired | 0.00 | 1.04 | 546.64 | 0.00 | 1.05 | 546.71 | 0.00 | 1.04 | 545.67 | 0.00 | 1.05 | 546.90 |
|  |  | water | 0.01 | 1.04 | 546.37 | -0.01 | 1.05 | 545.90 | -0.01 | 1.05 | 546.42 | 0.00 | 1.05 | 547.02 |
| Leon | Twitter | Heat | 0.02 | 0.66 | 360.45 | 0.00 | 0.66 | 360.72 | 0.01 | 0.66 | 360.71 | 0.02 | 0.66 | 360.32 |
|  |  | AC | -0.08 | 0.66 | 360.04 | 0.01 | 0.66 | 360.73 | 0.08 | 0.66 | 360.02 | 0.18*** | 0.64 | 356.65 |
|  | Google Search | AC repair | 0.01** | 0.64 | 357.54 | 0.00 | 0.66 | 359.72 | 0.00 | 0.66 | 360.73 | 0.00 | 0.66 | 360.43 |
|  |  | Beer | 0.00 | 0.66 | 360.20 | 0.00 | 0.66 | 360.48 | 0.00 | 0.66 | 360.71 | 0.00 | 0.66 | 360.72 |
|  |  | Drink | -0.03*** | 0.63 | 351.73 | -0.01 | 0.66 | 359.58 | 0.00 | 0.66 | 360.72 | 0.00 | 0.66 | 360.73 |
|  |  | Heat exhaustion | 0.00 | 0.66 | 360.67 | 0.00 | 0.66 | 360.73 | 0.00 | 0.66 | 360.39 | 0.01 | 0.66 | 359.40 |
|  |  | Heat stroke | -0.02** | 0.64 | 355.28 | 0.01*** | 0.65 | 357.29 | 0.01* | 0.66 | 358.65 | 0.01* | 0.65 | 358.62 |
|  |  | Hot weather | 0.00 | 0.66 | 360.07 | 0.00 | 0.66 | 360.72 | 0.01* | 0.66 | 358.85 | 0.00 | 0.66 | 360.71 |
|  |  | Park | 0.00 | 0.66 | 360.73 | -0.01* | 0.65 | 358.40 | -0.02** | 0.65 | 357.71 | -0.01* | 0.65 | 358.50 |
|  |  | Pool | 0.00 | 0.66 | 360.17 | -0.01 | 0.66 | 359.78 | 0.00 | 0.66 | 360.08 | -0.01 | 0.66 | 359.47 |
|  |  | Swim | 0.00 | 0.66 | 360.13 | -0.01 | 0.66 | 359.96 | 0.00 | 0.66 | 360.73 | -0.01 | 0.66 | 359.72 |
|  |  | Tired | 0.00 | 0.66 | 360.69 | 0.00 | 0.66 | 360.60 | 0.00 | 0.66 | 360.10 | -0.01*** | 0.62 | 350.79 |
|  |  | water | -0.01 | 0.66 | 360.09 | 0.00 | 0.66 | 360.69 | -0.01** | 0.65 | 357.35 | 0.00 | 0.66 | 360.67 |
| Miami-Dade | Twitter | Heat | 0.00 | 1.96 | 795.87 | 0.01 | 1.96 | 795.64 | 0.00 | 1.97 | 795.80 | 0.00 | 1.96 | 795.78 |
|  |  | AC | 0.07** | 1.94 | 792.75 | 0.00 | 1.96 | 795.86 | -0.03 | 1.97 | 795.52 | 0.05 | 1.95 | 794.61 |
|  | Google Search | AC repair | -0.01 | 1.96 | 795.29 | 0.01 | 1.96 | 795.02 | -0.01 | 1.97 | 795.53 | 0.00 | 1.96 | 795.75 |
|  |  | Beer | 0.02 | 1.95 | 794.44 | 0.00 | 1.97 | 795.85 | 0.02* | 1.96 | 793.62 | -0.01 | 1.96 | 795.73 |
|  |  | Drink | 0.00 | 1.96 | 795.84 | -0.02 | 1.97 | 794.17 | 0.03** | 1.96 | 792.56 | -0.04*** | 1.96 | 791.43 |
|  |  | Heat exhaustion | 0.01 | 1.95 | 794.43 | -0.01 | 1.96 | 795.38 | 0.02*** | 1.94 | 791.64 | -0.01 | 1.97 | 795.29 |
|  |  | Heat stroke | -0.01 | 1.95 | 795.13 | -0.02*** | 1.96 | 791.90 | -0.02** | 1.94 | 792.26 | -0.01** | 1.97 | 792.58 |
|  |  | Hot weather | 0.00 | 1.97 | 795.60 | -0.01** | 1.95 | 792.91 | -0.01** | 1.95 | 792.87 | 0.00 | 1.97 | 795.66 |
|  |  | Park | 0.00 | 1.96 | 795.87 | -0.01 | 1.96 | 795.68 | -0.04** | 1.96 | 793.02 | -0.05*** | 1.92 | 790.13 |
|  |  | Pool | -0.02 | 1.97 | 794.84 | -0.02 | 1.97 | 794.66 | -0.01 | 1.97 | 795.67 | -0.03** | 1.95 | 792.86 |
|  |  | Swim | -0.01 | 1.95 | 795.19 | -0.01 | 1.97 | 795.20 | -0.02* | 1.95 | 793.17 | -0.01 | 1.96 | 795.31 |
|  |  | Tired | 0.02 | 1.98 | 794.56 | 0.02* | 1.95 | 793.25 | 0.02 | 1.97 | 794.68 | 0.03*** | 1.95 | 791.86 |
|  |  | Water | -0.01 | 1.96 | 795.82 | -0.03 | 1.96 | 793.91 | 0.00 | 1.96 | 795.87 | 0.00 | 1.96 | 795.87 |
| Orange | Twitter | Heat | 0.01 | 1.18 | 630.75 | 0.00 | 1.18 | 630.88 | -0.01 | 1.16 | 630.24 | 0.01 | 1.18 | 630.80 |
|  |  | AC | 0.01 | 1.18 | 630.85 | 0.00 | 1.18 | 630.88 | -0.07* | 1.17 | 628.63 | 0.01 | 1.18 | 630.82 |
|  | Google Search | AC repair | 0.00 | 1.17 | 630.68 | 0.00 | 1.18 | 630.43 | 0.00 | 1.17 | 630.83 | 0.00 | 1.17 | 630.54 |
|  |  | Beer | -0.01 | 1.17 | 630.06 | 0.00 | 1.18 | 630.82 | 0.00 | 1.18 | 630.88 | 0.00 | 1.18 | 630.83 |
|  |  | Drink | -0.01 | 1.17 | 630.33 | -0.01 | 1.16 | 629.05 | -0.01 | 1.17 | 630.47 | 0.00 | 1.18 | 630.76 |
|  |  | Heat exhaustion | 0.00 | 1.17 | 630.60 | 0.00 | 1.18 | 630.85 | 0.00 | 1.18 | 630.81 | 0.00 | 1.17 | 630.36 |
|  |  | Heat stroke | -0.01* | 1.16 | 628.16 | 0.01 | 1.17 | 629.12 | 0.00 | 1.18 | 630.70 | 0.00 | 1.17 | 630.71 |
|  |  | Hot weather | 0.00 | 1.18 | 630.67 | 0.00 | 1.18 | 630.84 | 0.00 | 1.17 | 630.58 | 0.00 | 1.17 | 630.81 |
|  |  | Park | 0.00 | 1.17 | 630.80 | -0.02 | 1.17 | 629.83 | -0.01 | 1.17 | 630.45 | 0.00 | 1.18 | 630.83 |
|  |  | Pool | -0.01 | 1.17 | 630.23 | -0.01 | 1.17 | 629.42 | -0.01 | 1.16 | 629.78 | -0.01* | 1.16 | 628.50 |
|  |  | Swim | -0.01 | 1.17 | 630.03 | -0.01** | 1.16 | 627.15 | 0.00 | 1.18 | 630.87 | -0.01 | 1.17 | 630.12 |
|  |  | Tired | 0.01 | 1.17 | 629.79 | 0.01 | 1.18 | 629.51 | 0.00 | 1.18 | 630.53 | 0.00 | 1.18 | 630.87 |
|  |  | water | -0.01 | 1.17 | 630.53 | 0.00 | 1.18 | 630.87 | -0.02 | 1.16 | 629.57 | 0.01 | 1.18 | 630.59 |

Table S14. Renal illness hospitalization model specifications (*** <0.05, ** 0.05 ~ 0.10, * 0.10 ~ 0.15)

|  |  |  | Lag 0 | | | Lag 1 | | | Lag 2 | | | Lag 3 | | |
| --- | --- | --- | --- | --- | --- | --- | --- | --- | --- | --- | --- | --- | --- | --- |
|  | Keywords | | keyword | MAE | AIC | keyword | MAE | AIC | keyword | MAE | AIC | keyword | MAE | AIC |
| Duval | Twitter | Heat | 0.05 | 5.63 | 1139.70 | -0.04 | 5.63 | 1139.73 | -0.01 | 5.64 | 1139.77 | -0.16 | 5.62 | 1139.08 |
|  |  | AC | -0.45 | 5.61 | 1138.76 | -0.28 | 5.62 | 1139.41 | 0.16 | 5.63 | 1139.64 | -0.26 | 5.64 | 1139.43 |
|  | Google Search | AC repair | 0.03 | 5.63 | 1139.06 | 0.02 | 5.63 | 1139.12 | 0.02 | 5.65 | 1139.39 | 0.00 | 5.64 | 1139.77 |
|  |  | Beer | -0.03 | 5.63 | 1139.10 | -0.03 | 5.61 | 1139.15 | -0.01 | 5.64 | 1139.75 | -0.01 | 5.64 | 1139.75 |
|  |  | Drink | -0.01 | 5.64 | 1139.58 | -0.05 | 5.62 | 1137.72 | 0.02 | 5.63 | 1139.17 | -0.04 | 5.62 | 1138.47 |
|  |  | Heat exhaustion | -0.01 | 5.63 | 1139.53 | -0.01 | 5.63 | 1139.40 | 0.00 | 5.64 | 1139.76 | 0.00 | 5.64 | 1139.77 |
|  |  | Heat stroke | -0.04 | 5.58 | 1138.75 | -0.06** | 5.54 | 1136.67 | 0.05* | 5.57 | 1137.42 | -0.01 | 5.63 | 1139.63 |
|  |  | Hot weather | -0.01 | 5.64 | 1139.65 | 0.03 | 5.59 | 1138.27 | 0.01 | 5.61 | 1139.58 | 0.02 | 5.61 | 1139.35 |
|  |  | Park | -0.07 | 5.63 | 1138.40 | 0.01 | 5.64 | 1139.75 | -0.05 | 5.61 | 1139.14 | -0.09* | 5.59 | 1137.41 |
|  |  | Pool | -0.06** | 5.61 | 1136.96 | -0.03 | 5.62 | 1138.72 | -0.04 | 5.57 | 1137.97 | -0.10*** | 5.51 | 1130.86 |
|  |  | Swim | 0.00 | 5.64 | 1139.77 | -0.02 | 5.60 | 1139.14 | -0.06** | 5.55 | 1135.97 | -0.03 | 5.59 | 1138.45 |
|  |  | Tired | -0.02 | 5.63 | 1138.98 | 0.00 | 5.64 | 1139.76 | 0.04* | 5.65 | 1137.54 | 0.01 | 5.63 | 1139.67 |
|  |  | water | -0.14*** | 5.43 | 1132.71 | -0.08 | 5.66 | 1137.73 | -0.03 | 5.64 | 1139.42 | -0.02 | 5.63 | 1139.55 |
| Hillsborough | Twitter | Heat | 0.25** | 5.35 | 1131.04 | 0.32*** | 5.27 | 1129.07 | 0.13 | 5.31 | 1133.51 | -0.19 | 5.20 | 1132.62 |
|  |  | AC | 0.42* | 5.35 | 1132.17 | 0.28 | 5.27 | 1133.42 | 0.40 | 5.31 | 1132.56 | 0.11 | 5.27 | 1134.23 |
|  | Google Search | AC repair | 0.01 | 5.30 | 1134.22 | -0.02 | 5.28 | 1134.16 | -0.02 | 5.25 | 1133.87 | 0.00 | 5.27 | 1134.36 |
|  |  | Beer | -0.02 | 5.27 | 1134.27 | 0.04 | 5.28 | 1133.82 | -0.04 | 5.27 | 1133.65 | -0.13*** | 5.18 | 1127.93 |
|  |  | Drink | -0.05 | 5.27 | 1133.49 | 0.07 | 5.37 | 1132.52 | -0.01 | 5.27 | 1134.32 | -0.04 | 5.25 | 1133.76 |
|  |  | Heat exhaustion | -0.03 | 5.29 | 1133.46 | 0.05** | 5.29 | 1130.91 | -0.01 | 5.27 | 1134.31 | -0.01 | 5.27 | 1134.26 |
|  |  | Heat stroke | -0.02 | 5.25 | 1133.78 | 0.00 | 5.27 | 1134.34 | 0.01 | 5.24 | 1134.07 | -0.03 | 5.18 | 1132.54 |
|  |  | Hot weather | 0.01 | 5.27 | 1134.15 | -0.01 | 5.28 | 1134.19 | -0.02 | 5.26 | 1133.79 | 0.02 | 5.31 | 1134.05 |
|  |  | Park | -0.19*** | 5.22 | 1127.36 | -0.15*** | 5.21 | 1129.89 | -0.08 | 5.24 | 1133.11 | -0.08 | 5.26 | 1133.12 |
|  |  | Pool | -0.07* | 5.21 | 1131.70 | -0.05 | 5.21 | 1133.06 | -0.04 | 5.23 | 1133.45 | -0.05 | 5.21 | 1132.66 |
|  |  | Swim | -0.06 | 5.21 | 1132.51 | -0.05 | 5.25 | 1133.16 | -0.03 | 5.25 | 1133.92 | -0.07* | 5.20 | 1131.93 |
|  |  | Tired | 0.03 | 5.25 | 1133.17 | 0.02 | 5.28 | 1133.95 | 0.00 | 5.28 | 1134.37 | -0.03 | 5.26 | 1133.43 |
|  |  | water | -0.13** | 5.18 | 1131.23 | -0.10 | 5.20 | 1132.53 | -0.15*** | 5.23 | 1130.02 | 0.01 | 5.28 | 1134.36 |
| Leon | Twitter | Heat | 0.01 | 2.26 | 831.13 | 0.01 | 2.27 | 830.69 | 0.02* | 2.24 | 829.97 | -0.01 | 2.28 | 831.78 |
|  |  | AC | 0.01 | 2.27 | 832.25 | 0.07*** | 2.22 | 826.57 | 0.02 | 2.25 | 831.82 | 0.01 | 2.27 | 832.33 |
|  | Google Search | AC repair | 0.00* | 2.26 | 829.85 | 0.00 | 2.27 | 832.43 | 0.00 | 2.27 | 832.12 | 0.00 | 2.27 | 832.44 |
|  |  | Beer | 0.00 | 2.27 | 832.42 | 0.00 | 2.27 | 832.45 | -0.03*** | 2.25 | 837.34 | 0.00 | 2.27 | 831.98 |
|  |  | Drink | 0.00 | 2.27 | 832.08 | 0.00 | 2.27 | 832.29 | 0.00 | 2.27 | 832.33 | 0.00 | 2.27 | 830.50 |
|  |  | Heat exhaustion | -0.01*** | 2.28 | 828.22 | 0.00 | 2.27 | 832.34 | 0.00 | 2.27 | 832.41 | 0.00 | 2.26 | 831.32 |
|  |  | Heat stroke | 0.00 | 2.26 | 832.06 | 0.00 | 2.26 | 830.99 | 0.00 | 2.27 | 832.33 | 0.00 | 2.27 | 832.42 |
|  |  | Hot weather | 0.00 | 2.27 | 832.35 | 0.03*** | 2.23 | 836.56 | 0.00 | 2.27 | 832.19 | 0.00 | 2.26 | 831.65 |
|  |  | Park | 0.00 | 2.27 | 832.34 | 0.00 | 2.26 | 831.51 | 0.00 | 2.27 | 832.45 | 0.00 | 2.27 | 831.72 |
|  |  | Pool | 0.00 | 2.25 | 831.23 | 0.00 | 2.27 | 831.24 | 0.00 | 2.27 | 832.29 | 0.00 | 2.26 | 831.68 |
|  |  | Swim | 0.00 | 2.27 | 832.37 | 0.00 | 2.27 | 832.43 | 0.00 | 2.27 | 832.45 | 0.00* | 2.22 | 830.24 |
|  |  | Tired | 0.00 | 2.28 | 832.29 | 0.00 | 2.27 | 831.89 | 0.00 | 2.27 | 832.35 | 0.00 | 2.25 | 831.16 |
|  |  | water | 0.00 | 2.26 | 830.98 | 0.00 | 2.27 | 832.44 | 0.00 | 2.27 | 831.93 | 0.00** | 2.25 | 829.61 |
| Miami-Dade | Twitter | Heat | 0.02 | 7.28 | 1218.21 | 0.00 | 7.31 | 1218.54 | -0.04 | 7.32 | 1217.67 | 0.02 | 7.27 | 1218.20 |
|  |  | AC | -0.06 | 7.29 | 1218.39 | 0.03 | 7.32 | 1218.51 | -0.09 | 7.32 | 1218.17 | -0.04 | 7.30 | 1218.47 |
|  | Google Search | AC repair | 0.06* | 7.31 | 1216.03 | -0.03 | 7.28 | 1217.85 | -0.06** | 7.22 | 1215.68 | -0.06** | 7.24 | 1215.71 |
|  |  | Beer | -0.06 | 7.30 | 1217.69 | 0.01 | 7.31 | 1218.54 | 0.06 | 7.29 | 1217.47 | -0.05 | 7.31 | 1217.81 |
|  |  | Drink | -0.10** | 7.29 | 1215.73 | 0.07 | 7.29 | 1217.24 | -0.08 | 7.24 | 1217.01 | -0.09* | 7.21 | 1216.29 |
|  |  | Heat exhaustion | -0.01 | 7.30 | 1218.47 | -0.02 | 7.29 | 1218.11 | -0.01 | 7.31 | 1218.47 | -0.02 | 7.32 | 1218.25 |
|  |  | Heat stroke | -0.05** | 7.27 | 1215.76 | -0.05* | 7.18 | 1215.97 | 0.02 | 7.31 | 1218.10 | 0.04 | 7.27 | 1216.64 |
|  |  | Hot weather | -0.01 | 7.32 | 1218.35 | -0.03 | 7.30 | 1217.60 | -0.04* | 7.27 | 1216.09 | -0.02 | 7.31 | 1218.02 |
|  |  | Park | -0.09 | 7.30 | 1217.34 | -0.05 | 7.31 | 1218.21 | -0.04 | 7.29 | 1218.33 | -0.06 | 7.28 | 1218.05 |
|  |  | Pool | -0.03 | 7.32 | 1218.28 | -0.06 | 7.27 | 1217.35 | -0.13*** | 7.12 | 1213.15 | -0.09* | 7.19 | 1215.95 |
|  |  | Swim | -0.03 | 7.33 | 1217.93 | -0.08*** | 7.20 | 1214.06 | -0.01 | 7.31 | 1218.44 | -0.03 | 7.28 | 1217.94 |
|  |  | Tired | -0.01 | 7.31 | 1218.52 | -0.01 | 7.32 | 1218.53 | 0.01 | 7.31 | 1218.48 | -0.04 | 7.32 | 1217.85 |
|  |  | Water | -0.09 | 7.25 | 1217.40 | -0.15** | 7.25 | 1215.58 | -0.01 | 7.31 | 1218.55 | -0.14* | 7.24 | 1215.95 |
| Orange | Twitter | Heat | 0.25*** | 5.11 | 1095.91 | 0.03 | 5.18 | 1106.31 | 0.08 | 5.18 | 1105.21 | 0.03 | 5.19 | 1106.32 |
|  |  | AC | 0.18 | 5.19 | 1105.73 | 0.05 | 5.19 | 1106.42 | -0.35** | 5.19 | 1103.35 | -0.20 | 5.16 | 1105.42 |
|  | Google Search | AC repair | -0.02 | 5.18 | 1106.05 | 0.03 | 5.18 | 1105.36 | -0.03 | 5.17 | 1105.27 | -0.02 | 5.18 | 1105.68 |
|  |  | Beer | -0.11*** | 5.09 | 1101.69 | -0.08* | 5.17 | 1104.15 | -0.04 | 5.21 | 1105.98 | 0.00 | 5.19 | 1106.47 |
|  |  | Drink | -0.09** | 5.11 | 1102.93 | -0.08** | 5.16 | 1103.57 | -0.07* | 5.16 | 1103.95 | -0.07* | 5.18 | 1104.01 |
|  |  | Heat exhaustion | 0.01 | 5.19 | 1106.39 | 0.01 | 5.19 | 1106.22 | 0.01 | 5.19 | 1106.35 | -0.01 | 5.20 | 1106.33 |
|  |  | Heat stroke | 0.04* | 5.16 | 1104.03 | -0.02 | 5.23 | 1105.71 | -0.01 | 5.20 | 1106.14 | 0.00 | 5.19 | 1106.48 |
|  |  | Hot weather | 0.02 | 5.18 | 1105.38 | 0.02 | 5.24 | 1105.38 | 0.01 | 5.19 | 1106.19 | 0.02 | 5.18 | 1105.96 |
|  |  | Park | -0.17*** | 5.07 | 1099.81 | -0.12** | 5.11 | 1102.79 | -0.07 | 5.14 | 1105.23 | -0.09 | 5.14 | 1104.59 |
|  |  | Pool | -0.08*** | 5.08 | 1102.40 | -0.07** | 5.10 | 1103.07 | -0.09*** | 5.07 | 1101.21 | -0.06* | 5.14 | 1104.27 |
|  |  | Swim | -0.04 | 5.13 | 1104.36 | -0.06*** | 5.11 | 1102.31 | -0.05* | 5.09 | 1103.91 | -0.05** | 5.11 | 1103.28 |
|  |  | Tired | -0.03 | 5.19 | 1105.64 | 0.00 | 5.19 | 1106.48 | 0.01 | 5.18 | 1106.33 | -0.03 | 5.17 | 1105.72 |
|  |  | water | -0.03 | 5.18 | 1106.21 | -0.20*** | 5.03 | 1096.96 | -0.15*** | 5.12 | 1101.06 | -0.04 | 5.18 | 1106.04 |

Table S15. Cardiovascular illness ED model specifications (*** <0.05, ** 0.05 ~ 0.10, * 0.10 ~ 0.15)

| County | Keywords | | Model | Wed | Thu | Fri | Sat | Sun | Mon | Max T | keyword | MAE | AIC |
| --- | --- | --- | --- | --- | --- | --- | --- | --- | --- | --- | --- | --- | --- |
| Duval | None | | ARMA(2,1)-GARCH(1,1) | - | - | - | - | - | - | - | - | 16.11 | 8.99 |
|  |  |  |  | -5.72 | -6.88 | -14.65*** | -22.17*** | -27.78*** | 8.14** | - | - | 13.46 | 8.62 |
|  |  |  |  | -5.88 | -7.05* | -14.87*** | -22.31*** | -27.79*** | 8.18** | 0.20 | - | 13.38 | 8.63 |
|  | Twitter | Heat |  | -5.90 | -7.12* | -14.93*** | -22.33*** | -27.88*** | 8.20** | 0.21 | -0.12 | 13.39 | 8.64 |
|  |  | AC |  | -6.00 | -7.02* | -15.01*** | -22.37*** | -27.88*** | 8.22** | 0.19 | 0.31 | 13.41 | 8.64 |
|  | Google Search | AC repair |  | -5.75 | -6.74 | -13.77*** | -21.07*** | -27.09*** | 9.25*** | 0.18 | 0.09 | 13.31 | 8.63 |
|  |  | Beer |  | -5.93 | -7.55* | -14.06*** | -20.13*** | -25.90*** | 8.04** | 0.23 | -0.13 | 13.28 | 8.63 |
|  |  | Drink |  | -5.75 | -7.04* | -14.64*** | -21.41*** | -26.71*** | 8.01** | 0.22 | -0.07 | 13.40 | 8.64 |
|  |  | Heat exhaustion |  | -6.59 | -7.14 | -14.97** | -22.94*** | -27.98*** | 8.11 | 0.29 | -0.09* | 13.37 | 8.63 |
|  |  | Heat stroke |  | -5.60 | -7.01* | -14.94*** | -21.77*** | -28.18*** | 7.90** | 0.19 | 0.09 | 13.37 | 8.64 |
|  |  | Hot weather |  | -5.92 | -7.08* | -14.86*** | -22.28*** | -27.77*** | 8.17** | 0.20 | -0.01 | 13.38 | 8.64 |
|  |  | Park |  | -5.92 | -6.95* | -15.38*** | -23.15*** | -28.08*** | 7.92** | 0.17 | 0.08 | 13.38 | 8.64 |
|  |  | Pool |  | -6.47 | -6.87 | -13.61*** | -17.66*** | -24.19*** | 8.62** | -0.04 | -0.36*** | 13.46 | 8.64 |
|  |  | Swim |  | -5.62 | -7.03* | -14.30*** | -22.53*** | -28.08*** | 8.30** | 0.21 | 0.07 | 13.32 | 8.64 |
|  |  | Tired |  | -6.91* | -8.39** | -15.27*** | -23.15*** | -28.61*** | 7.89** | 0.23 | 0.09 | 13.30 | 8.63 |
|  |  | water |  | -6.06 | -7.22* | -14.87*** | -22.51*** | -28.00*** | 8.02** | 0.20 | 0.04 | 13.39 | 8.64 |
| Hillsborough | None | | ARMA(1,1)-GARCH(1,1) | - | - | - | - | - | - | - | - | 16.99 | 8.98 |
|  |  |  |  | -1.86 | -7.15 | -6.76 | -18.60*** | -20.08*** | 14.61*** | - | - | 14.59 | 8.72 |
|  |  |  |  | -2.54 | -7.96* | -6.61 | -18.55*** | -19.84*** | 15.11*** | 0.94*** | - | 14.13 | 8.67 |
|  | Twitter | Heat |  | -2.27 | -7.52* | -5.71 | -18.30*** | -19.76*** | 15.50*** | 1.11*** | -0.44* | 14.14 | 8.67 |
|  |  | AC |  | -2.54 | -8.00* | -6.63 | -18.73*** | -19.93*** | 15.14*** | 0.98*** | -0.21 | 14.09 | 8.68 |
|  | Google Search | AC repair |  | -2.29 | -7.52* | -6.26 | -17.71*** | -19.55*** | 15.46*** | 1.01*** | -0.06 | 14.17 | 8.68 |
|  |  | Beer |  | -2.47 | -7.53* | -6.02 | -17.33*** | -18.97*** | 15.35*** | 0.96*** | -0.04 | 14.13 | 8.68 |
|  |  | Drink |  | -4.05 | -7.84* | -6.22 | -14.73*** | -17.25*** | 12.73*** | 1.07*** | -0.32*** | 13.68 | 8.64 |
|  |  | Heat exhaustion |  | -2.86 | -8.80** | -6.62 | -18.71*** | -19.49*** | 15.60*** | 1.09*** | -0.09 | 13.94 | 8.67 |
|  |  | Heat stroke |  | -2.56 | -7.38* | -6.59 | -18.05*** | -19.25*** | 15.85*** | 0.90*** | 0.08 | 14.05 | 8.67 |
|  |  | Hot weather |  | -2.30 | -8.23** | -6.76 | -18.79*** | -19.83*** | 14.75*** | 1.00*** | -0.08 | 14.15 | 8.68 |
|  |  | Park |  | -2.68 | -7.66* | -4.05 | -13.69*** | -16.94*** | 15.50*** | 1.04*** | -0.31** | 13.99 | 8.66 |
|  |  | Pool |  | -2.50 | -7.90* | -6.54 | -18.37*** | -19.67*** | 15.23*** | 0.96*** | -0.01 | 14.12 | 8.68 |
|  |  | Swim |  | -2.53 | -7.97* | -6.58 | -18.50*** | -19.78*** | 15.14*** | 0.95*** | -0.01 | 14.12 | 8.68 |
|  |  | Tired |  | -2.49 | -7.80* | -6.45 | -18.68*** | -20.06*** | 14.94*** | 0.93*** | 0.04 | 14.05 | 8.68 |
|  |  | water |  | -2.42 | -8.07* | -6.67 | -18.38*** | -19.99*** | 14.78*** | 0.85*** | 0.14 | 14.04 | 8.68 |
| Leon | None | | ARMA(1,2)-GARCH(1,1) | - | - | - | - | - | - | - | - | 8.68 | 7.80 |
|  |  |  |  | 0.32 | -1.22 | 2.70 | -7.57*** | -5.62** | 2.38 | - | - | 8.05 | 7.75 |
|  |  |  |  | 0.17 | -1.24 | 2.59 | -7.79*** | -5.70** | 2.32 | 0.13 | - | 8.01 | 7.75 |
|  | Twitter | Heat |  | 0.17 | -1.25 | 2.60 | -7.80*** | -5.70** | 2.32 | 0.13 | 0.00 | 8.01 | 7.77 |
|  |  | AC |  | 0.16 | -1.21 | 2.56 | -7.83*** | -5.58** | 2.32 | 0.10 | 0.32 | 8.02 | 7.77 |
|  | Google Search | AC repair |  | 0.28 | -1.58 | 2.57 | -7.95*** | -5.68** | 2.28 | 0.12 | 0.03 | 7.97 | 7.76 |
|  |  | Beer |  | 0.24 | -1.03 | 2.86 | -7.34*** | -5.56** | 2.33 | 0.13 | -0.03 | 7.98 | 7.76 |
|  |  | Drink |  | 1.85 | -1.55 | 2.28 | -6.69*** | -4.92 | 2.43 | 0.00 | -0.06 | 8.54 | 7.82 |
|  |  | Heat exhaustion |  | 0.46 | -1.25 | 2.55 | -7.81*** | -5.55** | 2.69 | 0.14 | -0.05 | 8.04 | 7.76 |
|  |  | Heat stroke |  | 1.68 | -1.61 | 1.92 | -7.52*** | -5.58** | 2.50 | -0.04 | 0.02 | 8.51 | 7.83 |
|  |  | Hot weather |  | 0.25 | -1.18 | 2.74 | -7.80*** | -5.70** | 2.44 | 0.14 | -0.02 | 8.00 | 7.77 |
|  |  | Park |  | 0.17 | -1.24 | 2.60 | -7.79*** | -5.71** | 2.33 | 0.13 | 0.00 | 8.01 | 7.77 |
|  |  | Pool |  | - | - | - | - | - | - | - | - | - | - |
|  |  | Swim |  | 0.12 | -0.90 | 2.84 | -7.58*** | -5.48** | 2.58 | 0.16 | -0.03 | 8.00 | 7.76 |
|  |  | Tired |  | - | - | - | - | - | - | - | - | - | - |
|  |  | water |  | - | - | - | - | - | - | - | - | - | - |
| Miami-Dade | None | | ARMA(1,1)-GARCH(1,1) | - | - | - | - | - | - | - | - | 38.56 | 10.67 |
|  |  |  |  | -18.22 | -19.86 | -21.17 | -71.63 | -99.99** | 26.09 | - | - | 23.58 | 9.76 |
|  |  |  |  | -18.75*** | -20.51*** | -22.23*** | -73.17*** | -99.95*** | 26.08*** | 1.44*** | - | 23.40 | 9.76 |
|  | Twitter | Heat |  | -18.11*** | -20.70*** | -22.35*** | -72.86*** | -99.74*** | 26.14*** | 1.36** | 0.08 | 23.40 | 9.77 |
|  |  | AC |  | -18.25*** | -19.24*** | -22.29*** | -71.40*** | -98.74*** | 25.68*** | 1.04* | 0.72* | 23.40 | 9.76 |
|  | Google Search | AC repair |  | -18.03*** | -19.75*** | -21.18*** | -71.54*** | -96.68*** | 26.70*** | 0.92 | 0.28*** | 23.04 | 9.74 |
|  |  | Beer |  | -18.14*** | -20.18*** | -19.87*** | -69.32*** | -96.65*** | 26.28*** | 1.43*** | -0.19 | 23.45 | 9.77 |
|  |  | Drink |  | -16.02** | -18.57*** | -18.80*** | -67.38*** | -94.36*** | 27.02*** | 1.32*** | -0.29* | 23.38 | 9.77 |
|  |  | Heat exhaustion |  | -17.88*** | -20.11*** | -21.82*** | -72.96*** | -98.99*** | 26.60*** | 1.48*** | -0.10 | 23.27 | 9.76 |
|  |  | Heat stroke |  | -18.74*** | -20.80*** | -22.34*** | -73.63*** | -99.22*** | 27.22*** | 1.51*** | -0.13 | 23.58 | 9.77 |
|  |  | Hot weather |  | -18.53*** | -20.02*** | -21.31*** | -72.44*** | -98.82*** | 27.32*** | 1.41*** | -0.12 | 23.24 | 9.76 |
|  |  | Park |  | -17.42*** | -18.97*** | -15.08** | -59.81*** | -88.83*** | 30.96*** | 2.04*** | -0.86*** | 23.31 | 9.73 |
|  |  | Pool |  | -18.71*** | -20.80*** | -21.70*** | -70.69*** | -96.06*** | 27.79*** | 2.05*** | -0.34** | 23.19 | 9.76 |
|  |  | Swim |  | -18.47*** | -20.50*** | -21.85*** | -73.24*** | -99.77*** | 25.90*** | 1.37** | 0.06 | 23.45 | 9.77 |
|  |  | Tired |  | -18.28*** | -20.30*** | -21.63*** | -73.13*** | -99.19*** | 26.41*** | 1.27** | 0.02 | 23.41 | 9.77 |
|  |  | water |  | -18.02*** | -20.55*** | -21.37*** | -72.63*** | -98.53*** | 27.49*** | 1.75*** | -0.27 | 23.37 | 9.77 |
| Orange | None | | ARMA(1,1)-GARCH(1,1) | - | - | - | - | - | - | - | - | 17.03 | 9.06 |
|  |  |  |  | -3.16 | -7.62** | -18.31*** | -24.72*** | -30.09*** | 15.11*** | - | - | 12.66 | 8.52 |
|  |  |  |  | -3.22 | -7.69** | -18.37*** | -24.77*** | -30.14*** | 15.07*** | 0.02 | - | 12.66 | 8.53 |
|  | Twitter | Heat |  | -3.17 | -7.55** | -18.26*** | -24.69*** | -30.01*** | 15.22*** | -0.08 | 0.10 | 12.64 | 8.54 |
|  |  | AC |  | -3.20 | -7.67** | -18.36*** | -24.76*** | -30.12*** | 15.09*** | 0.02 | -0.01 | 12.66 | 8.54 |
|  | Google Search | AC repair |  | -2.69 | -8.65** | -16.06*** | -24.00*** | -28.54*** | 15.73*** | -0.29 | 0.21*** | 12.75 | 8.52 |
|  |  | Beer |  | -3.14 | -7.45** | -15.94*** | -17.09*** | -23.34*** | 14.49*** | 0.20 | -0.29*** | 12.58 | 8.51 |
|  |  | Drink |  | -3.27 | -7.74** | -18.46*** | -25.22*** | -30.42*** | 15.10*** | 0.00 | 0.02 | 12.65 | 8.54 |
|  |  | Heat exhaustion |  | -3.20 | -7.66** | -18.27*** | -24.68*** | -30.12*** | 15.21*** | 0.01 | 0.01 | 12.65 | 8.54 |
|  |  | Heat stroke |  | -3.24 | -7.72** | -18.35*** | -24.76*** | -30.15*** | 15.07*** | 0.02 | 0.01 | 12.65 | 8.54 |
|  |  | Hot weather |  | -3.23 | -7.39* | -18.14*** | -25.26*** | -30.17*** | 15.32*** | -0.01 | 0.06 | 12.57 | 8.53 |
|  |  | Park |  | -3.33 | -7.19* | -16.81*** | -20.65*** | -28.51*** | 15.53*** | 0.20 | -0.27** | 12.64 | 8.52 |
|  |  | Pool |  | -3.26 | -7.79** | -18.43*** | -24.80*** | -30.13*** | 15.01*** | 0.03 | 0.00 | 12.66 | 8.54 |
|  |  | Swim |  | -3.17 | -7.66** | -18.34*** | -24.68*** | -30.03*** | 15.13*** | 0.05 | -0.01 | 12.66 | 8.54 |
|  |  | Tired |  | -3.19 | -7.76** | -18.45*** | -24.85*** | -30.18*** | 15.00*** | 0.03 | -0.01 | 12.66 | 8.54 |
|  |  | water |  | -3.25 | -7.72** | -18.41*** | -24.72*** | -29.97*** | 15.23*** | 0.07 | -0.03 | 12.67 | 8.54 |

Table S16. Cardiovascular illness hospitalization model specifications (*** <0.05, ** 0.05 ~ 0.10, * 0.10 ~ 0.15)

| County | Keywords | | Model | Wed | Thu | Fri | Sat | Sun | Mon | Max T | keyword | MAE | AIC |
| --- | --- | --- | --- | --- | --- | --- | --- | --- | --- | --- | --- | --- | --- |
| Duval | None | | ARMA(1,1)-GARCH(1,1) | - | - | - | - | - | - | - | - | 29.05 | 10.02 |
|  |  |  |  | -12.20*** | -27.74*** | -34.00*** | -79.98*** | -89.72*** | -6.09 | - | - | 13.24 | 8.76 |
|  |  |  |  | -11.97*** | -27.54*** | -33.80*** | -79.84*** | -89.87*** | -6.33 | -0.27*** | - | 13.35 | 8.77 |
|  | Twitter | Heat |  | -11.92*** | -27.48*** | -33.73*** | -79.80*** | -89.78*** | -6.30 | -0.28*** | 0.05 | 13.37 | 8.78 |
|  |  | AC |  | -12.54*** | -27.47*** | -34.49*** | -80.16*** | -90.22*** | -6.20 | -0.36*** | 1.34 | 13.29 | 8.77 |
|  | Google Search | AC repair |  | -11.91*** | -27.46*** | -33.62*** | -79.65*** | -89.73*** | -6.14 | -0.27*** | 0.01 | 13.37 | 8.78 |
|  |  | Beer |  | -12.00*** | -28.31*** | -32.10*** | -75.34*** | -85.87*** | -6.48 | -0.11 | -0.26*** | 13.28 | 8.73 |
|  |  | Drink |  | -11.73*** | -27.45*** | -33.34*** | -78.16*** | -87.94*** | -6.58 | -0.18* | -0.12* | 13.34 | 8.76 |
|  |  | Heat exhaustion |  | -11.92*** | -27.53*** | -33.79*** | -79.81*** | -89.85*** | -6.32 | -0.27*** | 0.01 | 13.35 | 8.78 |
|  |  | Heat stroke |  | -12.17*** | -27.57*** | -33.77*** | -80.11*** | -89.67*** | -6.14 | -0.22* | -0.06 | 13.30 | 8.78 |
|  |  | Hot weather |  | -12.10*** | -27.79*** | -33.68*** | -79.36*** | -89.58*** | -6.25 | -0.26** | -0.06 | 13.35 | 8.77 |
|  |  | Park |  | -11.71*** | -27.74*** | -31.47*** | -75.87*** | -88.54*** | -5.02 | 0.03 | -0.36*** | 13.18 | 8.73 |
|  |  | Pool |  | -12.25*** | -27.23*** | -33.62*** | -78.10*** | -88.51*** | -6.00 | -0.12 | -0.12* | 13.22 | 8.76 |
|  |  | Swim |  | -12.13*** | -27.56*** | -34.09*** | -79.78*** | -89.77*** | -6.39 | -0.24** | -0.03 | 13.33 | 8.78 |
|  |  | Tired |  | -11.04*** | -26.35*** | -33.46*** | -79.12*** | -89.14*** | -6.05 | -0.27*** | -0.08 | 13.19 | 8.77 |
|  |  | water |  | -10.83*** | -26.30*** | -33.92*** | -78.29*** | -88.36*** | -5.07 | -0.12 | -0.35*** | 13.05 | 8.74 |
| Hillsborough | None | | ARMA(1,2)-GARCH(1,1) | - | - | - | - | - | - | - | - | 28.42 | 10.01 |
|  |  |  |  | -7.52* | -20.95*** | -29.54*** | -85.52*** | -99.36*** | -8.31* | - | - | 13.43 | 8.83 |
|  |  |  |  | -7.54* | -20.98*** | -29.48*** | -85.45*** | -99.27*** | -8.21* | 0.09 | - | 13.42 | 8.84 |
|  | Twitter | Heat |  | -7.65* | -21.19*** | -29.96*** | -85.56*** | -99.25*** | -8.38* | -0.01 | 0.25 | 13.35 | 8.85 |
|  |  | AC |  | -7.09*** | -21.55*** | -30.71*** | -87.80*** | -97.45*** | -6.15*** | -0.01*** | -0.56*** | 12.32 | 8.77 |
|  | Google Search | AC repair |  | -7.35 | -20.59*** | -29.15*** | -84.60*** | -99.08*** | -7.96* | 0.19 | -0.07 | 13.42 | 8.85 |
|  |  | Beer |  | -7.66* | -19.30*** | -26.67*** | -79.42*** | -94.98*** | -7.25 | 0.07 | -0.23** | 12.97 | 8.81 |
|  |  | Drink |  | -9.53*** | -21.35*** | -29.22*** | -82.39*** | -96.05*** | -9.97*** | 0.15*** | -0.26*** | 12.02 | 8.69 |
|  |  | Heat exhaustion |  | -7.63* | -21.13*** | -29.51*** | -85.51*** | -99.26*** | -8.18* | 0.13 | -0.02 | 13.41 | 8.85 |
|  |  | Heat stroke |  | -7.08*** | -22.39*** | -30.90*** | -87.33*** | -99.91*** | -8.03*** | 0.06*** | -0.12*** | 12.33 | 8.76 |
|  |  | Hot weather |  | -7.55* | -20.93*** | -29.43*** | -85.39*** | -99.16*** | -8.07 | 0.08 | 0.01 | 13.43 | 8.85 |
|  |  | Park |  | -8.26* | -20.27*** | -22.69*** | -72.23*** | -91.51*** | -7.62* | 0.32 | -0.87*** | 12.74 | 8.74 |
|  |  | Pool |  | -7.61*** | -20.49*** | -29.66*** | -82.74*** | -96.22*** | -7.24*** | 0.26*** | -0.09*** | 12.02 | 8.71 |
|  |  | Swim |  | -6.97 | -22.09*** | -29.33*** | -83.73*** | -98.34*** | -6.80 | 0.35 | -0.28** | 13.38 | 8.82 |
|  |  | Tired |  | -6.94 | -20.13*** | -28.54*** | -85.03*** | -98.50*** | -7.98 | -0.15*** | 0.08 | 12.96 | 8.83 |
|  |  | water |  | -7.90* | -20.79*** | -29.39*** | -86.12*** | -98.98*** | -7.64* | 0.31 | -0.37*** | 13.42 | 8.83 |
| Leon | None | | ARIMA(1,0,1) | - | - | - | - | - | - | - | - | 8.28 | 1242.76 |
|  |  |  |  | -1.91 | -5.86*** | -5.02*** | -19.88*** | -17.39*** | -2.67 | - | - | 5.60 | 1142.67 |
|  |  |  |  | -1.89 | -5.73*** | -5.01*** | -19.80*** | -17.44*** | -2.76 | -0.14*** | - | 5.54 | 1137.98 |
|  | Twitter | Heat |  | -2.40 | -6.31*** | -5.82*** | -19.97*** | -17.61*** | -3.51** | -0.25*** | 0.53*** | 5.40 | 1130.02 |
|  |  | AC |  | -1.89 | -5.75*** | -5.00*** | -19.81*** | -17.48*** | -2.77 | -0.14*** | -0.08 | 5.54 | 1139.95 |
|  | Google Search | AC repair |  | -1.81 | -5.38*** | -5.00*** | -19.59*** | -17.44*** | -2.68 | -0.12*** | -0.02 | 5.56 | 1138.85 |
|  |  | Beer |  | -1.75 | -5.20*** | -4.43*** | -18.97*** | -17.04*** | -2.73 | -0.15*** | -0.05* | 5.53 | 1137.39 |
|  |  | Drink |  | -1.84 | -5.70*** | -4.95*** | -19.63*** | -17.26*** | -2.78 | -0.13*** | -0.01 | 5.54 | 1139.84 |
|  |  | Heat exhaustion |  | -1.65 | -5.73*** | -5.00*** | -19.80*** | -17.29*** | -2.47 | -0.14*** | -0.04 | 5.54 | 1138.84 |
|  |  | Heat stroke |  | -1.89 | -5.73*** | -5.01*** | -19.80*** | -17.44*** | -2.76 | -0.14*** | 0.00 | 5.54 | 1139.98 |
|  |  | Hot weather |  | -1.69 | -5.48*** | -4.74*** | -19.70*** | -17.45*** | -2.62 | -0.12*** | -0.03 | 5.53 | 1138.82 |
|  |  | Park |  | -1.87 | -5.67*** | -4.96*** | -19.76*** | -17.46*** | -2.74 | -0.13*** | -0.01 | 5.55 | 1139.88 |
|  |  | Pool |  | -1.89 | -5.74*** | -5.01*** | -19.82*** | -17.45*** | -2.76 | -0.14*** | 0.00 | 5.54 | 1139.97 |
|  |  | Swim |  | -1.91 | -5.79*** | -5.04*** | -19.84*** | -17.47*** | -2.79 | -0.15*** | 0.00 | 5.54 | 1139.93 |
|  |  | Tired |  | -1.92 | -5.67*** | -4.90*** | -19.84*** | -17.38*** | -2.63 | -0.13*** | 0.03 | 5.54 | 1139.08 |
|  |  | water |  | -1.80 | -5.77*** | -5.11*** | -19.93*** | -17.48*** | -2.71 | -0.12*** | -0.03 | 5.56 | 1139.23 |
| Miami-Dade | None | | ARIMA(1,0,1) | - | - | - | - | - | - | - | - | 66.08 | 1935.90 |
|  |  |  |  | -14.29* | -41.69*** | -57.96*** | -185.49*** | -213.03*** | -9.03 | - | - | 21.35 | 1643.91 |
|  |  |  |  | -14.25* | -41.81*** | -58.13*** | -185.82*** | -213.03*** | -8.94 | 0.38 | - | 21.35 | 1645.75 |
|  | Twitter | Heat |  | -14.87** | -41.98*** | -59.94*** | -185.76*** | -213.17*** | -9.99 | 0.14 | 0.25** | 20.99 | 1644.73 |
|  |  | AC |  | -14.52** | -40.69*** | -58.90*** | -184.35*** | -212.04*** | -9.81 | -0.03 | 0.85* | 21.30 | 1645.63 |
|  | Google Search | AC repair |  | -14.13* | -41.46*** | -57.76*** | -184.98*** | -211.29*** | -8.71 | 0.13 | 0.16 | 21.40 | 1646.34 |
|  |  | Beer |  | -13.81* | -41.22*** | -53.29*** | -176.63*** | -205.13*** | -8.83 | 0.67 | -0.48*** | 21.45 | 1643.04 |
|  |  | Drink |  | -10.55 | -38.71*** | -52.89*** | -176.19*** | -203.44*** | -7.65 | 0.30 | -0.54*** | 21.81 | 1642.55 |
|  |  | Heat exhaustion |  | -14.18* | -41.76*** | -58.13*** | -185.81*** | -212.91*** | -8.87 | 0.40 | -0.02 | 21.37 | 1647.72 |
|  |  | Heat stroke |  | -16.27** | -43.10*** | -60.27*** | -188.01*** | -211.30*** | -5.89 | 0.82 | -0.38*** | 21.45 | 1635.48 |
|  |  | Hot weather |  | -14.50* | -41.59*** | -57.84*** | -185.51*** | -212.44*** | -8.21 | 0.46 | -0.09 | 21.56 | 1646.82 |
|  |  | Park |  | -12.95* | -39.77*** | -48.18*** | -166.46*** | -197.14*** | -2.09 | 1.59* | -1.27*** | 21.57 | 1633.47 |
|  |  | Pool |  | -15.03** | -42.09*** | -57.78*** | -181.05*** | -205.04*** | -6.35 | 1.54* | -0.66*** | 21.77 | 1639.73 |
|  |  | Swim |  | -14.50* | -41.89*** | -57.76*** | -185.96*** | -212.88*** | -9.36 | 0.17 | 0.14 | 21.32 | 1646.92 |
|  |  | Tired |  | -14.29* | -40.96*** | -57.16*** | -186.25*** | -213.02*** | -8.01 | 0.28 | 0.17 | 21.36 | 1647.03 |
|  |  | water |  | -13.47* | -42.24*** | -56.96*** | -184.60*** | -209.81*** | -6.22 | 1.19 | -0.68*** | 21.52 | 1643.60 |
| Orange | None | | ARMA(1,1)-GARCH(1,1) | - | - | - | - | - | - | - | - | 28.91 | 10.04 |
|  |  |  |  | -13.17*** | -21.30*** | -36.00*** | -85.48*** | -86.64*** | -3.77 | - | - | 12.79 | 8.75 |
|  |  |  |  | -13.24*** | -21.37*** | -36.08*** | -85.58*** | -86.77*** | -3.88 | -0.05 | - | 12.80 | 8.76 |
|  | Twitter | Heat |  | -13.21*** | -21.41*** | -36.08*** | -85.55*** | -86.77*** | -3.87 | 0.00 | -0.05 | 12.81 | 8.77 |
|  |  | AC |  | -13.20*** | -21.38*** | -36.08*** | -85.53*** | -86.73*** | -3.84 | -0.03 | -0.06 | 12.79 | 8.77 |
|  | Google Search | AC repair |  | -13.15*** | -21.42*** | -35.80*** | -85.45*** | -86.55*** | -3.78 | -0.08 | 0.02 | 12.80 | 8.77 |
|  |  | Beer |  | -12.65*** | -20.61*** | -30.99*** | -70.95*** | -73.71*** | -4.24 | 0.24 | -0.53*** | 12.68 | 8.69 |
|  |  | Drink |  | -11.91*** | -19.87*** | -33.64*** | -77.50*** | -81.14*** | -3.61 | 0.32 | -0.36*** | 12.68 | 8.73 |
|  |  | Heat exhaustion |  | -13.22*** | -21.37*** | -36.07*** | -85.57*** | -86.76*** | -3.87 | -0.05 | 0.00 | 12.80 | 8.77 |
|  |  | Heat stroke |  | -13.61*** | -22.02*** | -35.98*** | -85.61*** | -87.24*** | -3.97 | -0.18 | 0.08 | 12.74 | 8.76 |
|  |  | Hot weather |  | -13.33*** | -21.23*** | -36.02*** | -85.85*** | -86.80*** | -3.79 | -0.07 | 0.03 | 12.75 | 8.77 |
|  |  | Park |  | -13.27*** | -19.77*** | -31.54*** | -74.40*** | -82.15*** | -2.20 | 0.34 | -0.72*** | 12.47 | 8.69 |
|  |  | Pool |  | -13.28*** | -20.82*** | -35.30*** | -80.82*** | -81.47*** | -2.36 | 0.49* | -0.34*** | 12.59 | 8.72 |
|  |  | Swim |  | -13.53*** | -21.82*** | -36.27*** | -84.37*** | -85.69*** | -3.78 | 0.28 | -0.18** | 12.85 | 8.75 |
|  |  | Tired |  | -13.24*** | -21.16*** | -35.85*** | -85.35*** | -86.65*** | -3.68 | -0.07 | 0.02 | 12.79 | 8.77 |
|  |  | water |  | -13.58*** | -21.75*** | -36.26*** | -84.83*** | -84.88*** | -1.94 | 0.32 | -0.34** | 12.79 | 8.75 |

Table S17. Cardiovascular illness ED model specifications (*** <0.05, ** 0.05 ~ 0.10, * 0.10 ~ 0.15)

|  |  |  | Lag 0 | | | Lag 1 | | | Lag 2 | | | Lag 3 | | |
| --- | --- | --- | --- | --- | --- | --- | --- | --- | --- | --- | --- | --- | --- | --- |
|  | Keywords | | keyword | MAE | AIC | keyword | MAE | AIC | keyword | MAE | AIC | keyword | MAE | AIC |
| Duval | Twitter | Heat | -0.12 | 13.39 | 8.64 | -0.24 | 13.37 | 8.64 | 0.20 | 13.40 | 8.64 | -0.12 | 13.36 | 8.64 |
|  |  | AC | 0.31 | 13.41 | 8.64 | -1.11 | 13.34 | 8.64 | -0.68 | 13.38 | 8.64 | -0.33 | 13.36 | 8.64 |
|  | Google Search | AC repair | 0.09 | 13.31 | 8.63 | -0.06 | 13.31 | 8.64 | -0.05 | 13.37 | 8.64 | 0.10* | 13.33 | 8.63 |
|  |  | Beer | -0.13 | 13.28 | 8.63 | 0.03 | 13.38 | 8.64 | -0.19*** | 13.07 | 8.62 | 0.10 | 13.38 | 8.64 |
|  |  | Drink | -0.07 | 13.40 | 8.64 | 0.00 | 13.38 | 8.64 | 0.03 | 13.37 | 8.64 | -0.08 | 13.23 | 8.64 |
|  |  | Heat exhaustion | -0.09* | 13.37 | 8.63 | -0.11*** | 13.22 | 8.62 | 0.02 | 13.37 | 8.64 | 0.03 | 13.41 | 8.64 |
|  |  | Heat stroke | 0.09 | 13.37 | 8.64 | 0.11 | 13.40 | 8.63 | -0.08 | 13.30 | 8.64 | -0.12* | 13.15 | 8.63 |
|  |  | Hot weather | -0.01 | 13.38 | 8.64 | 0.08 | 13.39 | 8.64 | 0.10* | 13.32 | 8.63 | 0.05 | 13.40 | 8.64 |
|  |  | Park | 0.08 | 13.38 | 8.64 | -0.64*** | 13.30 | 8.64 | -0.48*** | 13.51 | 8.69 | -0.49*** | 13.33 | 8.69 |
|  |  | Pool | -0.36*** | 13.46 | 8.64 | -0.28*** | 13.49 | 8.69 | -0.38*** | 12.80 | 8.62 | -0.33*** | 13.32 | 8.66 |
|  |  | Swim | 0.07 | 13.32 | 8.64 | 0.00 | 13.40 | 8.65 | -0.01 | 13.39 | 8.64 | 0.02 | 13.39 | 8.64 |
|  |  | Tired | 0.09 | 13.30 | 8.63 | 0.06 | 13.35 | 8.64 | 0.10* | 13.08 | 8.63 | 0.01 | 13.36 | 8.64 |
|  |  | water | 0.04 | 13.39 | 8.64 | 0.08 | 13.31 | 8.64 | 0.15 | 13.37 | 8.64 | 0.01 | 13.39 | 8.64 |
| Hillsborough | Twitter | Heat | -0.44* | 14.14 | 8.67 | -0.37 | 14.13 | 8.67 | -0.39 | 14.11 | 8.67 | -0.06 | 14.13 | 8.68 |
|  |  | AC | -0.21 | 14.09 | 8.68 | -0.58 | 14.11 | 8.68 | -0.81 | 14.08 | 8.67 | 1.09** | 13.95 | 8.67 |
|  | Google Search | AC repair | -0.06 | 14.17 | 8.68 | 0.23*** | 13.64 | 8.64 | 0.03 | 14.12 | 8.68 | 0.08 | 14.04 | 8.68 |
|  |  | Beer | -0.04 | 14.13 | 8.68 | 0.12 | 14.09 | 8.68 | 0.11 | 14.00 | 8.68 | 0.13 | 14.08 | 8.68 |
|  |  | Drink | -0.32*** | 13.68 | 8.64 | -0.26*** | 13.76 | 8.65 | 0.07 | 14.10 | 8.68 | -0.05 | 14.11 | 8.68 |
|  |  | Heat exhaustion | -0.09 | 13.94 | 8.67 | 0.03 | 14.12 | 8.68 | 0.03 | 14.12 | 8.68 | -0.18*** | 13.73 | 8.64 |
|  |  | Heat stroke | 0.08 | 14.05 | 8.67 | 0.05 | 14.15 | 8.68 | 0.02 | 14.14 | 8.68 | 0.06 | 14.10 | 8.68 |
|  |  | Hot weather | -0.08 | 14.15 | 8.68 | -0.02 | 14.15 | 8.68 | -0.01 | 14.13 | 8.68 | 0.00 | 14.13 | 8.68 |
|  |  | Park | -0.31** | 13.99 | 8.66 | -0.19 | 14.00 | 8.67 | -0.08 | 14.10 | 8.68 | -0.04 | 14.11 | 8.68 |
|  |  | Pool | -0.01 | 14.12 | 8.68 | -0.01 | 14.12 | 8.68 | -0.09 | 14.00 | 8.68 | -0.05 | 14.06 | 8.68 |
|  |  | Swim | -0.01 | 14.12 | 8.68 | 0.02 | 14.13 | 8.68 | -0.02 | 14.11 | 8.68 | -0.02 | 14.12 | 8.68 |
|  |  | Tired | 0.04 | 14.05 | 8.68 | 0.15*** | 13.82 | 8.66 | 0.02 | 14.15 | 8.68 | 0.14** | 13.86 | 8.66 |
|  |  | water | 0.14 | 14.04 | 8.68 | -0.07 | 14.11 | 8.68 | -0.01 | 14.13 | 8.68 | 0.15 | 14.16 | 8.68 |
| Leon | Twitter | Heat | 0.00 | 8.01 | 7.77 | -0.38 | 7.91 | 7.76 | -0.18 | 7.99 | 7.76 | -0.04 | 8.01 | 7.77 |
|  |  | AC | 0.32 | 8.02 | 7.77 | -0.72 | 7.99 | 7.76 | - | - | - | -0.41 | 7.97 | 7.77 |
|  | Google Search | AC repair | 0.03 | 7.97 | 7.76 | 0.00 | 8.53 | 7.83 | -0.03 | 8.50 | 7.82 | 0.04 | 8.05 | 7.76 |
|  |  | Beer | -0.03 | 7.98 | 7.76 | -0.01 | 8.53 | 7.85 | -0.01 | 8.00 | 7.77 | -0.05 | 8.56 | 7.82 |
|  |  | Drink | -0.06 | 8.54 | 7.82 | -0.06 | 8.08 | 7.76 | 0.07 | 8.07 | 7.76 | 0.17*** | 7.90 | 7.71 |
|  |  | Heat exhaustion | -0.05 | 8.04 | 7.76 | 0.00 | 8.01 | 7.77 | 0.07 | 8.07 | 7.76 | 0.02 | 8.00 | 7.77 |
|  |  | Heat stroke | 0.02 | 8.51 | 7.83 | 0.06 | 7.99 | 7.76 | - | - | - | -0.01 | 8.01 | 7.77 |
|  |  | Hot weather | -0.02 | 8.00 | 7.77 | 0.04 | 7.93 | 7.76 | 0.00 | 8.53 | 7.83 | -0.06* | 8.43 | 7.82 |
|  |  | Park | 0.00 | 8.01 | 7.77 | -0.20*** | 8.08 | 7.73 | 0.02*** | 8.29 | 7.72 | -0.14*** | 8.41 | 7.81 |
|  |  | Pool | - | - | - | 0.06 | 7.97 | 7.76 | -0.07* | 8.51 | 7.82 | 0.02 | 7.99 | 7.77 |
|  |  | Swim | -0.03 | 8.00 | 7.76 | 0.04 | 7.92 | 7.76 | 0.04 | 7.94 | 7.76 | -0.08** | 7.98 | 7.75 |
|  |  | Tired | - | - | - | -0.04 | 8.55 | 7.82 | -0.03 | 7.97 | 7.76 | 0.05 | 8.03 | 7.76 |
|  |  | water | - | - | - | -0.04 | 8.03 | 7.76 | -0.02*** | 8.14 | 7.70 | 0.22*** | 7.97 | 7.70 |
| Miami-Dade | Twitter | Heat | 0.08 | 23.40 | 9.77 | 0.00 | 23.41 | 9.77 | -0.08 | 23.41 | 9.77 | 0.08 | 23.32 | 9.77 |
|  |  | AC | 0.72* | 23.40 | 9.76 | 0.20 | 23.40 | 9.77 | -0.70 | 23.16 | 9.76 | 0.27 | 23.43 | 9.77 |
|  | Google Search | AC repair | 0.28*** | 23.04 | 9.74 | -0.30*** | 22.95 | 9.73 | -0.11 | 23.20 | 9.77 | 0.20** | 23.20 | 9.75 |
|  |  | Beer | -0.19 | 23.45 | 9.77 | 0.09 | 23.41 | 9.77 | 0.09 | 23.35 | 9.77 | 0.01 | 23.41 | 9.77 |
|  |  | Drink | -0.29* | 23.38 | 9.77 | 0.21 | 23.38 | 9.76 | -0.27 | 23.35 | 9.76 | 0.11 | 23.37 | 9.77 |
|  |  | Heat exhaustion | -0.10 | 23.27 | 9.76 | 0.01 | 23.40 | 9.77 | -0.03 | 23.44 | 9.77 | -0.02 | 23.40 | 9.77 |
|  |  | Heat stroke | -0.13 | 23.58 | 9.77 | 0.00 | 23.41 | 9.77 | -0.07 | 23.29 | 9.77 | 0.06 | 23.32 | 9.77 |
|  |  | Hot weather | -0.12 | 23.24 | 9.76 | 0.15** | 23.23 | 9.75 | 0.11 | 23.46 | 9.77 | -0.07 | 23.44 | 9.77 |
|  |  | Park | -0.86*** | 23.31 | 9.73 | -0.03 | 23.41 | 9.77 | 0.39 | 23.31 | 9.76 | 0.12 | 23.40 | 9.77 |
|  |  | Pool | -0.34** | 23.19 | 9.76 | 0.04 | 23.45 | 9.77 | 0.01 | 23.41 | 9.77 | 0.20 | 23.39 | 9.77 |
|  |  | Swim | 0.06 | 23.45 | 9.77 | 0.09 | 23.46 | 9.77 | -0.05 | 23.35 | 9.77 | -0.06 | 23.30 | 9.77 |
|  |  | Tired | 0.02 | 23.41 | 9.77 | 0.17 | 23.34 | 9.76 | 0.00 | 23.28 | 9.75 | -0.12 | 23.33 | 9.77 |
|  |  | Water | -0.27 | 23.37 | 9.77 | 0.00 | 23.42 | 9.77 | 0.28 | 23.43 | 9.77 | 0.46* | 23.35 | 9.76 |
| Orange | Twitter | Heat | 0.10 | 12.64 | 8.54 | -0.03 | 12.66 | 8.54 | 0.11 | 12.68 | 8.54 | 0.16 | 12.69 | 8.54 |
|  |  | AC | -0.01 | 12.66 | 8.54 | -0.86** | 12.75 | 8.52 | -0.63 | 12.67 | 8.53 | 0.41 | 12.64 | 8.54 |
|  | Google Search | AC repair | 0.21*** | 12.75 | 8.52 | -0.06 | 12.59 | 8.54 | -0.09 | 12.64 | 8.53 | 0.09 | 12.59 | 8.53 |
|  |  | Beer | -0.29*** | 12.58 | 8.51 | -0.12 | 12.66 | 8.54 | -0.21** | 12.63 | 8.53 | -0.03 | 12.66 | 8.54 |
|  |  | Drink | 0.02 | 12.65 | 8.54 | -0.20** | 12.65 | 8.52 | -0.28*** | 12.61 | 8.50 | -0.23*** | 12.51 | 8.51 |
|  |  | Heat exhaustion | 0.01 | 12.65 | 8.54 | -0.01 | 12.65 | 8.54 | -0.09* | 12.50 | 8.53 | 0.03 | 12.68 | 8.54 |
|  |  | Heat stroke | 0.01 | 12.65 | 8.54 | -0.10* | 12.59 | 8.53 | -0.13*** | 12.46 | 8.51 | -0.03 | 12.68 | 8.54 |
|  |  | Hot weather | 0.06 | 12.57 | 8.53 | 0.13*** | 12.67 | 8.51 | 0.02 | 12.65 | 8.54 | 0.03 | 12.63 | 8.54 |
|  |  | Park | -0.27** | 12.64 | 8.52 | -0.26** | 12.65 | 8.53 | -0.24* | 12.65 | 8.53 | 0.03 | 12.65 | 8.54 |
|  |  | Pool | 0.00 | 12.66 | 8.54 | -0.03 | 12.67 | 8.54 | -0.14* | 12.63 | 8.53 | -0.08 | 12.61 | 8.54 |
|  |  | Swim | -0.01 | 12.66 | 8.54 | -0.06 | 12.67 | 8.54 | -0.05 | 12.62 | 8.54 | -0.02 | 12.66 | 8.54 |
|  |  | Tired | -0.01 | 12.66 | 8.54 | 0.01 | 12.66 | 8.54 | 0.01 | 12.66 | 8.54 | -0.08 | 12.61 | 8.54 |
|  |  | water | -0.03 | 12.67 | 8.54 | -0.16 | 12.63 | 8.54 | -0.09 | 12.64 | 8.54 | -0.21 | 12.61 | 8.53 |

Table S18. Cardiovascular illness hospitalization model specifications (*** <0.05, ** 0.05 ~ 0.10, * 0.10 ~ 0.15)

|  |  |  | Lag 0 | | | Lag 1 | | | Lag 2 | | | Lag 3 | | |
| --- | --- | --- | --- | --- | --- | --- | --- | --- | --- | --- | --- | --- | --- | --- |
|  | Keywords | | keyword | MAE | AIC | keyword | MAE | AIC | keyword | MAE | AIC | keyword | MAE | AIC |
| Duval | Twitter | Heat | 0.05 | 13.37 | 8.78 | -0.69* | 13.25 | 8.77 | -0.71** | 13.27 | 8.76 | -0.53 | 13.40 | 8.77 |
|  |  | AC | 1.34 | 13.29 | 8.77 | -0.83 | 13.37 | 8.77 | -0.15 | 13.34 | 8.78 | -0.95 | 13.37 | 8.77 |
|  | Google Search | AC repair | 0.01 | 13.37 | 8.78 | -0.04 | 13.33 | 8.78 | -0.08 | 13.31 | 8.77 | 0.00 | 13.35 | 8.78 |
|  |  | Beer | -0.26*** | 13.28 | 8.73 | 0.09 | 13.41 | 8.77 | -0.01 | 13.35 | 8.78 | -0.06 | 13.31 | 8.78 |
|  |  | Drink | -0.12* | 13.34 | 8.76 | -0.09 | 13.23 | 8.77 | -0.07 | 13.23 | 8.77 | 0.00 | 13.36 | 8.78 |
|  |  | Heat exhaustion | 0.01 | 13.35 | 8.78 | -0.17*** | 13.34 | 8.73 | 0.00 | 13.36 | 8.78 | -0.02 | 13.36 | 8.78 |
|  |  | Heat stroke | -0.06 | 13.30 | 8.78 | -0.13* | 13.16 | 8.77 | -0.02 | 13.32 | 8.78 | -0.17*** | 13.23 | 8.76 |
|  |  | Hot weather | -0.06 | 13.35 | 8.77 | 0.03 | 13.36 | 8.78 | -0.10* | 13.38 | 8.76 | 0.03 | 13.39 | 8.78 |
|  |  | Park | -0.36*** | 13.18 | 8.73 | -0.24** | 13.28 | 8.76 | -0.29*** | 13.16 | 8.75 | -0.12 | 13.24 | 8.77 |
|  |  | Pool | -0.12* | 13.22 | 8.76 | -0.06 | 13.39 | 8.77 | -0.06 | 13.35 | 8.77 | -0.20*** | 13.03 | 8.74 |
|  |  | Swim | -0.03 | 13.33 | 8.78 | -0.02 | 13.35 | 8.78 | -0.03 | 13.34 | 8.78 | -0.01 | 13.33 | 8.78 |
|  |  | Tired | -0.08 | 13.19 | 8.77 | -0.05 | 13.29 | 8.77 | 0.09 | 13.30 | 8.77 | 0.00 | 13.35 | 8.78 |
|  |  | water | -0.35*** | 13.05 | 8.74 | -0.29*** | 13.18 | 8.75 | -0.18 | 13.38 | 8.77 | 0.03 | 13.34 | 8.78 |
| Hillsborough | Twitter | Heat | 0.25 | 13.35 | 8.85 | 0.41 | 13.45 | 8.85 | -0.25 | 13.40 | 8.85 | -0.99*** | 12.00 | 8.71 |
|  |  | AC | -0.56*** | 12.32 | 8.77 | 0.32 | 13.40 | 8.85 | 1.72*** | 13.12 | 8.83 | - | - | - |
|  | Google Search | AC repair | -0.07 | 13.42 | 8.85 | 0.01 | 13.04 | 8.83 | 0.02 | 13.40 | 8.85 | 0.03 | 13.42 | 8.85 |
|  |  | Beer | -0.23** | 12.97 | 8.81 | - | - | - | - | - | - | - | - | - |
|  |  | Drink | -0.26*** | 12.02 | 8.69 | - | - | - | - | - | - | 0.01 | 13.42 | 8.85 |
|  |  | Heat exhaustion | -0.02 | 13.41 | 8.85 | -0.04 | 13.00 | 8.83 | - | - | - | -0.09 | 13.24 | 8.83 |
|  |  | Heat stroke | -0.12*** | 12.33 | 8.76 | -0.01 | 13.42 | 8.85 | -0.09*** | 13.18 | 8.83 | -0.03 | 13.37 | 8.85 |
|  |  | Hot weather | 0.01 | 13.43 | 8.85 | -0.20*** | 12.31 | 8.74 | -0.09 | 13.50 | 8.85 | -0.12 | 12.98 | 8.82 |
|  |  | Park | -0.87*** | 12.74 | 8.74 | - | - | - | -0.26*** | 12.24 | 8.73 | - | - | - |
|  |  | Pool | -0.09*** | 12.02 | 8.71 | -0.11*** | 11.98 | 8.71 | - | - | - | - | - | - |
|  |  | Swim | -0.28** | 13.38 | 8.82 | -0.13*** | 11.96 | 8.71 | 0.08 | 13.44 | 8.85 | - | - | - |
|  |  | Tired | 0.08 | 12.96 | 8.83 | 0.09 | 13.38 | 8.84 | 0.04 | 13.02 | 8.83 | - | - | - |
|  |  | water | -0.37*** | 13.42 | 8.83 | 0.00 | 13.42 | 8.85 | -0.28*** | 11.85 | 8.70 | 0.03 | 13.42 | 8.85 |
| Leon | Twitter | Heat | 0.53*** | 5.40 | 1130.02 | 0.08 | 5.53 | 1139.74 | -0.06 | 5.54 | 1139.87 | 0.04 | 5.53 | 1139.93 |
|  |  | AC | -0.08 | 5.54 | 1139.95 | -0.17 | 5.54 | 1139.87 | -0.09 | 5.53 | 1139.94 | 0.14 | 5.54 | 1139.88 |
|  | Google Search | AC repair | -0.02 | 5.56 | 1138.85 | 0.02 | 5.49 | 1139.05 | -0.04** | 5.54 | 1137.03 | 0.02 | 5.51 | 1139.00 |
|  |  | Beer | -0.05* | 5.53 | 1137.39 | -0.02 | 5.57 | 1139.52 | -0.01 | 5.55 | 1139.87 | -0.04 | 5.49 | 1138.38 |
|  |  | Drink | -0.01 | 5.54 | 1139.84 | -0.01 | 5.55 | 1139.87 | 0.04 | 5.50 | 1138.99 | -0.01 | 5.54 | 1139.88 |
|  |  | Heat exhaustion | -0.04 | 5.54 | 1138.84 | -0.05 | 5.50 | 1138.39 | 0.03 | 5.53 | 1139.52 | 0.00 | 5.54 | 1139.97 |
|  |  | Heat stroke | 0.00 | 5.54 | 1139.98 | -0.03 | 5.52 | 1138.93 | 0.02 | 5.52 | 1139.56 | -0.04 | 5.54 | 1138.66 |
|  |  | Hot weather | -0.03 | 5.53 | 1138.82 | 0.03 | 5.53 | 1139.21 | 0.00 | 5.54 | 1139.98 | -0.04 | 5.55 | 1138.04 |
|  |  | Park | -0.01 | 5.55 | 1139.88 | 0.00 | 5.54 | 1139.98 | 0.01 | 5.53 | 1139.86 | 0.02 | 5.50 | 1139.37 |
|  |  | Pool | 0.00 | 5.54 | 1139.97 | 0.01 | 5.52 | 1139.62 | -0.01 | 5.55 | 1139.77 | -0.02 | 5.55 | 1138.98 |
|  |  | Swim | 0.00 | 5.54 | 1139.93 | -0.01 | 5.54 | 1139.82 | -0.01 | 5.55 | 1139.82 | -0.01 | 5.54 | 1139.87 |
|  |  | Tired | 0.03 | 5.54 | 1139.08 | 0.02 | 5.50 | 1139.48 | 0.02 | 5.52 | 1139.70 | 0.00 | 5.54 | 1139.96 |
|  |  | water | -0.03 | 5.56 | 1139.23 | 0.01 | 5.54 | 1139.91 | -0.04 | 5.56 | 1138.59 | -0.05* | 5.48 | 1137.59 |
| Miami-Dade | Twitter | Heat | 0.25** | 20.99 | 1644.73 | -0.03 | 21.37 | 1647.69 | -0.08 | 21.35 | 1647.46 | -0.02 | 21.38 | 1647.72 |
|  |  | AC | 0.85* | 21.30 | 1645.63 | -0.16 | 21.30 | 1647.67 | 0.68 | 21.05 | 1646.58 | -0.18 | 21.38 | 1647.64 |
|  | Google Search | AC repair | 0.16 | 21.40 | 1646.34 | -0.14 | 21.33 | 1646.61 | -0.32*** | 20.80 | 1642.02 | -0.02 | 21.33 | 1647.73 |
|  |  | Beer | -0.48*** | 21.45 | 1643.04 | -0.29 | 21.41 | 1646.06 | 0.01 | 21.35 | 1647.75 | -0.09 | 21.42 | 1647.58 |
|  |  | Drink | -0.54*** | 21.81 | 1642.55 | 0.72*** | 21.81 | 1638.21 | -0.49*** | 21.85 | 1643.93 | 0.34* | 21.45 | 1645.88 |
|  |  | Heat exhaustion | -0.02 | 21.37 | 1647.72 | 0.04 | 21.38 | 1647.62 | -0.25*** | 21.63 | 1643.32 | 0.18* | 21.00 | 1645.22 |
|  |  | Heat stroke | -0.38*** | 21.45 | 1635.48 | -0.09 | 21.17 | 1647.05 | 0.02 | 21.34 | 1647.73 | 0.11 | 21.49 | 1646.87 |
|  |  | Hot weather | -0.09 | 21.56 | 1646.82 | 0.05 | 21.35 | 1647.53 | -0.16** | 20.68 | 1644.94 | -0.04 | 21.41 | 1647.54 |
|  |  | Park | -1.27*** | 21.57 | 1633.47 | -0.64*** | 21.43 | 1643.52 | -0.41 | 21.48 | 1646.23 | -0.15 | 21.43 | 1647.82 |
|  |  | Pool | -0.66*** | 21.77 | 1639.73 | -0.46*** | 21.60 | 1643.46 | -0.24 | 21.52 | 1646.94 | 0.09 | 21.31 | 1647.61 |
|  |  | Swim | 0.14 | 21.32 | 1646.92 | -0.21 | 21.67 | 1645.95 | -0.12 | 21.58 | 1647.16 | 0.08 | 21.36 | 1647.46 |
|  |  | Tired | 0.17 | 21.36 | 1647.03 | 0.36** | 21.09 | 1644.59 | -0.09 | 21.32 | 1647.53 | 0.20 | 21.48 | 1647.01 |
|  |  | Water | -0.68*** | 21.52 | 1643.60 | -0.29 | 21.26 | 1647.04 | -0.29 | 21.64 | 1647.35 | -0.21 | 21.32 | 1647.37 |
| Orange | Twitter | Heat | -0.05 | 12.81 | 8.77 | -0.32* | 12.99 | 8.76 | -0.20 | 12.87 | 8.77 | -0.03 | 12.81 | 8.77 |
|  |  | AC | -0.06 | 12.79 | 8.77 | 0.49 | 12.71 | 8.77 | -0.90* | 12.92 | 8.76 | -1.41*** | 12.79 | 8.74 |
|  | Google Search | AC repair | 0.02 | 12.80 | 8.77 | 0.02 | 12.78 | 8.77 | -0.13** | 12.93 | 8.75 | -0.08 | 12.77 | 8.77 |
|  |  | Beer | -0.53*** | 12.68 | 8.69 | -0.42*** | 12.82 | 8.72 | -0.28** | 12.82 | 8.75 | -0.11 | 12.83 | 8.77 |
|  |  | Drink | -0.36*** | 12.68 | 8.73 | -0.30*** | 12.72 | 8.74 | -0.12 | 12.75 | 8.77 | -0.35*** | 12.44 | 8.73 |
|  |  | Heat exhaustion | 0.00 | 12.80 | 8.77 | 0.00 | 12.80 | 8.77 | -0.11** | 12.62 | 8.75 | -0.05 | 12.95 | 8.77 |
|  |  | Heat stroke | 0.08 | 12.74 | 8.76 | -0.15*** | 12.69 | 8.75 | 0.13** | 12.61 | 8.75 | 0.00 | 12.79 | 8.77 |
|  |  | Hot weather | 0.03 | 12.75 | 8.77 | 0.00 | 12.79 | 8.77 | -0.09 | 12.86 | 8.76 | -0.06 | 12.89 | 8.77 |
|  |  | Park | -0.72*** | 12.47 | 8.69 | -0.47*** | 12.76 | 8.74 | -0.29* | 12.87 | 8.76 | -0.38*** | 12.61 | 8.75 |
|  |  | Pool | -0.34*** | 12.59 | 8.72 | -0.15 | 12.86 | 8.76 | -0.32*** | 12.49 | 8.73 | -0.20** | 12.76 | 8.75 |
|  |  | Swim | -0.18** | 12.85 | 8.75 | -0.21*** | 12.64 | 8.74 | -0.18*** | 12.62 | 8.75 | -0.11 | 12.85 | 8.76 |
|  |  | Tired | 0.02 | 12.79 | 8.77 | -0.07 | 12.79 | 8.77 | 0.15** | 12.74 | 8.75 | -0.01 | 12.80 | 8.77 |
|  |  | water | -0.34** | 12.79 | 8.75 | -0.42*** | 12.63 | 8.74 | -0.12 | 12.79 | 8.77 | -0.46*** | 12.54 | 8.74 |

Table S19. Respiratory illness ED model specifications (*** <0.05, ** 0.05 ~ 0.10, * 0.10 ~ 0.15)

| County | Keywords | | Model | Wed | Thu | Fri | Sat | Sun | Mon | Max T | keyword | MAE | AIC |
| --- | --- | --- | --- | --- | --- | --- | --- | --- | --- | --- | --- | --- | --- |
| Duval | None | | ARMA(1,1)-GARCH(1,1) | - | - | - | - | - | - | - | - | 16.18 | 8.93 |
|  |  |  |  | -2.86 | -6.34* | -20.81*** | -25.14*** | -11.11*** | 8.15*** | - | - | 12.67 | 8.56 |
|  |  |  |  | -2.79 | -6.14* | -20.59*** | -24.90*** | -10.97*** | 8.12*** | -0.11 | - | 12.66 | 8.57 |
|  | Twitter | Heat |  | -2.87 | -6.44* | -20.89*** | -25.03*** | -11.49*** | 8.39*** | 0.05 | -0.62 | 12.67 | 8.57 |
|  |  | AC |  | -3.19 | -6.03* | -21.01*** | -24.94*** | -11.32*** | 8.13*** | -0.15 | 0.97 | 12.73 | 8.57 |
|  | Google Search | AC repair |  | -2.98 | -6.35* | -21.50*** | -25.74*** | -11.54*** | 7.20** | -0.08 | -0.07 | 12.66 | 8.57 |
|  |  | Beer |  | -2.66 | -6.81** | -19.39*** | -22.12*** | -8.91*** | 8.16*** | -0.12 | -0.14** | 12.56 | 8.56 |
|  |  | Drink |  | -3.00 | -6.10* | -20.90*** | -25.59*** | -11.92*** | 8.08*** | -0.17 | 0.06 | 12.71 | 8.57 |
|  |  | Heat exhaustion |  | -2.77 | -6.15* | -20.59*** | -24.89*** | -10.98*** | 8.12*** | -0.11 | 0.00 | 12.66 | 8.58 |
|  |  | Heat stroke |  | -2.76 | -6.14* | -20.60*** | -24.85*** | -11.00*** | 8.10*** | -0.11 | 0.01 | 12.65 | 8.58 |
|  |  | Hot weather |  | -2.79 | -6.18* | -20.49*** | -24.70*** | -10.83*** | 8.19*** | -0.10 | -0.02 | 12.63 | 8.58 |
|  |  | Park |  | -2.71 | -6.18* | -20.14*** | -24.31*** | -10.76*** | 8.41*** | -0.07 | -0.06 | 12.65 | 8.58 |
|  |  | Pool |  | -2.78 | -6.15* | -20.59*** | -24.92*** | -10.99*** | 8.12*** | -0.11 | 0.00 | 12.66 | 8.58 |
|  |  | Swim |  | -3.24 | -6.45* | -21.76*** | -24.92*** | -10.71*** | 7.96*** | -0.11 | -0.11** | 12.58 | 8.56 |
|  |  | Tired |  | -1.70 | -4.01 | -19.87*** | -23.61*** | -9.70*** | 8.44*** | -0.17 | -0.11*** | 12.64 | 8.56 |
|  |  | water |  | -3.06 | -6.42* | -20.59*** | -25.23*** | -11.32*** | 7.86*** | -0.11 | 0.07 | 12.70 | 8.58 |
| Hillsborough | None | | ARMA(2,1)-GARCH(1,1) | - | - | - | - | - | - | - | - | 21.31 | 9.43 |
|  |  |  |  | 2.34 | -14.59*** | -31.86*** | -19.34*** | -8.99** | 11.86*** | - | - | 16.46 | 8.99 |
|  |  |  |  | 1.70 | -15.18*** | -31.81*** | -19.38*** | -8.89** | 11.70*** | 0.77 | - | 16.39 | 9.00 |
|  | Twitter | Heat |  | 1.98 | -14.80*** | -31.09*** | -19.13*** | -8.75** | 12.04*** | 0.85* | -0.31 | 16.29 | 9.01 |
|  |  | AC |  | 1.77 | -15.15*** | -31.79*** | -19.44*** | -9.05** | 11.88*** | 0.77 | -0.44 | 16.34 | 9.01 |
|  | Google Search | AC repair |  | 1.41 | -15.76*** | -32.27*** | -20.35*** | -9.14** | 11.34*** | 0.64 | 0.09 | 16.33 | 9.00 |
|  |  | Beer |  | 1.65 | -14.91*** | -31.40*** | -17.92*** | -7.93 | 12.08*** | 0.82 | -0.05 | 16.40 | 9.01 |
|  |  | Drink |  | 0.23 | -15.17*** | -31.47*** | -15.62*** | -6.39 | 9.68*** | 0.94* | -0.30*** | 16.19 | 8.98 |
|  |  | Heat exhaustion |  | 1.58 | -15.58*** | -31.70*** | -19.15*** | -8.48** | 12.19*** | 0.87 | -0.05 | 16.35 | 9.01 |
|  |  | Heat stroke |  | 1.78 | -15.01*** | -31.64*** | -18.95*** | -8.56** | 12.07*** | 0.76 | 0.01 | 16.41 | 9.01 |
|  |  | Hot weather |  | 1.59 | -14.95*** | -31.59*** | -19.01*** | -8.78** | 12.08*** | 0.73 | 0.03 | 16.37 | 9.01 |
|  |  | Park |  | 1.59 | -15.51*** | -32.99*** | -21.30*** | -10.22** | 11.51*** | 0.75 | 0.12 | 16.43 | 9.01 |
|  |  | Pool |  | 1.06 | -15.55*** | -32.19*** | -17.99*** | -7.73 | 12.25*** | 0.83 | -0.12 | 16.34 | 9.01 |
|  |  | Swim |  | 2.76 | -15.22*** | -31.24*** | -17.41*** | -7.41 | 13.49*** | 0.67 | -0.20 | 16.35 | 9.00 |
|  |  | Tired |  | 1.95 | -15.17*** | -31.72*** | -18.77*** | -8.08* | 12.44*** | 0.73 | -0.07 | 16.40 | 9.00 |
|  |  | water |  | 1.98 | -15.33*** | -31.79*** | -18.60*** | -9.18** | 11.08*** | 0.68 | 0.37** | 16.23 | 8.99 |
| Leon | None | | ARMA(1,1)-GARCH(1,1) | - | - | - | - | - | - | - | - | 7.18 | 7.41 |
|  |  |  |  | -0.94 | -5.42*** | -3.35 | -6.66*** | -3.61* | 2.82 | - | - | 6.90 | 7.35 |
|  |  |  |  | -0.85 | -5.33*** | -3.18 | -6.32*** | -3.53* | 2.88 | -0.26 | - | 6.90 | 7.35 |
|  | Twitter | Heat |  | -0.90 | -5.38*** | -3.27 | -6.32*** | -3.54* | 2.77 | -0.27 | 0.08 | 6.91 | 7.36 |
|  |  | AC |  | -0.92 | -5.26*** | -3.33 | -6.31*** | -3.08 | 2.93 | -0.33* | 0.88 | 6.86 | 7.35 |
|  | Google Search | AC repair |  | -0.83 | -5.18*** | -3.20 | -6.23*** | -3.53* | 2.92 | -0.26 | -0.01 | 6.89 | 7.36 |
|  |  | Beer |  | -0.91 | -5.60*** | -3.47 | -6.75*** | -3.74* | 2.86 | -0.25 | 0.03 | 6.87 | 7.36 |
|  |  | Drink |  | -1.02 | -5.37*** | -3.41 | -7.15*** | -4.39** | 2.95 | -0.25 | 0.06 | 6.85 | 7.35 |
|  |  | Heat exhaustion |  | -0.80 | -5.33*** | -3.18 | -6.32*** | -3.50* | 2.93 | -0.25 | -0.01 | 6.90 | 7.36 |
|  |  | Heat stroke |  | -0.96 | -5.65*** | -3.14 | -6.55*** | -3.78* | 2.80 | -0.28 | 0.07** | 6.86 | 7.34 |
|  |  | Hot weather |  | -0.89 | -5.38*** | -3.24 | -6.34*** | -3.52* | 2.85 | -0.26 | 0.01 | 6.90 | 7.36 |
|  |  | Park |  | -0.90 | -4.86*** | -2.71 | -5.72*** | -3.94** | 3.30 | -0.28 | -0.14*** | 6.82 | 7.34 |
|  |  | Pool |  | -0.84 | -5.32*** | -3.12 | -6.60*** | -3.74* | 2.86 | -0.26 | 0.02 | 6.89 | 7.36 |
|  |  | Swim |  | -0.91 | -5.77*** | -3.56* | -6.71*** | -3.91* | 2.47 | -0.29* | 0.05 | 6.89 | 7.35 |
|  |  | Tired |  | -0.85 | -5.31*** | -3.15 | -6.31*** | -3.51* | 2.90 | -0.26 | 0.00 | 6.90 | 7.36 |
|  |  | water |  | -0.98 | -4.96*** | -2.78 | -5.93*** | -3.44* | 2.73 | -0.26 | 0.09** | 6.82 | 7.35 |
| Miami-Dade | None | | ARMA(1,1)-GARCH(1,1) | - | - | - | - | - | - | - | - | 29.15 | 10.12 |
|  |  |  |  | -21.85*** | -34.97*** | -39.44*** | -19.94*** | -8.60 | 28.48*** | - | - | 23.10 | 9.72 |
|  |  |  |  | -21.52*** | -34.75*** | -39.31*** | -19.96*** | -8.32 | 28.78*** | 0.40 | - | 23.10 | 9.73 |
|  | Twitter | Heat |  | -21.67*** | -34.73*** | -39.78*** | -19.85*** | -8.36 | 28.46*** | 0.28 | 0.07 | 23.07 | 9.74 |
|  |  | AC |  | -21.54*** | -35.09*** | -39.20*** | -20.39*** | -8.59 | 28.93*** | 0.48 | -0.19 | 23.04 | 9.75 |
|  | Google Search | AC repair |  | -21.55*** | -34.87*** | -39.44*** | -20.23*** | -8.85 | 28.71*** | 0.48 | -0.05 | 23.14 | 9.75 |
|  |  | Beer |  | -21.56*** | -34.82*** | -40.10*** | -21.46*** | -9.63 | 28.76*** | 0.37 | 0.08 | 23.06 | 9.75 |
|  |  | Drink |  | -20.19*** | -33.67*** | -37.44*** | -16.22*** | -4.72 | 29.28*** | 0.40 | -0.20 | 23.07 | 9.74 |
|  |  | Heat exhaustion |  | -21.28*** | -34.57*** | -39.26*** | -19.85*** | -7.90 | 29.02*** | 0.43 | -0.06 | 23.15 | 9.74 |
|  |  | Heat stroke |  | -21.88*** | -34.93*** | -39.68*** | -20.33*** | -8.00 | 29.35*** | 0.46 | -0.07 | 23.01 | 9.74 |
|  |  | Hot weather |  | -21.42*** | -34.85*** | -39.44*** | -20.08*** | -8.58 | 28.47*** | 0.37 | 0.04 | 23.12 | 9.75 |
|  |  | Park |  | -21.98*** | -35.54*** | -43.84*** | -28.82*** | -15.59** | 25.69*** | 0.06 | 0.58* | 22.87 | 9.73 |
|  |  | Pool |  | -21.51*** | -34.79*** | -39.41*** | -20.29*** | -8.78 | 28.58*** | 0.41 | 0.03 | 23.10 | 9.75 |
|  |  | Swim |  | -21.94*** | -34.90*** | -38.75*** | -20.67*** | -8.16 | 27.85*** | 0.43 | 0.29** | 22.70 | 9.73 |
|  |  | Tired |  | -21.56*** | -34.45*** | -38.99*** | -20.20*** | -8.32 | 29.16*** | 0.40 | 0.07 | 23.08 | 9.75 |
|  |  | water |  | -21.88*** | -34.52*** | -40.12*** | -20.93*** | -10.38 | 27.09*** | 0.20 | 0.40 | 23.14 | 9.74 |
| Orange | None | | ARMA(1,1)-GARCH(1,1) | - | - | - | - | - | - | - | - | 19.55 | 9.32 |
|  |  |  |  | -5.80 | -11.47*** | -26.09*** | -25.12*** | -17.30*** | 12.73*** | - | - | 16.97 | 9.10 |
|  |  |  |  | -5.73 | -11.46*** | -26.09*** | -25.00*** | -17.20*** | 12.71* | 0.02 | - | 16.96 | 9.11 |
|  | Twitter | Heat |  | -5.42 | -10.80*** | -25.61*** | -24.55*** | -16.88*** | 12.98*** | -0.26 | 0.22 | 16.74 | 9.12 |
|  |  | AC |  | -4.30 | -8.87* | -23.09*** | -22.81*** | -15.23*** | 14.60*** | -0.51 | 1.58*** | 16.41 | 9.09 |
|  | Google Search | AC repair |  | -5.67 | -11.14*** | -26.21*** | -24.96*** | -17.51*** | 12.68*** | -0.09 | -0.04 | 16.99 | 9.12 |
|  |  | Beer |  | -5.30 | -11.09*** | -24.69*** | -22.38*** | -14.89*** | 12.93*** | -0.10 | -0.08 | 16.96 | 9.12 |
|  |  | Drink |  | -4.10 | -9.94** | -24.16*** | -19.43*** | -13.17*** | 13.51*** | -0.06 | -0.23 | 16.78 | 9.11 |
|  |  | Heat exhaustion |  | -5.95 | -11.56*** | -25.70*** | -24.96*** | -17.50*** | 13.21*** | -0.24 | 0.07 | 16.86 | 9.12 |
|  |  | Heat stroke |  | -6.60 | -12.90*** | -25.82*** | -25.10*** | -18.38*** | 12.33*** | -0.25 | 0.18*** | 16.61 | 9.10 |
|  |  | Hot weather |  | -5.01 | -11.14*** | -25.63*** | -23.65*** | -16.57*** | 12.96*** | -0.16 | -0.09 | 16.81 | 9.11 |
|  |  | Park |  | -6.28 | -11.32*** | -24.35*** | -20.59*** | -15.81*** | 12.99*** | -0.14 | -0.31 | 16.85 | 9.12 |
|  |  | Pool |  | -5.93 | -12.22*** | -26.88*** | -29.55*** | -22.29*** | 11.04*** | -0.32 | 0.29 | 16.74 | 9.12 |
|  |  | Swim |  | -5.13 | -10.74*** | -25.32*** | -25.01*** | -17.30*** | 13.01*** | -0.22 | 0.10 | 16.98 | 9.12 |
|  |  | Tired |  | -5.94 | -11.11*** | -25.56*** | -24.59*** | -17.18*** | 12.97*** | -0.18 | 0.06 | 16.97 | 9.12 |
|  |  | water |  | -5.10 | -10.89*** | -25.20*** | -24.53*** | -17.10*** | 12.73*** | -0.19 | 0.09 | 16.97 | 9.12 |

Table S20. Respiratory illness hospitalization model specifications (*** <0.05, ** 0.05 ~ 0.10, * 0.10 ~ 0.15)

| County | Keywords | | Model | Wed | Thu | Fri | Sat | Sun | Mon | Max T | keyword | MAE | AIC |
| --- | --- | --- | --- | --- | --- | --- | --- | --- | --- | --- | --- | --- | --- |
| Duval | None | | ARMA(1,1)-GARCH(1,1) | - | - | - | - | - | - | - | - | 13.63 | 8.53 |
|  |  |  |  | -4.70* | -12.58*** | -12.41*** | -31.10*** | -36.66*** | 0.92 | - | - | 8.52 | 7.71 |
|  |  |  |  | -4.42* | -12.29*** | -12.14*** | -30.91*** | -36.69*** | 0.78 | -0.24* | - | 8.42 | 7.71 |
|  | Twitter | Heat |  | -4.41* | -12.29*** | -12.14*** | -30.90*** | -36.70*** | 0.80 | -0.23 | -0.03 | 8.42 | 7.72 |
|  |  | AC |  | -4.52* | -12.24*** | -12.27*** | -30.97*** | -36.76*** | 0.83 | -0.26* | 0.30 | 8.43 | 7.72 |
|  | Google Search | AC repair |  | -4.36* | -12.15*** | -11.60*** | -30.32*** | -36.33*** | 1.30 | -0.26** | 0.05 | 8.38 | 7.72 |
|  |  | Beer |  | -4.34* | -12.88*** | -10.83*** | -27.65*** | -33.79*** | 0.80 | -0.13 | -0.18*** | 8.32 | 7.66 |
|  |  | Drink |  | -4.25* | -12.28*** | -11.91*** | -29.90*** | -35.58*** | 0.67 | -0.18 | -0.08* | 8.40 | 7.71 |
|  |  | Heat exhaustion |  | -4.46* | -12.28*** | -12.14*** | -30.95*** | -36.70*** | 0.78 | -0.23* | -0.01 | 8.41 | 7.72 |
|  |  | Heat stroke |  | -4.42* | -12.28*** | -12.12*** | -30.93*** | -36.66*** | 0.82 | -0.23* | -0.01 | 8.42 | 7.72 |
|  |  | Hot weather |  | -4.54* | -12.47*** | -11.99*** | -30.50*** | -36.40*** | 0.90 | -0.23* | -0.06* | 8.40 | 7.71 |
|  |  | Park |  | -4.27* | -12.51*** | -10.32*** | -27.95*** | -35.70*** | 1.79 | -0.02 | -0.28*** | 8.10 | 7.67 |
|  |  | Pool |  | -4.58* | -12.20*** | -12.11*** | -30.16*** | -36.13*** | 0.88 | -0.18 | -0.06 | 8.42 | 7.72 |
|  |  | Swim |  | -4.70* | -12.37*** | -12.63*** | -30.85*** | -36.53*** | 0.67 | -0.19 | -0.05 | 8.44 | 7.72 |
|  |  | Tired |  | -4.25 | -12.07*** | -12.08*** | -30.78*** | -36.56*** | 0.83 | -0.24* | -0.01 | 8.42 | 7.72 |
|  |  | water |  | -3.52 | -11.36*** | -12.23*** | -29.75*** | -35.53*** | 1.71 | -0.13 | -0.25*** | 8.30 | 7.66 |
| Hillsborough | None | | ARMA(2,1)-GARCH(1,1) | - | - | - | - | - | - | - | - | 15.40 | 8.83 |
|  |  |  |  | -2.40 | -9.69*** | -14.68*** | -41.56*** | -45.89*** | -0.45 | - | - | 9.98 | 8.04 |
|  |  |  |  | -2.02 | -9.24*** | -14.69*** | -41.60*** | -46.12*** | -0.63 | -0.46*** | - | 9.77 | 8.04 |
|  | Twitter | Heat |  | -1.95 | -9.10*** | -14.32*** | -41.57*** | -46.16*** | -0.50 | -0.37** | -0.24 | 9.71 | 8.04 |
|  |  | AC |  | -1.98 | -9.24*** | -14.69*** | -41.67*** | -46.17*** | -0.60 | -0.45*** | -0.14 | 9.79 | 8.05 |
|  | Google Search | AC repair |  | -2.05 | -9.30*** | -14.73*** | -41.73*** | -46.15*** | -0.67 | -0.47*** | 0.01 | 9.76 | 8.05 |
|  |  | Beer |  | -1.70 | -7.54*** | -12.25*** | -36.39*** | -42.59*** | 0.31 | -0.38*** | -0.19*** | 9.62 | 8.02 |
|  |  | Drink |  | -2.94 | -9.27*** | -14.54*** | -39.66*** | -44.73*** | -1.92 | -0.38*** | -0.17** | 9.62 | 8.03 |
|  |  | Heat exhaustion |  | -2.16 | -9.54*** | -14.74*** | -41.69*** | -46.05*** | -0.50 | -0.41*** | -0.03 | 9.77 | 8.05 |
|  |  | Heat stroke |  | -2.01 | -8.85*** | -14.62*** | -41.15*** | -45.64*** | -0.09 | -0.48*** | 0.05 | 9.80 | 8.04 |
|  |  | Hot weather |  | -2.19 | -9.13*** | -14.63*** | -41.52*** | -46.08*** | -0.41 | -0.49*** | 0.04 | 9.79 | 8.04 |
|  |  | Park |  | -2.26 | -8.93*** | -11.71*** | -35.86*** | -42.70*** | -0.26 | -0.36*** | -0.37*** | 9.34 | 8.00 |
|  |  | Pool |  | -2.12 | -9.17*** | -14.63*** | -40.97*** | -45.54*** | -0.32 | -0.40*** | -0.05 | 9.78 | 8.05 |
|  |  | Swim |  | -1.79 | -9.64*** | -14.63*** | -40.98*** | -45.77*** | -0.13 | -0.37*** | -0.10 | 9.71 | 8.04 |
|  |  | Tired |  | -1.97 | -9.11*** | -14.54*** | -41.68*** | -46.34*** | -0.77 | -0.47*** | 0.03 | 9.80 | 8.05 |
|  |  | water |  | -2.32 | -9.21*** | -14.68*** | -42.07*** | -46.05*** | -0.32 | -0.34** | -0.20* | 9.83 | 8.05 |
| Leon | None | | ARMA(1,1)-GARCH(1,1) | - | - | - | - | - | - | - | - | 4.49 | 6.30 |
|  |  |  |  | -0.29 | -2.80*** | -2.36** | -8.97*** | -7.12*** | -0.32 | - | - | 3.57 | 5.94 |
|  |  |  |  | -0.29 | -2.80*** | -2.33** | -8.90*** | -7.12*** | -0.34 | -0.08 | - | 3.58 | 5.95 |
|  | Twitter | Heat |  | -0.33 | -2.84*** | -2.42** | -8.90*** | -7.14*** | -0.44 | -0.09 | 0.07 | 3.58 | 5.96 |
|  |  | AC |  | -0.26 | -2.82*** | -2.28** | -8.88*** | -7.24*** | -0.35 | -0.06 | -0.26 | 3.58 | 5.95 |
|  | Google Search | AC repair |  | -0.25 | -2.39** | -2.39** | -8.65*** | -7.13*** | -0.23 | -0.07 | -0.03*** | 3.48 | 5.93 |
|  |  | Beer |  | -0.22 | -2.53*** | -2.05* | -8.49*** | -6.92*** | -0.32 | -0.08 | -0.03 | 3.56 | 5.95 |
|  |  | Drink |  | -0.28 | -2.80*** | -2.32** | -8.85*** | -7.07*** | -0.34 | -0.08 | 0.00 | 3.57 | 5.96 |
|  |  | Heat exhaustion |  | -0.20 | -2.80*** | -2.33** | -8.90*** | -7.06*** | -0.23 | -0.08 | -0.02 | 3.57 | 5.96 |
|  |  | Heat stroke |  | -0.25 | -2.70*** | -2.34** | -8.83*** | -7.05*** | -0.32 | -0.07 | -0.02 | 3.56 | 5.95 |
|  |  | Hot weather |  | -0.12 | -2.60*** | -2.11** | -8.81*** | -7.13*** | -0.22 | -0.06 | -0.03* | 3.55 | 5.94 |
|  |  | Park |  | -0.30 | -2.71*** | -2.25** | -8.80*** | -7.20*** | -0.26 | -0.07 | -0.03 | 3.57 | 5.95 |
|  |  | Pool |  | -0.28 | -2.79*** | -2.30** | -9.03*** | -7.23*** | -0.35 | -0.09 | 0.01 | 3.57 | 5.96 |
|  |  | Swim |  | -0.29 | -2.88*** | -2.40** | -8.96*** | -7.19*** | -0.42 | -0.09 | 0.01 | 3.59 | 5.96 |
|  |  | Tired |  | -0.29 | -2.76*** | -2.28** | -8.90*** | -7.09*** | -0.27 | -0.07 | 0.01 | 3.58 | 5.95 |
|  |  | water |  | -0.23 | -2.95*** | -2.55** | -9.13*** | -7.19*** | -0.26 | -0.06 | -0.04* | 3.54 | 5.94 |
| Miami-Dade | None | | ARMA(1,1)-GARCH(1,1) | - | - | - | - | - | - | - | - | 26.50 | 9.90 |
|  |  |  |  | -2.71 | -8.15* | -17.74*** | -66.89*** | -74.65*** | 2.93 | - | - | 13.80 | 8.76 |
|  |  |  |  | -2.67 | -7.83* | -17.33*** | -66.13*** | -74.49*** | 3.06 | -0.52 | - | 13.82 | 8.77 |
|  | Twitter | Heat |  | -3.22 | -8.22** | -18.51*** | -66.61*** | -75.00*** | 2.07 | -0.62 | 0.12 | 13.61 | 8.77 |
|  |  | AC |  | -2.88 | -7.75* | -17.84*** | -66.14*** | -74.37*** | 2.50 | -0.58 | 0.25 | 13.76 | 8.78 |
|  | Google Search | AC repair |  | -2.44 | -7.64* | -17.07*** | -65.94*** | -74.11*** | 3.05 | -0.60 | 0.04 | 13.85 | 8.78 |
|  |  | Beer |  | -2.28 | -7.40* | -14.94*** | -61.82*** | -70.53*** | 3.11 | -0.34*** | -0.22*** | 13.70 | 8.76 |
|  |  | Drink |  | -1.74 | -6.72 | -15.57*** | -63.34*** | -71.84*** | 3.36 | -0.73*** | -0.13 | 13.83 | 8.78 |
|  |  | Heat exhaustion |  | -2.79 | -7.77* | -17.01*** | -65.74*** | -74.42*** | 2.99 | -0.53 | 0.02 | 13.82 | 8.78 |
|  |  | Heat stroke |  | -3.40 | -8.24** | -18.13*** | -67.05*** | -73.47*** | 4.40 | -0.31*** | -0.17*** | 14.01 | 8.74 |
|  |  | Hot weather |  | -2.78 | -8.00* | -17.43*** | -66.57*** | -74.56*** | 2.91 | -0.47 | -0.02 | 13.88 | 8.78 |
|  |  | Park |  | -2.05 | -6.60 | -12.24*** | -56.97*** | -67.03*** | 6.01 | -0.17 | -0.59*** | 13.51 | 8.72 |
|  |  | Pool |  | -2.90 | -7.80* | -16.73*** | -62.77*** | -68.87*** | 4.86 | 0.15 | -0.43*** | 13.58 | 8.72 |
|  |  | Swim |  | -2.87 | -8.28** | -17.71*** | -66.44*** | -74.80*** | 2.68 | -0.45 | -0.03 | 13.80 | 8.78 |
|  |  | Tired |  | -3.18 | -8.06* | -17.47*** | -67.12*** | -74.95*** | 2.83 | -0.54 | 0.08 | 13.88 | 8.78 |
|  |  | water |  | -2.43 | -7.72* | -16.60*** | -65.54*** | -73.39*** | 3.68 | -0.47 | -0.19 | 13.68 | 8.78 |
| Orange | None | | ARMA(1,1)-GARCH(1,1) | - | - | - | - | - | - | - | - | 13.70 | 8.60 |
|  |  |  |  | -7.76*** | -8.36*** | -16.00*** | -34.66*** | -33.56* | -0.11 | - | - | 9.14 | 7.91 |
|  |  |  |  | -7.35*** | -7.68*** | -15.44*** | -34.36* | -33.63* | 0.00 | -0.62*** | - | 8.91 | 7.86 |
|  | Twitter | Heat |  | -7.35*** | -7.66*** | -15.43*** | -34.36*** | -33.62*** | 0.01 | -0.64*** | 0.02 | 8.90 | 7.87 |
|  |  | AC |  | -7.19*** | -7.36*** | -15.08*** | -34.11*** | -33.38*** | 0.22 | -0.69*** | 0.20 | 8.92 | 7.87 |
|  | Google Search | AC repair |  | -7.20*** | -7.40*** | -15.87*** | -34.36*** | -33.91*** | 0.00 | -0.55*** | -0.06 | 8.95 | 7.87 |
|  |  | Beer |  | -7.27*** | -7.52*** | -14.37*** | -31.21*** | -30.77*** | -0.15 | -0.55*** | -0.12 | 8.93 | 7.86 |
|  |  | Drink |  | -6.48** | -6.98*** | -14.14*** | -30.05*** | -30.63*** | 0.15 | -0.44*** | -0.19*** | 8.84 | 7.85 |
|  |  | Heat exhaustion |  | -7.30*** | -7.51*** | -15.06*** | -34.06*** | -33.49*** | 0.54 | -0.70*** | 0.05 | 8.94 | 7.86 |
|  |  | Heat stroke |  | -7.71*** | -8.27*** | -15.39*** | -34.45*** | -34.09*** | -0.12 | -0.74*** | 0.07** | 8.86 | 7.86 |
|  |  | Hot weather |  | -7.38*** | -7.63*** | -15.41*** | -34.46*** | -33.65*** | 0.02 | -0.63*** | 0.01 | 8.89 | 7.87 |
|  |  | Park |  | -7.18*** | -7.03*** | -13.12*** | -28.60*** | -31.26*** | 0.81 | -0.43*** | -0.37*** | 8.75 | 7.79 |
|  |  | Pool |  | -7.41*** | -7.46*** | -15.20*** | -32.55*** | -31.62*** | 0.55 | -0.40*** | -0.13** | 8.94 | 7.85 |
|  |  | Swim |  | -7.35*** | -7.98*** | -15.54*** | -34.00*** | -33.34*** | 0.00 | -0.52*** | -0.06 | 8.98 | 7.87 |
|  |  | Tired |  | -7.34*** | -7.68*** | -15.44*** | -34.36*** | -33.63*** | 0.00 | -0.62*** | 0.00 | 8.91 | 7.87 |
|  |  | water |  | -7.35*** | -7.90*** | -15.53*** | -34.07*** | -32.88*** | 0.76 | -0.47*** | -0.13 | 8.98 | 7.87 |

Table S21. Respiratory illness ED model specifications (*** <0.05, ** 0.05 ~ 0.10, * 0.10 ~ 0.15)

|  |  |  | Lag 0 | | | Lag 1 | | | Lag 2 | | | Lag 3 | | |
| --- | --- | --- | --- | --- | --- | --- | --- | --- | --- | --- | --- | --- | --- | --- |
|  | Keywords | | keyword | MAE | AIC | keyword | MAE | AIC | keyword | MAE | AIC | keyword | MAE | AIC |
| Duval | Twitter | Heat | -0.62 | 12.67 | 8.57 | -1.26*** | 12.51 | 8.53 | 0.08 | 12.69 | 8.58 | -0.09 | 12.66 | 8.58 |
|  |  | AC | 0.97 | 12.73 | 8.57 | 0.04 | 12.66 | 8.58 | -1.21 | 12.66 | 8.57 | -0.90 | 12.61 | 8.57 |
|  | Google Search | AC repair | -0.07 | 12.66 | 8.57 | 0.02 | 12.69 | 8.58 | -0.04 | 12.66 | 8.58 | 0.01 | 12.67 | 8.58 |
|  |  | Beer | -0.14** | 12.56 | 8.56 | -0.03 | 12.66 | 8.58 | -0.13** | 12.57 | 8.56 | 0.06 | 12.56 | 8.58 |
|  |  | Drink | 0.06 | 12.71 | 8.57 | 0.11** | 12.66 | 8.56 | -0.08 | 12.61 | 8.57 | 0.06 | 12.59 | 8.57 |
|  |  | Heat exhaustion | 0.00 | 12.66 | 8.58 | -0.04 | 12.70 | 8.58 | -0.01 | 12.65 | 8.58 | -0.01 | 12.64 | 8.58 |
|  |  | Heat stroke | 0.01 | 12.65 | 8.58 | -0.05 | 12.70 | 8.58 | 0.02 | 12.67 | 8.58 | -0.09 | 12.67 | 8.57 |
|  |  | Hot weather | -0.02 | 12.63 | 8.58 | -0.09* | 12.58 | 8.57 | -0.03 | 12.69 | 8.58 | 0.08 | 12.64 | 8.57 |
|  |  | Park | -0.06 | 12.65 | 8.58 | -0.17 | 12.63 | 8.57 | 0.15 | 12.70 | 8.57 | 0.31*** | 12.68 | 8.56 |
|  |  | Pool | 0.00 | 12.66 | 8.58 | -0.10 | 12.58 | 8.57 | 0.06 | 12.63 | 8.58 | -0.10 | 12.66 | 8.57 |
|  |  | Swim | -0.11** | 12.58 | 8.56 | -0.08 | 12.57 | 8.57 | 0.14*** | 12.58 | 8.56 | 0.04 | 12.59 | 8.58 |
|  |  | Tired | -0.11*** | 12.64 | 8.56 | 0.03 | 12.63 | 8.58 | 0.01 | 12.66 | 8.58 | -0.05 | 12.67 | 8.58 |
|  |  | water | 0.07 | 12.70 | 8.58 | -0.02 | 12.66 | 8.58 | -0.07 | 12.74 | 8.58 | -0.15 | 12.56 | 8.57 |
| Hillsborough | Twitter | Heat | -0.31 | 16.29 | 9.01 | 1.03*** | 16.11 | 8.98 | -0.29 | 16.41 | 9.01 | 0.48 | 16.42 | 9.00 |
|  |  | AC | -0.44 | 16.34 | 9.01 | 1.13 | 16.30 | 9.00 | -0.08 | 16.39 | 9.01 | 0.59 | 16.42 | 9.01 |
|  | Google Search | AC repair | 0.09 | 16.33 | 9.00 | 0.02 | 16.38 | 9.01 | 0.09 | 16.35 | 9.00 | 0.11 | 16.31 | 9.00 |
|  |  | Beer | -0.05 | 16.40 | 9.01 | -0.03 | 16.38 | 9.01 | 0.15 | 16.46 | 9.00 | -0.01 | 16.39 | 9.01 |
|  |  | Drink | -0.30*** | 16.19 | 8.98 | 0.03 | 16.41 | 9.01 | 0.06 | 16.43 | 9.02 | -0.03 | 16.40 | 9.01 |
|  |  | Heat exhaustion | -0.05 | 16.35 | 9.01 | 0.06 | 16.38 | 9.01 | 0.03 | 16.39 | 9.01 | -0.10 | 16.34 | 9.00 |
|  |  | Heat stroke | 0.01 | 16.41 | 9.01 | -0.05 | 16.33 | 9.01 | 0.03 | 16.35 | 9.01 | 0.00 | 16.43 | 9.01 |
|  |  | Hot weather | 0.03 | 16.37 | 9.01 | 0.02 | 16.40 | 9.01 | -0.03 | 16.43 | 9.01 | 0.10 | 16.35 | 9.00 |
|  |  | Park | 0.12 | 16.43 | 9.01 | -0.19 | 16.35 | 9.01 | 0.32 | 16.25 | 9.00 | -0.06 | 16.38 | 9.01 |
|  |  | Pool | -0.12 | 16.34 | 9.01 | 0.13 | 16.27 | 9.01 | 0.32** | 16.35 | 9.01 | 0.01 | 16.40 | 9.01 |
|  |  | Swim | -0.20 | 16.35 | 9.00 | 0.03 | 16.41 | 9.01 | -0.13 | 16.34 | 9.01 | -0.06 | 16.41 | 9.01 |
|  |  | Tired | -0.07 | 16.40 | 9.00 | -0.05 | 16.29 | 9.01 | - | - | - | -0.05 | 16.43 | 9.01 |
|  |  | water | 0.37** | 16.23 | 8.99 | -0.14 | 16.36 | 9.01 | 0.21 | 16.34 | 9.00 | -0.19 | 16.36 | 9.00 |
| Leon | Twitter | Heat | 0.08 | 6.91 | 7.36 | -0.03 | 6.90 | 7.36 | -0.14 | 6.89 | 7.36 | 0.19 | 6.88 | 7.36 |
|  |  | AC | 0.88 | 6.86 | 7.35 | -0.72 | 6.91 | 7.36 | 0.65 | 6.86 | 7.36 | -0.70 | 6.89 | 7.36 |
|  | Google Search | AC repair | -0.01 | 6.89 | 7.36 | -0.02 | 6.89 | 7.36 | -0.01 | 6.90 | 7.36 | 0.03 | 6.90 | 7.36 |
|  |  | Beer | 0.03 | 6.87 | 7.36 | - | - | - | -0.04 | 6.88 | 7.36 | -0.03 | 6.91 | 7.36 |
|  |  | Drink | 0.06 | 6.85 | 7.35 | 0.00 | 6.91 | 7.36 | -0.07 | 6.89 | 7.35 | 0.02 | 6.89 | 7.36 |
|  |  | Heat exhaustion | -0.01 | 6.90 | 7.36 | 0.10** | 6.75 | 7.34 | 0.01 | 6.91 | 7.36 | -0.04 | 6.86 | 7.36 |
|  |  | Heat stroke | 0.07** | 6.86 | 7.34 | 0.04 | 6.84 | 7.36 | -0.02 | 6.88 | 7.36 | -0.04 | 6.91 | 7.36 |
|  |  | Hot weather | 0.01 | 6.90 | 7.36 | 0.05* | 6.78 | 7.35 | 0.02 | 6.91 | 7.36 | -0.02 | 6.91 | 7.36 |
|  |  | Park | -0.14*** | 6.82 | 7.34 | -0.04 | 6.90 | 7.36 | -0.04 | 6.89 | 7.36 | -0.08 | 6.88 | 7.36 |
|  |  | Pool | 0.02 | 6.89 | 7.36 | 0.02 | 6.92 | 7.36 | 0.05 | 6.83 | 7.36 | 0.01 | 6.91 | 7.36 |
|  |  | Swim | 0.05 | 6.89 | 7.35 | - | - | - | 0.06* | 6.88 | 7.35 | -0.02 | 6.90 | 7.36 |
|  |  | Tired | 0.00 | 6.90 | 7.36 | -0.01 | 6.91 | 7.36 | -0.07** | 6.77 | 7.34 | 0.03 | 6.87 | 7.36 |
|  |  | water | 0.09** | 6.82 | 7.35 | 0.03 | 6.90 | 7.36 | -0.07 | 6.88 | 7.35 | 0.09* | 6.85 | 7.35 |
| Miami-Dade | Twitter | Heat | 0.07 | 23.07 | 9.74 | -0.08 | 22.96 | 9.74 | -0.11 | 23.13 | 9.74 | -0.05 | 23.12 | 9.75 |
|  |  | AC | -0.19 | 23.04 | 9.75 | 0.68* | 22.81 | 9.73 | 0.35 | 23.03 | 9.74 | -0.19 | 23.04 | 9.75 |
|  | Google Search | AC repair | -0.05 | 23.14 | 9.75 | -0.06 | 23.05 | 9.74 | -0.24*** | 23.00 | 9.72 | 0.01 | 23.09 | 9.75 |
|  |  | Beer | 0.08 | 23.06 | 9.75 | 0.12 | 23.02 | 9.74 | 0.20 | 23.01 | 9.74 | 0.12 | 23.17 | 9.74 |
|  |  | Drink | -0.20 | 23.07 | 9.74 | 0.39*** | 22.75 | 9.72 | 0.32** | 23.02 | 9.73 | 0.01 | 23.11 | 9.75 |
|  |  | Heat exhaustion | -0.06 | 23.15 | 9.74 | -0.21*** | 23.08 | 9.71 | 0.08 | 23.18 | 9.74 | 0.04 | 23.04 | 9.75 |
|  |  | Heat stroke | -0.07 | 23.01 | 9.74 | 0.01 | 23.10 | 9.75 | -0.01 | 23.09 | 9.75 | -0.05 | 23.14 | 9.74 |
|  |  | Hot weather | 0.04 | 23.12 | 9.75 | -0.02 | 23.09 | 9.75 | -0.02 | 23.11 | 9.75 | -0.04 | 23.07 | 9.75 |
|  |  | Park | 0.58* | 22.87 | 9.73 | 0.51* | 22.88 | 9.73 | - | - | - | 0.55* | 23.09 | 9.73 |
|  |  | Pool | 0.03 | 23.10 | 9.75 | 0.21 | 23.04 | 9.74 | 0.27 | 23.08 | 9.74 | 0.22 | 23.15 | 9.74 |
|  |  | Swim | 0.29** | 22.70 | 9.73 | 0.11 | 23.06 | 9.74 | 0.08 | 23.20 | 9.74 | -0.12 | 22.99 | 9.74 |
|  |  | Tired | 0.07 | 23.08 | 9.75 | -0.05 | 23.11 | 9.75 | -0.41*** | 22.67 | 9.71 | -0.05 | 23.13 | 9.75 |
|  |  | Water | 0.40 | 23.14 | 9.74 | -0.20 | 23.09 | 9.74 | -0.04 | 23.08 | 9.75 | 0.15 | 23.17 | 9.75 |
| Orange | Twitter | Heat | 0.22 | 16.74 | 9.12 | -0.08 | 16.98 | 9.12 | 0.25 | 16.80 | 9.12 | 0.06 | 16.97 | 9.12 |
|  |  | AC | 1.58*** | 16.41 | 9.09 | -1.57*** | 16.72 | 9.09 | -0.15 | 17.00 | 9.12 | 0.96* | 16.84 | 9.11 |
|  | Google Search | AC repair | -0.04 | 16.99 | 9.12 | -0.10 | 17.03 | 9.12 | -0.16** | 16.63 | 9.11 | -0.03 | 16.95 | 9.12 |
|  |  | Beer | -0.08 | 16.96 | 9.12 | -0.05 | 16.98 | 9.12 | -0.10 | 16.97 | 9.12 | 0.11 | 16.89 | 9.12 |
|  |  | Drink | -0.23 | 16.78 | 9.11 | -0.10 | 16.94 | 9.12 | 0.05 | 16.96 | 9.12 | 0.21 | 16.84 | 9.12 |
|  |  | Heat exhaustion | 0.07 | 16.86 | 9.12 | 0.04 | 16.96 | 9.12 | -0.01 | 16.97 | 9.12 | -0.16*** | 16.63 | 9.10 |
|  |  | Heat stroke | 0.18*** | 16.61 | 9.10 | -0.12* | 17.05 | 9.11 | -0.11 | 16.91 | 9.11 | 0.01 | 16.98 | 9.12 |
|  |  | Hot weather | -0.09 | 16.81 | 9.11 | 0.06 | 16.87 | 9.12 | 0.04 | 16.95 | 9.12 | 0.10* | 16.85 | 9.11 |
|  |  | Park | -0.31 | 16.85 | 9.12 | -0.36 | 16.93 | 9.12 | 0.57** | 16.61 | 9.11 | 0.23 | 16.92 | 9.12 |
|  |  | Pool | 0.29 | 16.74 | 9.12 | 0.25 | 16.89 | 9.12 | -0.15 | 16.96 | 9.12 | 0.12 | 17.01 | 9.12 |
|  |  | Swim | 0.10 | 16.98 | 9.12 | 0.07 | 16.94 | 9.12 | -0.08 | 16.92 | 9.12 | -0.01 | 16.98 | 9.12 |
|  |  | Tired | 0.06 | 16.97 | 9.12 | -0.18*** | 16.83 | 9.10 | -0.05 | 16.99 | 9.12 | 0.03 | 16.99 | 9.12 |
|  |  | water | 0.09 | 16.97 | 9.12 | -0.13 | 16.94 | 9.12 | -0.19 | 16.99 | 9.12 | -0.07 | 16.95 | 9.12 |

Table S22. Respiratory illness hospitalization model specifications (*** <0.05, ** 0.05 ~ 0.10, * 0.10 ~ 0.15)

|  |  |  | Lag 0 | | | Lag 1 | | | Lag 2 | | | Lag 3 | | |
| --- | --- | --- | --- | --- | --- | --- | --- | --- | --- | --- | --- | --- | --- | --- |
|  | Keywords | | keyword | MAE | AIC | keyword | MAE | AIC | keyword | MAE | AIC | keyword | MAE | AIC |
| Duval | Twitter | Heat | -0.03 | 8.42 | 7.72 | -0.39 | 8.39 | 7.71 | -0.55** | 8.30 | 7.70 | -0.30 | 8.33 | 7.72 |
|  |  | AC | 0.30 | 8.43 | 7.72 | -0.79 | 8.37 | 7.72 | -0.14 | 8.42 | 7.72 | -0.77 | 8.41 | 7.72 |
|  | Google Search | AC repair | 0.05 | 8.38 | 7.72 | -0.06 | 8.38 | 7.71 | -0.03 | 8.43 | 7.72 | -0.04 | 8.43 | 7.72 |
|  |  | Beer | -0.18*** | 8.32 | 7.66 | 0.03 | 8.43 | 7.72 | -0.06 | 8.32 | 7.72 | -0.05 | 8.42 | 7.72 |
|  |  | Drink | -0.08* | 8.40 | 7.71 | -0.09** | 8.22 | 7.70 | 0.06 | 8.35 | 7.71 | -0.04 | 8.41 | 7.72 |
|  |  | Heat exhaustion | -0.01 | 8.41 | 7.72 | -0.09*** | 8.30 | 7.68 | 0.00 | 8.42 | 7.72 | -0.03 | 8.45 | 7.72 |
|  |  | Heat stroke | -0.01 | 8.42 | 7.72 | -0.17 | 8.03 | 7.66 | 0.03 | 8.41 | 7.72 | -0.08* | 8.33 | 7.71 |
|  |  | Hot weather | -0.06* | 8.40 | 7.71 | -0.04 | 8.34 | 7.72 | -0.05 | 8.40 | 7.72 | 0.03 | 8.42 | 7.72 |
|  |  | Park | -0.28*** | 8.10 | 7.67 | -0.09 | 8.39 | 7.72 | -0.20*** | 8.42 | 7.69 | -0.13 | 8.34 | 7.71 |
|  |  | Pool | -0.06 | 8.42 | 7.72 | -0.09** | 8.38 | 7.71 | -0.03 | 8.41 | 7.72 | -0.10** | 8.30 | 7.70 |
|  |  | Swim | -0.05 | 8.44 | 7.72 | 0.00 | 8.42 | 7.72 | 0.00 | 8.42 | 7.72 | 0.01 | 8.43 | 7.72 |
|  |  | Tired | -0.01 | 8.42 | 7.72 | -0.01 | 8.43 | 7.72 | 0.03 | 8.39 | 7.72 | -0.03 | 8.39 | 7.72 |
|  |  | water | -0.25*** | 8.30 | 7.66 | -0.15** | 8.36 | 7.70 | 0.03 | 8.43 | 7.72 | -0.01 | 8.42 | 7.72 |
| Hillsborough | Twitter | Heat | -0.24 | 9.71 | 8.04 | 0.03 | 9.77 | 8.05 | -0.10 | 9.77 | 8.05 | 0.02 | 9.77 | 8.05 |
|  |  | AC | -0.14 | 9.79 | 8.05 | 0.16 | 9.76 | 8.05 | 0.97** | 9.75 | 8.04 | 0.25 | 9.77 | 8.05 |
|  | Google Search | AC repair | 0.01 | 9.76 | 8.05 | 0.05 | 9.79 | 8.05 | 0.02 | 9.76 | 8.05 | 0.04 | 9.80 | 8.05 |
|  |  | Beer | -0.19*** | 9.62 | 8.02 | 0.02 | 9.78 | 8.05 | -0.04 | 9.76 | 8.05 | -0.07 | 9.77 | 8.04 |
|  |  | Drink | -0.17** | 9.62 | 8.03 | 0.17*** | 9.83 | 8.03 | -0.08 | 9.72 | 8.04 | -0.07 | 9.72 | 8.04 |
|  |  | Heat exhaustion | -0.03 | 9.77 | 8.05 | -0.07 | 9.74 | 8.04 | -0.08* | 9.75 | 8.03 | 0.02 | 9.74 | 8.05 |
|  |  | Heat stroke | 0.05 | 9.80 | 8.04 | 0.04 | 9.87 | 8.05 | -0.02 | 9.76 | 8.05 | -0.02 | 9.77 | 8.05 |
|  |  | Hot weather | 0.04 | 9.79 | 8.04 | -0.03 | 9.78 | 8.05 | -0.07 | 9.80 | 8.04 | 0.01 | 9.77 | 8.05 |
|  |  | Park | -0.37*** | 9.34 | 8.00 | -0.13 | 9.68 | 8.04 | 0.05 | 9.74 | 8.05 | 0.06 | 9.77 | 8.05 |
|  |  | Pool | -0.05 | 9.78 | 8.05 | -0.14** | 9.64 | 8.03 | 0.01 | 9.77 | 8.05 | -0.04 | 9.78 | 8.05 |
|  |  | Swim | -0.10 | 9.71 | 8.04 | -0.01 | 9.77 | 8.05 | 0.02 | 9.79 | 8.05 | -0.05 | 9.74 | 8.05 |
|  |  | Tired | 0.03 | 9.80 | 8.05 | -0.05 | 9.74 | 8.04 | 0.09** | 9.77 | 8.03 | -0.07 | 9.74 | 8.04 |
|  |  | water | -0.20* | 9.83 | 8.05 | 0.00 | 9.77 | 8.05 | -0.13 | 9.77 | 8.04 | 0.16 | 9.69 | 8.04 |
| Leon | Twitter | Heat | 0.07 | 3.58 | 5.96 | -0.04 | 3.58 | 5.96 | -0.23** | 3.53 | 5.94 | -0.13 | 3.57 | 5.95 |
|  |  | AC | -0.26 | 3.58 | 5.95 | -0.47 | 3.54 | 5.95 | -0.33 | 3.57 | 5.95 | -0.20 | 3.57 | 5.95 |
|  | Google Search | AC repair | -0.03*** | 3.48 | 5.93 | 0.03* | 3.59 | 5.94 | -0.02 | 3.55 | 5.95 | 0.00 | 3.58 | 5.96 |
|  |  | Beer | -0.03 | 3.56 | 5.95 | -0.02 | 3.55 | 5.95 | 0.02 | 3.55 | 5.95 | -0.01 | 3.57 | 5.96 |
|  |  | Drink | 0.00 | 3.57 | 5.96 | -0.02 | 3.60 | 5.95 | 0.00 | 3.58 | 5.96 | -0.03 | 3.56 | 5.95 |
|  |  | Heat exhaustion | -0.02 | 3.57 | 5.96 | -0.03 | 3.56 | 5.95 | 0.01 | 3.57 | 5.96 | 0.00 | 3.57 | 5.96 |
|  |  | Heat stroke | -0.02 | 3.56 | 5.95 | -0.03 | 3.54 | 5.95 | 0.01 | 3.57 | 5.96 | -0.02 | 3.56 | 5.95 |
|  |  | Hot weather | -0.03* | 3.55 | 5.94 | 0.00 | 3.58 | 5.96 | -0.01 | 3.57 | 5.96 | -0.01 | 3.59 | 5.95 |
|  |  | Park | -0.03 | 3.57 | 5.95 | -0.02 | 3.57 | 5.95 | -0.01 | 3.57 | 5.96 | -0.01 | 3.57 | 5.96 |
|  |  | Pool | 0.01 | 3.57 | 5.96 | -0.02 | 3.54 | 5.95 | -0.01 | 3.56 | 5.96 | -0.03 | 3.54 | 5.95 |
|  |  | Swim | 0.01 | 3.59 | 5.96 | -0.02 | 3.56 | 5.95 | -0.02 | 3.53 | 5.95 | -0.01 | 3.57 | 5.96 |
|  |  | Tired | 0.01 | 3.58 | 5.95 | 0.01 | 3.56 | 5.95 | 0.00 | 3.57 | 5.96 | 0.02 | 3.58 | 5.95 |
|  |  | water | -0.04* | 3.54 | 5.94 | 0.00 | 3.58 | 5.96 | -0.04** | 3.55 | 5.94 | -0.05*** | 3.49 | 5.93 |
| Miami-Dade | Twitter | Heat | 0.12 | 13.61 | 8.77 | -0.01 | 13.83 | 8.78 | 0.11 | 13.74 | 8.77 | -0.01 | 13.82 | 8.78 |
|  |  | AC | 0.25 | 13.76 | 8.78 | 0.22 | 13.82 | 8.78 | 0.00 | 13.83 | 8.78 | 0.02 | 13.83 | 8.78 |
|  | Google Search | AC repair | 0.04 | 13.85 | 8.78 | -0.05 | 13.80 | 8.78 | -0.03 | 13.84 | 8.78 | -0.07 | 13.62 | 8.78 |
|  |  | Beer | -0.22*** | 13.70 | 8.76 | -0.07 | 13.89 | 8.78 | 0.00 | 13.82 | 8.78 | -0.10 | 13.85 | 8.78 |
|  |  | Drink | -0.13 | 13.83 | 8.78 | 0.27*** | 13.87 | 8.75 | -0.33*** | 13.42 | 8.74 | 0.11 | 13.88 | 8.78 |
|  |  | Heat exhaustion | 0.02 | 13.82 | 8.78 | 0.03 | 13.77 | 8.78 | -0.03 | 13.84 | 8.78 | 0.07 | 13.76 | 8.77 |
|  |  | Heat stroke | -0.17*** | 14.01 | 8.74 | -0.02 | 13.82 | 8.78 | 0.03 | 13.86 | 8.78 | -0.04 | 13.77 | 8.78 |
|  |  | Hot weather | -0.02 | 13.88 | 8.78 | 0.05 | 13.85 | 8.78 | -0.11*** | 13.53 | 8.76 | -0.03 | 13.82 | 8.78 |
|  |  | Park | -0.59*** | 13.51 | 8.72 | -0.38*** | 13.73 | 8.75 | -0.23 | 13.67 | 8.77 | -0.10 | 13.84 | 8.78 |
|  |  | Pool | -0.43*** | 13.58 | 8.72 | -0.21** | 13.70 | 8.77 | -0.22** | 13.78 | 8.77 | -0.10 | 13.83 | 8.78 |
|  |  | Swim | -0.03 | 13.80 | 8.78 | -0.20*** | 13.56 | 8.75 | -0.09 | 13.76 | 8.77 | -0.06 | 13.84 | 8.78 |
|  |  | Tired | 0.08 | 13.88 | 8.78 | 0.08 | 13.76 | 8.78 | -0.02 | 13.84 | 8.78 | 0.10 | 13.82 | 8.77 |
|  |  | Water | -0.19 | 13.68 | 8.78 | -0.14 | 13.82 | 8.78 | -0.35** | 13.59 | 8.76 | -0.35** | 13.67 | 8.76 |
| Orange | Twitter | Heat | 0.02 | 8.90 | 7.87 | -0.13 | 8.94 | 7.87 | 0.00 | 8.91 | 7.87 | 0.01 | 8.90 | 7.87 |
|  |  | AC | 0.20 | 8.92 | 7.87 | 0.14 | 8.88 | 7.87 | -0.22 | 8.89 | 7.87 | -0.29 | 8.90 | 7.87 |
|  | Google Search | AC repair | -0.06 | 8.95 | 7.87 | -0.05 | 8.94 | 7.87 | -0.06 | 8.85 | 7.87 | -0.06 | 8.87 | 7.86 |
|  |  | Beer | -0.12 | 8.93 | 7.86 | -0.24*** | 8.83 | 7.84 | -0.11 | 8.93 | 7.86 | 0.02 | 8.90 | 7.87 |
|  |  | Drink | -0.19*** | 8.84 | 7.85 | -0.24*** | 8.85 | 7.82 | 0.05 | 8.92 | 7.87 | -0.14** | 8.71 | 7.86 |
|  |  | Heat exhaustion | 0.05 | 8.94 | 7.86 | -0.01 | 8.91 | 7.87 | -0.04 | 8.92 | 7.87 | -0.06 | 9.01 | 7.87 |
|  |  | Heat stroke | 0.07** | 8.86 | 7.86 | -0.06 | 8.83 | 7.86 | 0.05 | 8.93 | 7.87 | 0.03 | 8.88 | 7.88 |
|  |  | Hot weather | 0.01 | 8.89 | 7.87 | -0.03 | 8.94 | 7.87 | -0.02 | 8.89 | 7.87 | -0.01 | 8.91 | 7.87 |
|  |  | Park | -0.37*** | 8.75 | 7.79 | -0.26*** | 8.97 | 7.84 | -0.11 | 8.92 | 7.87 | -0.20*** | 8.89 | 7.85 |
|  |  | Pool | -0.13** | 8.94 | 7.85 | -0.01 | 8.92 | 7.87 | -0.15*** | 8.86 | 7.85 | -0.10** | 8.91 | 7.86 |
|  |  | Swim | -0.06 | 8.98 | 7.87 | -0.09** | 8.90 | 7.86 | -0.08** | 8.90 | 7.86 | -0.08* | 8.88 | 7.86 |
|  |  | Tired | 0.00 | 8.91 | 7.87 | -0.03 | 8.91 | 7.87 | 0.05 | 8.87 | 7.87 | 0.01 | 8.91 | 7.87 |
|  |  | water | -0.13 | 8.98 | 7.87 | -0.17* | 8.91 | 7.86 | -0.18* | 8.92 | 7.86 | -0.36*** | 8.59 | 7.81 |
